# Design research with the use of visual and symmetry analysis in indigenous woven textiles

**DOI:** 10.1107/S1600576722011153

**Published:** 2023-02-01

**Authors:** Disaya Chudasri, Nattakorn Sukantamala

**Affiliations:** a College of Arts, Media and Technology, Chiang Mai University, Thailand; b Advanced Research Center for Computational Simulation, Chiang Mai University, Thailand; c Department of Mathematics, Faculty of Science, Chiang Mai University, Thailand; Australian Nuclear Science and Technology Organisation, Lucas Heights, Australia

**Keywords:** design visualization, frieze groups, visual anaysis, symmetry analysis, traditional weaving, textile patterns

## Abstract

Since *ca* the 1990s, there have been developments in the weaving process for making *tin chok* fabrics in the Long district, Phrae province, northern Thailand, with the aim of reducing the production time and increasing the production capacity with respect to poverty alleviation and cultural revitalization. In this article, the symmetry patterns in *tin chok* fabrics in a collection of 17 vintage (traditional) skirts held in the Komol Antique Textile Museum, in the Long district, are examined to determine how to distinguish *tin chok* fabrics woven by the integrated method from those woven by the traditional method. The research includes visual and symmetry analysis, literature review, fieldwork, and the creation of a pattern booklet. This research reveals that the hem, which is one of the four parts (supplementary part one, the main part, supplementary part two and the hem) of the detachable *tin chok*, is key to distinguishing the fabrics made by the two methods. The four parts of *tin chok* made by the integrated method will always have a common vertical axis of symmetry, whereas the vertical axes of symmetry of the hem of a fabric made by the traditional method may not be aligned with the motifs in the other three parts. The frieze groups of the individual parts of the 17 vintage (traditional) skirts are decoded and possible corresponding patterns for weaving by the integrated method are generated.

## Abstract 2

This research examines a type of traditional handwoven skirt from northern Thailand, with the aim of distinguishing the fabrics made by the faster integrated method from those made by the traditional method. Visual and symmetry analysis have been employed to inspect the fabric patterns, the design structure and the symmetries.

## 1. Introduction

‘The textile industry is one of global importance, providing high levels of employment, foreign exchange revenue and products essential to human welfare’ [The United Nations Environment Programme (UNEP), 2020 ▸, p. 6]. However, it is notorious for being one of the biggest polluters and is thus harmful to the world, *i.e.* to the environment and human health (UNEP, 2020 ▸, pp. 8, 19–35). It is imperative to change practices in industrial textile production and the consumption of fast fashion toward more sustainable practices, and UNEP has addressed how to create sustainability and circularity in the textile value chain (UNEP, 2020 ▸, pp. 7, 13, 14). This needs a holistic approach and collaborations from multidisciplinary research groups. People face a dilemma with regard to how to move towards such sustainable production and consumption, and what are exemplars. Scholars have suggested various approaches, including material design (*i.e.* fibre types), slow production and responsible consumption, creating a sense of value and care for clothes, and facilitating action towards the 6Rs (refuse, reduce, re-use, repair, repurpose and recycle) along the apparel value chain (UNEP, 2020 ▸, pp. 14, 22, 46, 47). Therefore, we examined a type of traditional skirt made by a slow production method, *i.e.* hand loom weaving, in northern Thailand, with a sense of the sociocultural and economic values, and care for both the making and the use of skirts.

Thailand is well known for its traditional culture and indigenous crafts (World Crafts Council: Asia Pacific Region, 2009 ▸), including handwoven textiles, which are especially evident with respect to the rich cultural heritage of the Thai people (Conway, 1992 ▸, p. 9). Traditional weaving methods have long been handed down through the generations. This rich source of knowledge represents local and national identities (Ministry of Culture, 2009 ▸), and contributes to the soft power (verbal communication with Kosit Panpiemras; Chudasri (2015 ▸) of Thailand. In traditional weaving, weavers do not usually document information about patterning, but they pass on indigenous knowledge from one generation to the next through fabric samples (Conway, 1992 ▸, p. 9), as well as weaving demonstrations and oral instruction. Woven fabrics showing complex patterns are usually kept as a reference (Conway, 1992 ▸, p. 9). In recent decades, the number of weaving communities has been declining due to changes in ways of living. Few young people are being trained in weaving, and experienced weavers are over 40 or 50 years of age. Experiential knowledge in traditional weaving can be permanently lost upon the death of craftspeople if it is not passed on to the next generation. UNESCO recommends that more attention needs to be paid to the continuity of skills and knowledge embedded in craftsmanship, rather than focusing on craft objects themselves [UNESCO (2020 ▸), cited by Zhan *et al.* (2021 ▸), p. 3]. Traditional patterns can reflect some aspects of the past culture as well as the present (Hann, 1992 ▸, p. 581). Traditional patterns refer to the patterns that have been produced using similar techniques for several generations without undergoing significant change, even in recent times (Hann, 1992 ▸, p. 581). Symmetry classification is an analytical tool which anthropologists, archaeologists and design historians use to examine traditional patterns and indicate their cultural adherence, continuity and change (Hann & Thomson, 1992 ▸, p. 55; Hann, 1992 ▸, pp. 581, 589), for example, the use of symmetry to analyse and indicate the designs of artefacts from different cultural settings and historical periods (Hann, 2013 ▸, p. 23). Within any cultural group, there will be a preferred symmetry or symmetries that the group constantly uses in their design systems for the making of objects (Washburn & Crowe, 1988 ▸, p. ix; Hann & Thomson, 1992 ▸, pp. 2, 8, 55). There is a relationship between pattern, symmetry and design for cultural significance. The design structure of patterns and their design elements are meaningful in terms of cultural significance, and they are important criteria for evaluating the overall properties of artefacts. It is critical to enable our understanding of the designs of patterns in correlation with cultural significance (Hann & Thomson, 1992 ▸, p. 55).

### 1.1. A traditional skirt: *sin tin chok*

**Figure 1:**
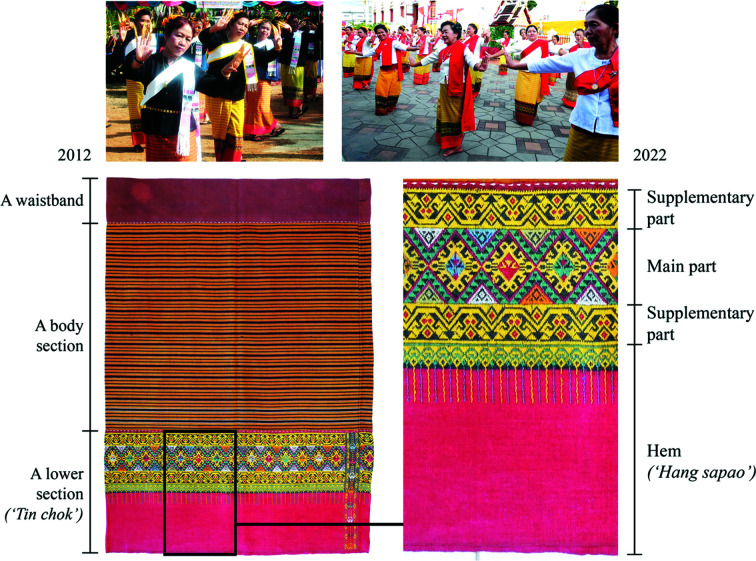
*Sin tin chok* from Komol Phaboraan Museum and the Long people wearing *sin tin chok* for cultural events. Photographs by Disaya Chudasri.

In Thailand, there are a variety of weaving communities, who produce handwoven textiles based on their specialized knowledge and skills. We focus on a representative sample of indigenous textiles from the northern region of Thailand. A majority of the population in northern Thailand are the Tai Yuan, an ethnic Tai group that has been settled in this region for many centuries (Conway, 1992 ▸; Museum of the Bank of Thailand Northern Region Office, 2011 ▸). These days, they call themselves *khon mueang*, which means ‘people of the city’ (Museum of the Bank of Thailand Northern Region Office, 2011 ▸). The Tai Yuan women are specialized in a weaving technique known by the local word *chok*, and they usually produce a specific type of traditional skirt that is known as *sin tin chok* (McIntosh, 2012 ▸). This name comprises three words of different meaning: *sin* is an old Thai word meaning fabric, *tin* means foot or base, and *chok* means an act of picking up and pulling out. In this weave, *chok* means a discontinuous supplementary weft technique (Suchitta, 1989 ▸, p. 97; McIntosh, 2012 ▸). (Information about this weaving technique is expanded in Sections 1.2 and 3.1.) *Sin tin chok* was originally designed to comprise three separate fabrics sewn together, *i.e.* a waistband, a body section and a lower section (Fig. 1 ▸, bottom left). If any of these fabrics were damaged, it could be taken off and replaced with a new one. The lower section is known as *tin chok*, a fabric that was originally designed to comprise four parts: one main part, two supplementary parts and a hem (Fig. 1 ▸, bottom right). Each part in *tin chok* is designed to have complex patterns.

### 1.2. Two methods of *chok* weaving

*Chok* weaving is a discontinuous supplementary weft technique. It is necessary to explain about a related technique, *i.e.* a supplementary weave technique, which provides a foundation for a discontinuous supplementary weave technique.

‘Supplementary warp or weft techniques involve the insertion of additional extra warps or wefts to create designs. These threads are supplementary or extra in the sense that if they were removed the woven cloth would still be complete, though without the original pattern. In the supplementary weft technique, the additional weft threads are made to float over warp threads in a planned sequence to create the design or pattern. If the supplementary threads extend from edge to edge, they are ‘continuous’, but if they only go back and forth in small areas where they are needed, they are ‘discontinuous’’ (De Las Peñas *et al.*, 2018 ▸).

In northern Thailand, one can still find weaving communities that make *sin tin chok* because they recognize it as their intangible cultural heritage. In this work, we focus on *sin tin chok* from the Long district, Phrae province, because the weavers are renowned nationally and there have been developments in the weaving process for *tin chok* (Chudasri, 2015 ▸, pp. 174–176). Nevertheless, the woven patterns have not generally undergone significant change, even in recent times. Although the supplementary weave can be of the warp or of the weft, in this *chok* weaving, the Long weavers insert the supplementary threads on the weft. The traditional loom for making *tin chok* appears to have the minimum — just two shafts. Without hand manipulation of warp threads, the only thing it will produce is plain weave, one over and one under. However, with hand manipulation, weavers can create many patterns, either simple or complex. To make *tin chok* on a loom equipped with just two shafts, weavers have to repeatedly hand pick the sequences of the warp threads to create the patterns. This is a time-consuming process, but the woven fabric can be of exceptionally high quality on both the front and the reverse of the fabric if the weaver knots the additional weft threads along the woven fabric (Chudasri, 2015 ▸, pp. 174–176). In this article, we refer to this method as ‘the traditional method’.

Since *ca* the 1990s, the Long weavers have modified part of the traditional method with the aim of reducing the production time and increasing the production capacity (Chudasri, 2015 ▸, pp. 174–176). Hence, they have integrated a brocade technique (known locally as *yok dok*) with a discontinuous supplementary weft technique (*chok*). There is an additional task to be done prior to weaving, *i.e.* to make a device for the prearrangement of threads. Although making this device takes some time and needs highly skilled weavers, it is worth doing because this device can help the weavers speed up the weaving process. This device contains multiple shafts of threaded heddles that hold sequences of the warp threads, and the master weave to guide weaving. Next, this device is fitted to the loom to help control the sequences of warp threads to raise or lower at the same time. Thus, the weaver can create patterns without estimating the warp threads each time. Moreover, *chok* weaving can be done faster by less skilful weavers. In this article, we refer to this weaving method as ‘the integrated method’. *Tin chok* from the Long district since *ca* the 1990s are made by the integrated method.

Here we raise a question: How can one distinguish *tin chok* fabrics made by the ‘traditional method’ from those made by the ‘integrated method’? In this article, we consider symmetry as an analytical tool to examine the traditional patterns. The weaving master of the Long district advised us to look at the vertical axes of symmetry of the pattern fabrics. If they were made by the integrated method, there would be a common vertical axis of symmetry along the four parts of the pattern (supplementary part, main part, supplementary part and hem). They are altered with resepct to the vintage fabrics, in which the symmetry axes are not aligned. De Las Peñas *et al.* (2018 ▸) also indicate that ‘the types of symmetries and colour symmetries produced are related to the weaving techniques and the cultures that produced them.’ The aim of this study was to analyse a representative sample of the Long indigenous textiles in order to capture the ranges of symmetries and indicate relationships between the symmetries and the weaving methods with respect to the Long textile. (Colour symmetries are not considered in this study. This may raise a question in one’s mind as to why colour can be ignored. This is because the weavers can easily and randomly use different colours of threads in the pattern elements related by symmetry. This flexibility is applicable to the traditional method, as well as to the integrated method. Fig. 7 below is an obvious example of this.)

The article is organized as follows: In Section 2, we describe the collection of 17 vintage (traditional) skirts to be studied and the analysis methods, including the alterations of the patterns woven by the traditional method when reproduced by the integrated method and subsequent symmetry analysis. In Section 3, we describe the integrated method and the two techniques for handling the weft threads, affirm that the hem is key to distinguishing the fabrics made by the two methods, and present the decoding of the symmetry analysis of the 17 traditional skirts and the corresponding patterns generated for weaving by the integrated method. We also present symmetry classifications identified from every pattern, and discuss the two approaches by which fabric 14 can be woven by the integrated method. We conclude with a summary of the five main findings.

## 2. Materials and methods

### 2.1. Materials

**Figure 2:**
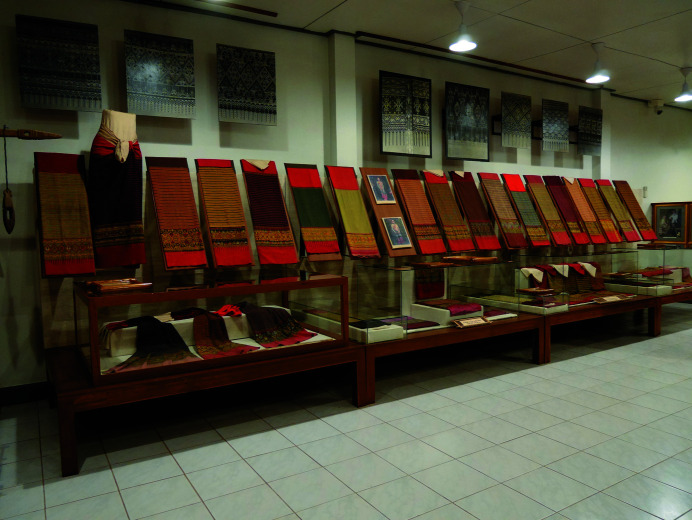
Collection of vintage skirts exhibited in Komol Phaboraan Museum. Photograph by Disaya Chudasri.

Since the Long weavers are familiar with the integrated method for *chok* weaving, they prefer not to employ the traditional method. It is important that the indigenous samples must be carefully identified. Vintage clothes, including *sin tin chok*, can be found in local museums and temples. In this work, we examine an indigenous collection of *sin tin chok* exhibited in the Komol Phaboraan Museum (Komol Antique Textile Museum) located in the Long district. This textile collection comprises 17 vintage skirts (Fig. 2 ▸) that are said to be woven using the traditional method. They are all estimated to be more than 100 years old, determined from the time when Komol received the fabrics from the donors and their oral information, together with the age of the museum’s owner.

### 2.2. Visual analysis

**Figure 3:**
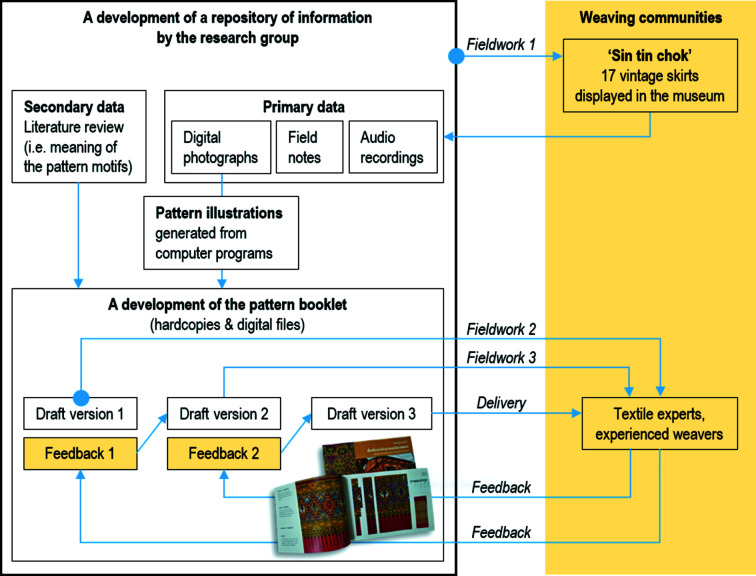
Data collection and visualization of information in 2018.

Visual analysis is a method of understanding art/an artwork, including its visual structure, composition and elements, such as point, line, shape, symmetry, colour and direction, and of explaining how the artist works out how to create meaning (Johnson Museum of Art, 2022 ▸). Practicing visual analysis sharpens critical judgment skills and helps people seek out answers instead of passively receiving information (Johnson Museum of Art, 2022 ▸). The use of visual analysis can help people to recognize the choices that an artist makes in creating the artwork (Johnson Museum of Art, 2022 ▸). Visual analysis is also applicable to other areas in which people read and critically interpret images or artefacts, for example, woven textiles displayed in museums. In 2018, a research group was set up, including the first author and six undergraduate students from Chiang Mai University. The group conducted fieldwork in the Long district three times, every three months, and each visit lasted two days. It was mainly based in the museum and the weaving communities, where the research group got permission to discuss information, review artefacts and take photographs. Creating a pattern booklet (Chudasri, 2018 ▸) is part of the research, as the Long weavers addressed the significance of a book in relation to knowledge transfer (Chudasri *et al.*, 2020 ▸), for example, between local weavers and visitors (*e.g.* teachers, students and tourists). The research process is illustrated in Fig. 3 ▸.

After *Fieldwork-1*, a repository of information was created in a computer system, using the internet and cloud storage to store and share information within the research group. The research group conducted visual analysis by looking at clear and vivid photographs of the 17 fabrics. With a basic knowledge of symmetry, the research group identified shape boundaries of motifs in every pattern. These shapes are rectangles and vertical axes of symmetry are drawn to divide the shapes into half motifs. Although colour symmetries are not considered in this study, colour has been used in the visual analysis to identify the boundaries of each shape element in the patterns. The pattern elements were decoded and drawn on graph paper (Chudasri, 2019 ▸, p. 192; this paper is available as supporting information). It was essential to refer back repeatedly to the original photographs of the 17 vintage fabrics (available in the repository of information and the pattern booklet) when decoding and regenerating the patterns to avoid distortion of information. For example, one must avoid introducing a preconceived alignment of vertical axes of symmetry across the four parts of the patterns. Next, the research assistants used computer software to digitally record information from the decoding of patterns. The working files in *Excel* were saved as images and transferred to *Photoshop* for generating patterns with tools (*i.e.* Select > Transform Selection, Image > Image Rotation, Edit > Transform). These illustrations were ready for placing in the pattern booklet. Additionally, a literature review was con­ducted via web-based information to find out about pattern descriptions and meaning. In this research, the pattern booklet refers to a thin document (54 pages) that was written in the central Thai language and which provides information about the design structure of *sin tin chok* and the 17 vintage skirts (Fig. 2 ▸), photographs of their decorative patterns, illustrations of these patterns plotted on graphs (both the vintage fabrics and the modified integrated patterns), and descriptions and meanings of the pattern motifs. The development of the pattern booklet is a co-creation and iterative process that incorporates recommendations from the Long people (Fig. 3 ▸).

The research group came up with the first draft of the pattern booklet that contained visual information and initial symmetry analysis of 11 fabrics, along with text-based information that described the pattern names and meaning. In *Fieldwork-2*, the first draft was presented to the weaving experts of the Long district for discussion and recommendations. The weaving experts pointed out a few errors in the supplementary parts of the pattern visualization, notably incorrect identification of twofold rotation axes and boundaries of asymmetric units in the patterns, where the motifs did not appear exactly the same as in the vintage fabrics. (Thereafter, the research group corrected these errors in a revised version of the booklet.) Nevertheless, the weaving experts of the Long district were satisfied with the first draft and looked forward to the analysis of all 17 fabrics. They men­tioned further that, if a given pattern was woven by the integrated method, the vertical axis of symmetry of the four parts (supplementary part, main part, supplementary part and hem) must be aligned. Therefore, the research group did more work on alterations of the symmetry axes. (Section 2.2.1 explains how the traditional patterns can be altered to obtain the integrated patterns.) This resulted in two series of symmetry axes that were based on the 17 vintage fabrics (Figs. 8 and 9). The two series of symmetry axes were placed in the second draft and this was presented to the weaving experts in *Fieldwork-3*. Thereafter, the research group amended the detailed information on the basis of the experts’ recommendations, and this turned into the third draft, which is an effective tool for us to inspect the patterns and discuss with experts in symmetry analysis (Section 2.3).

#### 2.2.1. Alterations in the traditional patterns to obtain the integrated patterns

**Figure 4:**
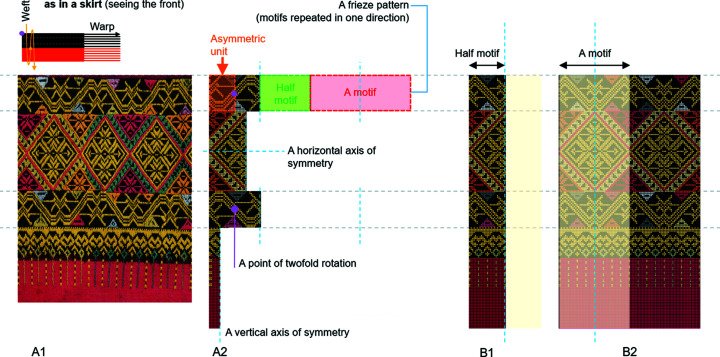
Visual analysis of vintage fabric 2 (A1 and A2) and the alterations of the symmetry axes to adapt the pattern to the integrated method (B1 and B2). Image by Disaya Chudasri.

In Fig. 4 ▸, A1 is a photograph of the vintage fabric (fabric 2 in Fig. 8) and A2 is a computer-generated image of the patterns decoded from A1. In A2, there are descriptors and guidelines for visual and symmetry analysis. A pattern is a set of lines, shapes or colours that are designed and arranged repeatedly in an orderly sequence (Washburn & Crowe, 1988 ▸, p. 8). A pattern comprises the repetition of a motif or motifs at regular intervals. All patterns can be obtained from the translation of the motif(s) along the straight line parallel to the direction of the warp yarn. This straight line is called the axis of the pattern. Here, a line parallel to the axis of the pattern will be called a horizontal line; a line perpendicular to the axis of the pattern will be called a vertical line. When a pattern is cut exactly along a vertical line so that the two parts are mirror images of each other, then the pattern is said to be vertically symmetric. That vertical line is called the vertical axis of symmetry. Likewise, when a pattern is cut along a horizontal line so that the two parts are mirror images of each other, then the pattern is said to be horizontally symmetric. That horizontal line is called the horizontal axis of symmetry. In addition, if a pattern can be rotated by 180° about one fixed point to obtain a result identical to the pattern itself, then the pattern is said to be twofold rotationally symmetric. Here, that fixed point is called a point of twofold rotational symmetry. The motif will be called a ‘symmetric motif’ if there exists either a vertical axis of symmetry or a horizontal axis of symmetry or a point of twofold rotational symmetry. Otherwise, it is said to be an ‘asymmetric motif’. Therefore, a motif can be either symmetric or asymmetric. For a symmetric motif with a vertical axis of symmetry, the vertical half is called a ‘half motif’. It is clear that every asymmetric motif is the minimal pattern in the context of a frieze pattern design. Thus, it will be referred to as the ‘asymmetric unit’. Every symmetric motif consists of at least two asymmetric units of the same size, shape and content (Hann & Thomson, 1992 ▸, pp. 1–2). In these 17 fabrics, every motif is a building block that is regularly repeated in one direction across the plane, and the result of this is a frieze pattern. In mathematics, a frieze pattern is a two-dimensional design that repeats in one direction. In Fig. 4 ▸, B1 and B2 visualize the patterns as if they were woven by the integrated method.

The numbers of warp threads and/or weft threads were modified, so every half motif (from A2) has the same distance along the warp, and they are integrated into one unit – that is another half motif, as shown in B1. This integrated half motif is repeated evenly by reflection along the warp, creating the frieze pattern, as shown in B2.

### 2.3. Symmetry analysis

In mathematics, a frieze pattern is a two-dimensional design that repeats in one direction. This corresponds to patterns in *tin chok* fabrics. They are designed in two dimensions (length and width) and they are woven incrementally in one direction that increases the length of the fabric. All frieze patterns can be classified into seven groups on the basis of their symmetries (Bart & Clair, 2022 ▸): these are known as *frieze groups*. A frieze pattern is generated as a result of movement of the original motif in the plane in one direction – this is known as rigid motion (Hann, 2013 ▸, p. 24).

**Figure 5:**
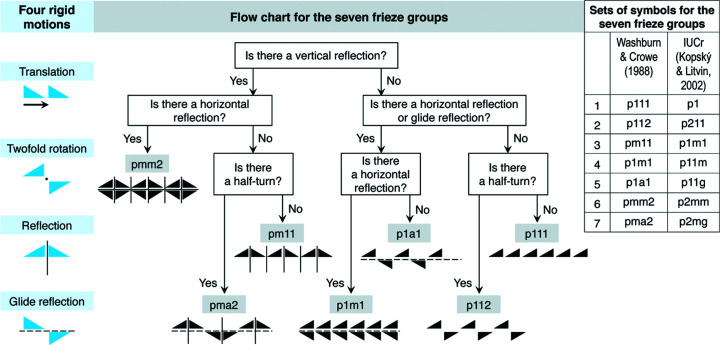
Rigid motions and a flow chart to classify the seven frieze groups. Illustration by Disaya Chudasri, after Washburn & Crowe (1988 ▸).

*A rigid motion* is a transformation in plane in which the original motif and its image are congruent in size, shape and content (Shubnikov & Koptsik, 1974 ▸, p. 159). The four kinds of rigid motions consist of a translation, a twofold rotation, a reflection and a glide reflection (Fig. 5 ▸, left), which can generate any pattern (Shubnikov & Koptsik, 1974 ▸, p. 159; Hann, 2013 ▸, p. 24). When rigid motions are combined in designs which repeat or translate in one direction, a total of seven frieze groups can be produced according to their symmetries (Fig. 5 ▸) (Washburn & Crowe, 1988 ▸, pp. 44, 58–59, 83; Hann, 2013 ▸, pp. 24–27). In this article, we employed the methodology and crystallographic symbols of Washburn & Crowe (1988 ▸) to classify the seven frieze groups. We also compare these symbols with those of the International Union of Crystallography (IUCr) (Kopský & Litvin, 2002 ▸) (Fig. 5 ▸, right).

*The seven frieze groups* refer to classification codes, each of which comprises four characters from the set *p*, *m*, *a*, 1 and 2 (Fig. 5 ▸). These classification codes give the complete information about the mathematical symmetry elements of such patterns. The first character for any code of the seven frieze groups is always a letter ‘*p*’. For the second character, if the pattern shows a vertical axis of symmetry, it will be a letter ‘*m*’. Otherwise, it is a number ‘1’. The third character provides information about the horizontal/glide symmetry of the pattern. It will be a letter ‘*m*’ if the pattern presents a horizontal axis of symmetry, and it will be a letter ‘*a*’ if the pattern has a glide reflection but not a horizontal axis of symmetry. Otherwise, it is a number ‘1’. Finally, the fourth character will be a number ‘2’ if the pattern is of twofold rotational sym­metry. Otherwise, it is a number ‘1’. Of the four characters in the code of a frieze group, the first character has to be a letter ‘*p*’. The second character could be either a letter ‘*m*’ or a number ‘1’. The third character is allowed to be a choice of ‘*m*’ or ‘*a*’ or ‘1’. The last character can be a ‘1’ or ‘2’. By combinatorial methods, we obtain 12 different codes as follows: *pmm*1, *pmm*2, *pma*1, *pma*2, *pm*11, *pm*12, *p*1*m*1, *p*1*m*2, *p*1*a*1, *p*1*a*2, *p*111 and *p*112. In the case that the second and third characters are ‘*mm*’, the pattern shows both vertical and horizontal symmetry. This automatically results in twofold rotational symmetry. Hence, the *pmm*1 type does not exist. Similarly, none of *pma*1, *pm*12, *p*1*m*2 and *p*1*a*2 exist. Therefore, the seven frieze groups are as follows: *pmm*2, *pma*2, *pm*11, *p*1*m*1, *p*1*a*1, *p*111 and *p*112.

We applied this knowledge about the seven frieze groups to inspect *chok* patterns and verify whether their vertical axes of symmetry can really distinguish *tin chok* fabrics made by the traditional method from those made by the integrated method. In identifying symmetry classes in patterns, we strongly suggest considering motifs that are regularly repeated in the overall design of the frieze patterns. One can mistakenly identify symmetry classes if only looking at asymmetric units and/or half motifs, and not taking account of motion that implies actual movement along the warp. Misinformed or incomplete information may lead to the incorrect identification of symmetry classes, and thereby different patterns. Furthermore, the image quality of the patterns should be clear and obtained in high resolution for enlargement. Additionally, it was very helpful to discuss with experts, who have sufficient knowledge in traditional arts and have an eye for detail. In this research, they included a designer, a mathematician, an archaeologist/anthropologist/expert in symmetry analysis and weavers.

## 3. Findings

### 3.1. The integrated method and the two techniques for handling the weft threads

**Figure 6:**
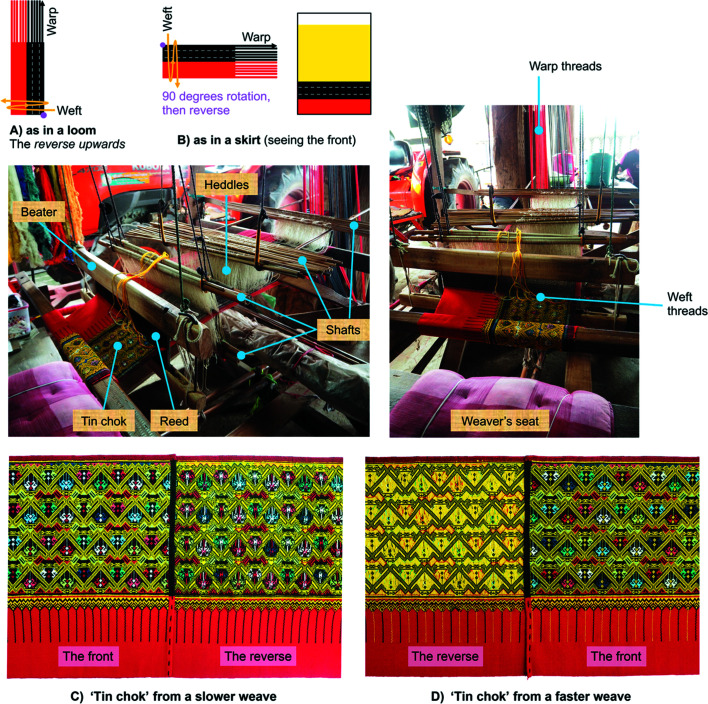
The floor loom set for weaving *tin chok* with the integrated method in the Long district. Photographs by Disaya Chudasri.

The Long weavers carry out their weaving on a floor loom, and they prefer the integrated method for *chok* weaving (Fig. 6 ▸). It is necessary to explain about textile setting in a loom for weaving by the integrated method, because the placement of a *tin chok* fabric when it is woven on the loom is different from when it is sewn to the skirt. There are human interactions involved. The Long weavers usually weave the reverse of *tin chok* upwards, with the front underneath. (In other provinces, weavers may place the front of *tin chok* the other way round.) On the loom, the width of *tin chok* appears in a horizontal orientation parallel to the weaver and the pattern runs vertically up the warp [Fig. 6 ▸(*a*)]. In the traditional skirt, *tin chok* is turned through 90° and is reversed to show its front in the lower section of the skirt [Fig. 6 ▸(*b*)]. In this loom, the width of a fabric is controlled by a reed and the length is set on warp threads attached to the loom. This set of threads remains in place during the entire weaving process until the woven fabric is complete. Whereas two front shafts produce underlying plain weave, the other shafts behind them create patterns. Multiple shafts of threaded heddles control the sequences of the warp threads, raising or lowering them at a particular time to create sheds (gaps), so a shuttle carrying the weft threads can be passed through to form lines/rows of patterns. In each row, the supplementary weft threads may be woven to float over the warp threads for a shorter distance or a much longer distance, depending on the technique of the weaver, the pattern design and the colour. Since these supplementary weft threads are discontinuous, it is possible to produce pattern designs that change colour^1^ from one motif to another across the fabric width and the entire warp (De Las Peñas *et al.*, 2018 ▸, p. 463). As a result, the fabric can be very colourful. With a deep understanding, one can correctly identify the direction in which the pattern progresses and draw vertical or horizontal axes, hence the validity of symmetry classifications in relation to the weaving methods.

In this fieldwork, we find that there are two techniques that the weavers employ to handle the supplementary weft threads: a slower weave [Fig. 6 ▸(*c*)] and a faster weave [Fig. 6 ▸(*d*)]. There are several factors that the weavers consider to determine which technique to implement. These include effects on the fabric properties (*i.e.* aesthetics and durability), the production duration, prices of the fabrics, pattern designs and colours, the ability and availability of the weaver, and the customer preference. In a faster weave, with a sample fabric shown in Fig. 6 ▸(*d*), the weaver inserted the supplementary weft threads (*e.g.* yellow and red) through the gaps for much longer distances. Additional supplementary weft threads were also inserted for shorter distances to create colourful elements in the pattern. The fabric was finished in a shorter time, but the reverse of the fabric shows several areas where the weft threads are floating for long distances. In a slower weave [Fig. 6 ▸(*c*)], the weaver inserts the supplementary weft threads through a few sequences of the warp threads to form lines of the pattern, and hand picks these threads, which may be cut or knotted. The weaver keeps doing this for the entire width of the fabric. Although, the weaver needs more time for weaving, the fabric can be of exceptionally high quality on both the front and the reverse of the fabric. In terms of pricing, weavers sell fabrics woven by the faster-weave technique at a lower price than those produced by the slower-weave technique.

### 3.2. The hem, a key for distinguishing the fabrics woven by the two methods

**Figure 7:**
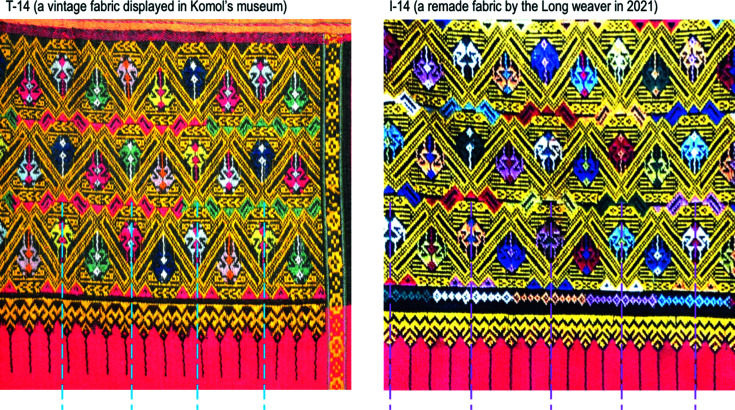
The hems identified with the vertical axes of symmetry relative to the translation distance of the main motifs in fabric 14. Image by Disaya Chudasri.

**Figure 8:**
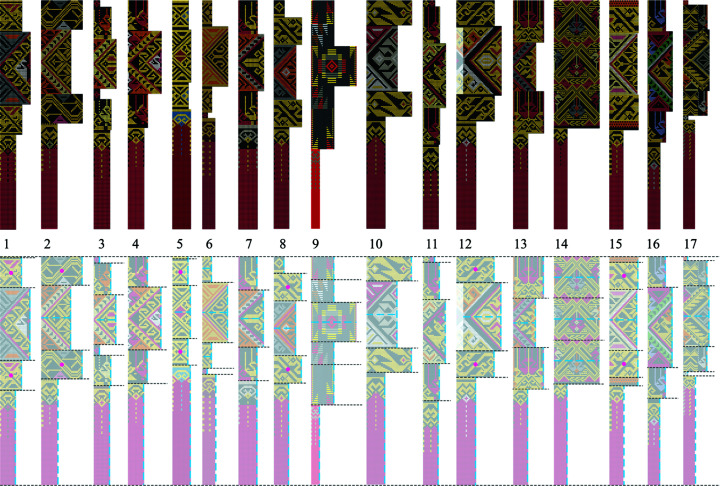
Half motifs and axes of symmetry decoded from the 17 vintage fabrics. [It is noted that some fabrics contain additional parts with much simpler patterns than the other parts (*e.g.* top of T1, top and middle of T3, top of T5, above the hem of T6, top of T8, top of T11, top and above the hem of T15, and top and above the hem of T17). These additional parts are transferred to the integrated patterns in the same way as the other parts.] Dashed blue lines indicate axes of symmetry (horizontal and vertical) and red dots indicate twofold rotation axes in each part. Image by Disaya Chudasri.

With reference to the pattern booklet (Chudasri, 2018 ▸), we carefully inspected photographs of the 17 vintage fabrics. We found a variety of motif designs that appear in their main parts and supplementary parts. In every fabric, motifs in the two supplementary parts were of the same design, or they alternated, whereas motifs in all of the hems are of a similar design. The design contains, in particular, shape elements that look like a letter ‘M’ or ‘E’, a diamond, and dashed lines, all of which are usually repeated on the red fabric. This common design is called *hang sapao*. It is unique and represents the identity of *tin chok*. Having considered photographs of each vintage fabric, we confirm that the vertical axes of symmetry of the motifs in the main part, the pair of supplementary parts and the hem are *not* aligned with those in the adjacent part(s), but this is not the case for fabric 14. Fabric 14 is distinct from the others because only one motif design was executed and arranged evenly throughout the main part and the supplementary parts of the fabric. In this motif, there is a horizontal glide reflection axis, but no vertical reflection axis (Fig. 8, illustration 14). Although this motif design was woven neatly and arranged evenly with the same distance of translation, the vertical axes of symmetry of the hem are *not* evenly aligned with the translation distance of such a motif. Therefore, we affirm that the hem is key to clearly distinguishing the woven fabrics of the traditional method (Fig. 7 ▸, T-14) from those of the integrated method (Fig. 7 ▸, I-14). (Further information about the fabrics made by the integrated method is given in Sections 3.3.2, 3.4 and 3.5.) Additionally, we must emphasize that it is very important to consider the overall design of each fabric and that it contains at least three repeats of motifs in the frieze pattern(s).

Why were the vertical axes of symmetry in the motifs not aligned with those in the adjacent part? The weavers res­ponded that, in the traditional weaving method (Section 1.2), the weavers designed a pattern from their imagination and memory, and executed it directly on the loom [Chudasri (2015 ▸), and a personal communication with weavers in 2022]. They used a porcupine quill to freely pick the warp threads up or push them down along each part of the pattern, and inserted the weft threads to create the pattern elements. At the beginning of a fabric, they usually recalled or developed shape elements or changed their mind about colours, and they carried on weaving even when they made a fairly small mistake. It was all about happiness that they could freely think and do it directly on the loom. They preferred not to start all over again because *chok* weaving is a time-consuming pro­cess, and they did not want to cut the threads or create waste. Moreover, each weaver had a personal way of re­mem­bering things, so it was not necessary that every weaver would start each part of a pattern at the centre or at the rim (*e.g.* fabric 12 in Fig. 8).

### 3.3. The decoding of the symmetry analysis (half motifs and symmetries)

**Figure 9:**
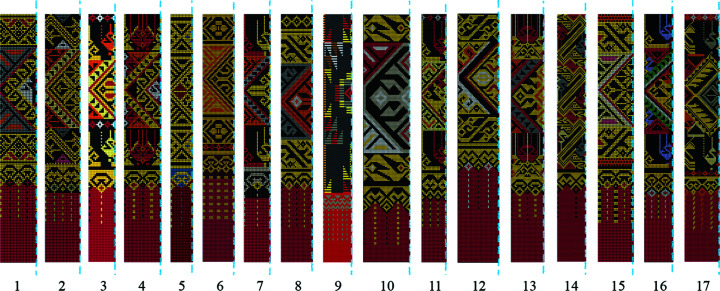
The half motifs generated for patterning the 17 fabrics by the integrated method. Dashed blue lines indicate the vertical reflection axes common to all parts in each pattern. Image by Disaya Chudasri.

In this section, we answer the question of how the traditional patterns can be altered to obtain the integrated patterns (Section 2.2.1). Two series of patterns are illustrated. Series 1 depicts half motifs and axes of symmetry decoded from the 17 vintage fabrics (Fig. 8). Series 2 depicts half motifs and axes of symmetry modified for the integrated method (Fig. 9).

#### 3.3.1. Series 1: half motifs and axes of symmetry decoded from the 17 vintage fabrics

In general, the vertical axes of symmetry in the main part, the pair of supplementary parts and the hem of each fabric were not aligned evenly throughout (Fig. 8 ▸). Fabrics 9, 12 and 14 need further explanation. In fabric 12, although the decoding of the main part and the supplementary parts show the same widths of half motifs, the vertical axis of the main part in the vintage fabric was offset from the vertical axes of the supplementary parts. In fabric 9, the main part does not have a vertical axis of symmetry, but does have a horizontal reflection axis. In fabric 14, only one motif design was used in the main part and the supplementary parts, and this motif does not have a vertical axis of symmetry. We have explained further about fabric 14 in Sections 3.2 and 3.5.

#### 3.3.2. Series 2: half motifs and axes of symmetry modified for the integrated method

Based on Series 1, each individual part in each fabric was modified with respect to the numbers of warp threads and/or weft threads, so their widths along the warp are congruent. These four parts show an equal alignment, with only one vertical axis of symmetry passing through them. They are integrated into a half motif that will be moved on its vertical reflection axis to create a frieze pattern along the warp. In these alterations, the widths of the supplementary parts and the hems in Series 1 (Fig. 8 ▸) may be fractions or the whole of the width of the half motifs in Series 2 (Fig. 9 ▸). They were modified for the integrated method. For example, in Fig. 9 ▸ (illustrations 1, 4 and 6), the supplementary parts are fractions of the width of the half motifs, whereas in illustration 14, the supplementary parts are the whole of the width of the half motifs.

Fig. 9 ▸ demonstrates that the construction of the integrated versions based on the asymmetric units of the traditional versions (Fig. 8 ▸) will always possess a vertical reflection axis. The presence of the vertical reflection axis is obvious in all the integrated versions of the 17 patterns, including pattern 14, even if its traditional version did not have a vertical reflection axis.

### 3.4. Symmetry classifications

**Figure 10:**
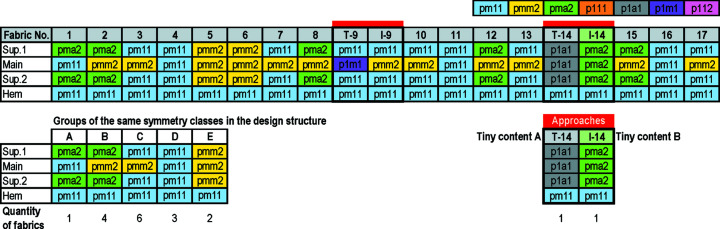
Symmetry classification identified from the 17 fabrics and the 17 modified patterns.

**Figure 11:**
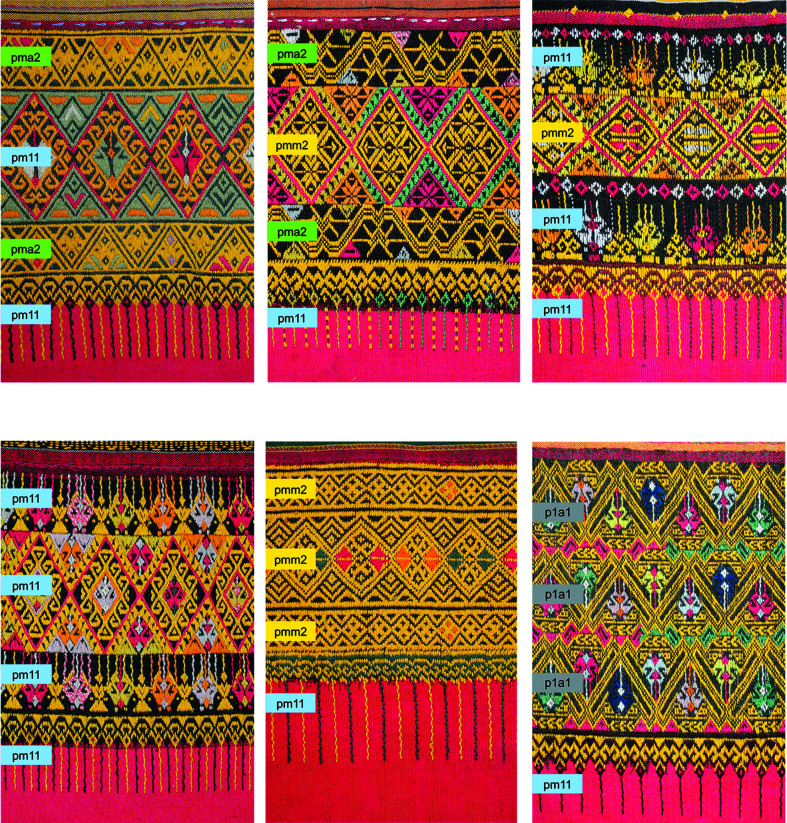
Patterns identified with symmetry classes in relation to design structure. Image by Disaya Chudasri in 2018.

Regarding the four rigid motions, motifs in the 17 vintage fabrics were generated from reflection, twofold rotation, translation and glide reflection. Five of the seven frieze groups appeared, namely, *pm*11, *pmm*2, *pma*2, *p*1*a*1 and *p*1*m*1 (Fig 10 ▸). Regarding the design structure of the fabrics, in the main part, *pmm*2 was often used (11 motifs), *pm*11 was sometimes used (4 motifs), and *p*1*a*1 and *p*1*m*1 appeared just once. In the supplementary parts, *pm*11 was often used (9 motifs), *pma*2 was used sometimes (5 motifs), *pmm*2 was used in two motifs and *p*1*a*1 appeared just once. Patterns in the hems were generated from *pm*11. The symmetry classes identified from the 17 vintage fabrics and the 17 modified patterns are the same, except for fabric 9 [Fig 10 ▸: T-9 (vintage) and I-9 (modified)] and fabric 14 [Fig 10 ▸: T-14 (vintage) and I-14 (modified)]. In the main part of fabric 9, the rectangular shape elements are translated to be vertically and horizontally aligned within its part. Thus, its vertical axis of symmetry is connected to the vertical axes of symmetry in the other three parts. Fabric 14 is distinct from the others in that only one motif was executed across the main part and the supplementary parts. *p*1*a*1 was identified in the vintage fabric (T-14), whereas *pma*2 was identified as the modified pattern (I-14). (Section 3.5 discusses fabric 14 in more detail.) These 17 vintage fabrics can be sorted into six groups according to their symmetry classes in the design structure (Fig 10 ▸, A–E and T-14). Fig. 11 ▸ exemplifies the vintage fabrics from these six groups.

### 3.5. Fabric 14: design alterations with technical constraints

**Figure 12:**
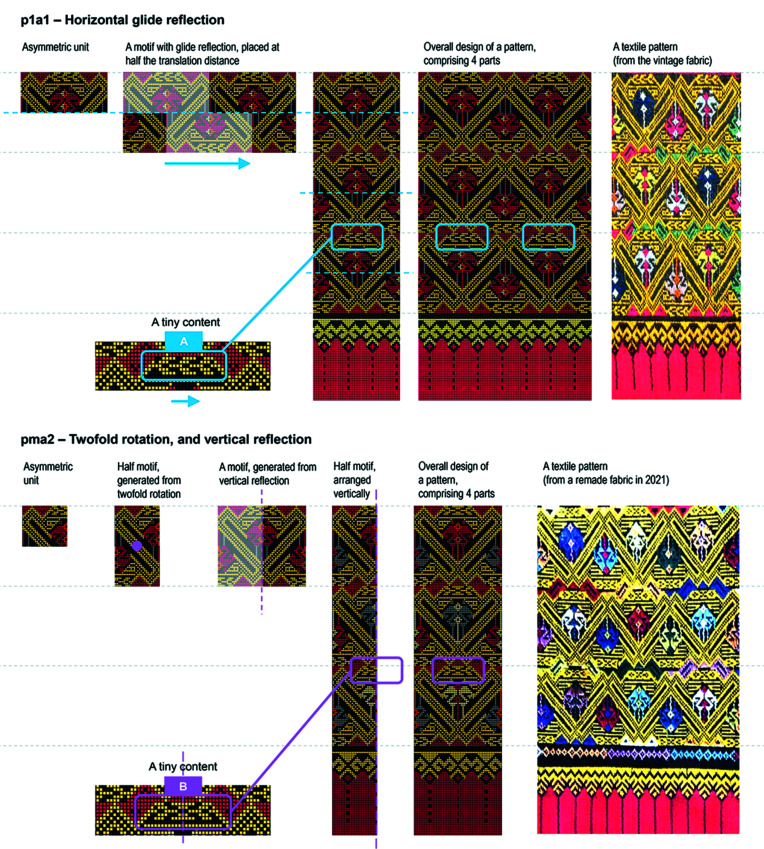
Symmetries in pattern 14 and design alteration due to technical constraint. Image by Disaya Chudasri.

In fabric 14, only one motif was executed repeatedly across the main part and the supplementary parts. In the traditional version, this motif has a horizontal glide reflection, and it is placed at half the translation distance (*p*1*a*1). In the integrated version, the motif has twofold rotation and a vertical reflection (*pma*2). We found that different symmetry classes resulted in different designs (Sukantamala, 2012 ▸, pp. 16, 271). We com­pared the illustrations between the traditional version and the integrated version of fabric 14 and found small details showing a minor difference (Fig. 12 ▸, ‘tiny content’ sections A and B). At that time, we had the photographs of the real/vintage fabric 14, but we had not seen the real/remade fabric by the integrated method. Thus, we wondered what the pattern would look like in reality and which approach the weaver preferred, A *p*1*a*1 or B *pma*2. Would the weaver adopt approach A in a loom set up for the integrated method? We thought that for approach A it would only take a little more time to prearrange a patterning device, but it would not take more time in the next step of weaving. Thus, we asked the weaver and probed into this issue. We were very fortunate that the weaver had just remade a fabric in 2021 – and it was fabric 14 by the integrated method. The weaver also provided photographs of this remade fabric for use in this article (Fig. 12 ▸).

We found that the weaver hesitated to adopt approach A in the loom set up for the integrated method. The weaver explained about other factors taken into consideration as follows. To remake fabric 14 in this loom setting, the weaver could have employed approach A (translation), but she preferred^2^ not to because it would be more laborious and time consuming than using approach B (vertical reflection). To remake the same pattern by different approaches, different quantities of shafts are calculated. The more lines in a motif, the greater the number of shafts and their weight. A shaft is a set of two horizontal bars, made from bamboo rods, by which heddles are tied on (Fig. 6 ▸), to control sets of warp threads for raising or lowering without hand manipulation. In fabric 14, the motif was designed for 50 lines along the warp. If weaving through approach A, 50 shafts equivalent to 100 bamboo rods are required (50 × 2). This is not convenient and it is slower for the weavers to control the loom with their hands and feet on heavier equipment. This can also obstruct the sheds from opening wide enough for passing weft threads through. If weaving through approach B, only 25 shafts equivalent to 50 bamboo rods are required [(50/2) × 2]. Although the ‘tiny content’ was modified, the overall fabric resembles the vintage fabric. In fact, this tiny content appears as a minor difference from the vintage fabric, and most people do not usually notice it. We found that fabric 14 demonstrates a design alteration that goes along with the technical constraints in the integrated method.

## 4. Conclusion

In this article, we examined *sin tin chok*, a type of traditional skirt from northern Thailand – specifically *tin chok* fabrics from the Long district, Phrae province (Section 1.1). This study was undertaken because there were developments in part of the traditional *chok* weaving process. In this article, such process development for *chok* weaving is called ‘the integrated method’. We asked: how can we distinguish *tin chok* fabrics made by these two methods? The weaving master of the Long district advised us to compare the symmetry axes of the patterns made by the two methods (Section 1.2). Seventeen indigenous/vintage fabrics from Komol Antique Textile Museum were examined (Section 2.1). The main research methods included visual analysis (Section 2.2) and symmetry analysis (Section 2.3), while literature review was conducted intermittently in parallel. Visual analysis involved fieldwork, discussions, reviewing artefacts, taking photographs, classifying the ranges of symmetries into two series (the traditional version and the integrated version) and creating the pattern booklet. A method for altering the traditional patterns to the integrated patterns is explained in Section 2.2.1. Creating the pattern booklet is a creative process that can foster information exchange, learning and understanding about symmetries. It was also used for the pattern inspections where we consulted experts in symmetry analysis and discussed the relationships between the symmetries and the weaving methods of the Long textiles. Consequently, we identified five main findings as follows.

First, in Section 3.1, we described the textile setting in a loom for weaving by the integrated method, and the two techniques for handling the weft threads (a faster weave and a slower weave). The placement of a *tin chok* fabric when it is woven on the loom is different from when it is sewn to the skirt. With a deep understanding of these factors, one can correctly identify the direction in which the pattern progresses and draw vertical or horizontal axes of symmetry, hence the validity of symmetry classifications. Second, in Section 3.2, we affirmed that the hem, which is one of the four parts (supplementary part one, main part, supplementary part two and hem) of the detachable *tin chok*, is key to distinguishing the fabrics made by the two methods. The four parts of *tin chok* made by the integrated method will always have a common vertical axis of symmetry, whereas the vertical axes of symmetry of the hem of a fabric made by the traditional method may not be aligned with the motifs in the other three parts. Third, in Section 3.3, we presented the decoding of the symmetry analysis of the 17 traditional skirts (Series 1) and the corresponding patterns generated for weaving by the integrated method (Series 2), and explained the relationships between the symmetries generated for the two weaving methods. Fourth, in Section 3.4, we presented symmetry classifications identified from every pattern, and found the two symmetry approaches (translation and vertical reflection) by which fabric 14 can be woven by the integrated method. Fifth, in Section 3.5, we revealed the preference of the weaver to remake fabric 14 by the vertical reflection approach over the translation approach, mainly because it is faster, and this results in a fabric that has small details that are slightly different from the vintage fabric.

## Supplementary Material

Paper, with example of graph paper. DOI: 10.1107/S1600576722011153/in5067sup1.pdf

Book (partial), sin tin chok by NS 2012. DOI: 10.1107/S1600576722011153/in5067sup2.pdf

## Footnotes

1 A change of colour requires a change of weft threads, for which the weavers may swap a shuttle or use their little fingers or a weaving needle to pull the short length of threads through the warp threads to create the pattern elements.

2 This does not mean that the weaver will always avoid approach A (translation). It depends on the design of a pattern and its elements, the number of shafts needed, and a reasonable wage. In 2022, the weaver employed approach A to remake a new pattern design where its elements were based on traditional patterns, which are familiar to the weaver. This design was plotted on graph paper comprising 28 lines, corresponding to 56 bamboo rods, and so is still convenient for the weaver.
