# Supplementary material for: Design research with the use of visual and symmetry analysis in indigenous woven textiles
Source: J Appl Crystallogr. 2023 Feb 1;56(Pt 1):81–94. doi: 10.1107/S1600576722011153 (PMC9901921; doi:10.1107/S1600576722011153)
Supplement: Supplementary file 1 [file j-56-00081-sup1.pdf]

CO-CREATION THROUGH DESIGN PROCESS FOR REVIVING INDIGENOUS KNOWLEDGE ABOUT TRADITIONAL TEXTILE PATTERNS

Disaya Chudasri

The College of Arts, Media and Technology – Chiang Mai University, Thailand
Chiang Mai University, 239 Suthep road, Muang district, Chiang Mai 50200, Thailand
Email: disaya.c@cmu.ac.th
Mobile: +66 (0)89-116-1155 Office: +66 (0)53-920-299

Abstract

Woven textiles of Thailand are especially evident for their intangible cultural heritage, which contributes to the Soft Power of Thailand through various means, i.e. the national identity and branding. However, in the recent decades, a number of weaving communities have been declining as a sequence of the expansion of industrialization and globalization for trade of mass produced goods. Few young people are being trained in traditional weaving, whereas experienced weavers are over forty years of age. Indigenous knowledge of traditional weaving is likely to be lost in the near future, if there is no immediate action happened to reinforce their cultural adherence and continuity. Designers are encouraged to design for cultural revitalization. The author utilises research and educational activities to letting the young generation engaged in design for cultural revitalization. A co-creation project was implemented with the weaving communities of Long district, Phrae province because of the erosion of indigenous knowledge about woven textiles. These communities wanted to reinforce and revival their indigenous knowledge of woven textiles by developing some tools, which they could use to exchange knowledge with other people. They preferred a booklet of traditional textile patterns and a card game. It was agreed to explore a vintage collection of a type of traditional skirts, known locally as ‘sin tin chok’, which are exhibited in the ‘Komol Phaboraan Museum’. This design process lasted eight months, involving fieldwork and data collection, booklet design and design of a card game. The research group included the principal researcher, a specialist in game design and five undergraduate students. This design process embraced people within the district (i.e. textile masters, experienced weavers, villagers) and outside the district/province (i.e. undergraduate students, school children, teachers, potential users and tourists). Co-creation through design process can enable knowledge exchange between engaging actors that does not limit to indigenous knowledge about woven textiles. Their knowledge exchange may expand to other knowledge domains, such as local history and languages, and digital technology.

Keywords: *Co-creation, Design process, Design and culture, Traditional textile patterns, Weaving Communities*

Biography

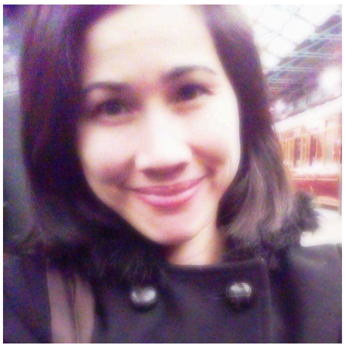

Disaya Chudasri has been teaching in the College of Arts, Media and Technology at Chiang Mai University, Thailand since 2009. She gained the Ph.D. in Design from Lancaster University, UK in 2015. Previously, Disaya worked for more than ten years as a senior designer in design companies and dealt with commercial projects that involved graphic design, packaging design, corporate identity and branding. Disaya is keen to contribute experiential knowledge to enable people in educational, enterprising and research settings. Her research interests include design in relation to sustainability, art, craft and culture, business and marketing, knowledge management and education.

See also: www.designineducation.com

CO-CREATION THROUGH DESIGN PROCESS FOR REVIVING INDIGENOUS KNOWLEDGE ABOUT TRADITIONAL TEXTILE PATTERNS

Disaya Chudasri

The College of Arts, Media and Technology – Chiang Mai University, Thailand
Chiang Mai University, 239 Suthep road, Muang district, Chiang Mai 50200, Thailand
Email: disaya.c@cmu.ac.th
Mobile: +66 (0)89-116-1155 Office: +66 (0)53-920-299

Abstract

Woven textiles of Thailand are especially evident for their intangible cultural heritage, which contributes to the Soft Power of Thailand through various means, i.e. the national identity and branding. However, in the recent decades, a number of weaving communities have been declining as a sequence of the expansion of industrialization and globalization for trade of mass produced goods. Few young people are being trained in traditional weaving, whereas experienced weavers are over forty years of age. Indigenous knowledge of traditional weaving is likely to be lost in the near future, if there is no immediate action happened to reinforce their cultural adherence and continuity. Designers are encouraged to design for cultural revitalization. The author utilises research and educational activities to letting the young generation engaged in design for cultural revitalization. A co-creation project was implemented with the weaving communities of Long district, Phrae province because of the erosion of indigenous knowledge about woven textiles. These communities wanted to reinforce and revival their indigenous knowledge of woven textiles by developing some tools, which they could use to exchange knowledge with other people. They preferred a booklet of traditional textile patterns and a card game. It was agreed to explore a vintage collection of a type of traditional skirts, known locally as ‘sin tin chok’, which are exhibited in the ‘Komol Phaboraan Museum’. This design process lasted eight months, involving fieldwork and data collection, booklet design and design of a card game. The research group included the principal researcher, a specialist in game design and five undergraduate students. This design process embraced people within the district (i.e. textile masters, experienced weavers, villagers) and outside the district/province (i.e. undergraduate students, school children, teachers, potential users and tourists). Co-creation through design process can enable knowledge exchange between engaging actors that does not limit to indigenous knowledge about woven textiles. Their knowledge exchange may expand to other knowledge domains, such as local history and languages, and digital technology.

Keywords: *Co-creation, Design process, Design and culture, Traditional textile patterns, Weaving Communities*

TRADITIONAL TEXTILE, DESIGN, RESEARCH AND EDUCATION

In the context of traditional textile production, within a given cultural group, there will be a preferred symmetry or symmetries used to decorate objects (Brainerd cited in Arnold, 1992, p.8). Their group will consistently use the specific symmetries in their design systems (Washburn and Crowe cited in Arnold, 1992, p.55). The design of pattern structure from any given cultural group shows their unique preferences for a cultural system in many aspects (Washburn and Crowe cited in Arnold, 1992, p.55), for example, cultural and historical principles, geometric principles in practice and correlation to design (Washburn and Crowe, 1988, p.ix; Arnold, 1992, p.2). The fundamentals of pattern structure and their symmetry characteristics can be used as a tool for cultural analysis (Arnold, 1992, p.55; Hann, 1992, pp.581, 589; Washburn and Crowe, 1988, p.ix; Hann, 2003, p.81).

Woven textiles of Thailand are especially evident for their intangible cultural heritage (Conway, 1992, p.9; Ministry of Culture, 2009), which contributes to the Soft Power of Thailand through various means, i.e. the national identity and branding (verbal communication with Kosit Panpiemras in 2012). Soft Power is a means for a country to success in world politics, in ways that a country is able to persuade others to do what it wants without force or coercion. It describes and evaluates the comprehensive national strength, including culture, political values and foreign policies (Nye, 2004). Woven textiles are an integral part of the social life and religious belief associated with Buddhism of Thai people from their birth to death (Conway, 1992, p.9). For example, woven textiles are used for cloth-making, household products, gifts-giving, and presenting in religious ceremonies (Conway, 1992, pp.41, 135). Additionally, woven textiles play a role related to financial value (Museum of the Bank of Thailand Northern Region Office, 2011).

However, in the recent decades, a number of weaving communities have been declining as a sequence of the expansion of industrialization and globalization for trade of mass produced goods (Warren, 1983; Wherry, 2008). A number of the younger generations have migrated from villages to urban areas for job opportunities, which can provide a lucrative income, and a modern lifestyle. Few young people are being trained in traditional weaving, whereas experienced weavers are over forty years of age (Chudasri, 2015). Indigenous knowledge of traditional weaving is likely to be lost in the near future, if there is no immediate action happened to reinforce their cultural adherence and continuity (Chudasri, 2015). It is critical to enable our understanding of design in relation to cultural significance, for example, design structure can be used as an indicator of cultural adherence, continuity and change (Arnold, 1992, p.55).

Designers are encouraged to make positive contributions of design for cultural revitalization at the local level (Walker, Evans, Cassidy, Jung, and Holroyd, 2018). Enabling factors, which support and underpin how design can reconnect with various aspects of traditional culture are identified, including promotion, enterprise, and research and education (Evans, Holroyd, Walker, Cassidy and Jung, 2018, p.344). Scholars address that 'the focus of higher education on art, craft, and design is facing constant changes', which focus more on design, digitalization and an industrial approach, for example in universities in UK and Finland, rather than cultural and traditional aspects that strengthen the sense of local identity (Kokko, 2018, pp.231–232). The author is an educator, researcher and designer, working in a university in northern Thailand. I admit that this situation is also happening in higher education institutions throughout Thailand, where several courses are being replaced by new courses involving computer programs and digital technology.

The author inquired *how research and education could be used as a platform to connect craft enterprise and multiple actors to support the revival of indigenous knowledge about traditional textile patterns?* Co-creation design is a strategic approach to innovation;

it is the key to investigate new opportunities for enterprises and unlocking new sources of competitive advantage (Frow, Nenonen, Payne and Storbacka, 2015, p.463). However, there is deficiency of the literature that 'offers a detailed exploration of the specific dimensions and categories that are important in co-creation design' (Frow, Nenonen, Payne and Storbacka, 2015, p.464). For example, how firms can purposefully identify co-creation opportunities, what tools and processes can enable effective cocreation (Frow, Nenonen, Payne and Storbacka, 2015, pp.464, 356), how we can encourage work on practices for collaborating with partners? (Barczak, 2012 cited in Frow, Nenonen, Payne and Storbacka, 2015, p.464).

RESEARCH CONTEXT, APPROACHES AND METHODS

Research approaches included ethnography, use of archives and museums, geometric symmetry concepts, and grounded theory (based on Cassidy, 2018, pp.277–289).

Selection of the case study

Most of the population in northern Thailand are the 'Tai Yuan', an ethnic Tai group that settled in this region for over many centuries (Conway, 1992, p.135; Museum of the Bank of Thailand Northern Region Office, 2011). The Tai Yuan women are specialized in a weaving technique known in a local word as 'chok' (McIntosh, 2012, pp.3–9), which means to weave and create patterns by slipping weft threads of different colours in and out on the loom (Suchitta, 1989, p.97). This weaving technique is described in English as discontinuous supplementary weft (McIntosh, 2012, p.6). They usually produce the special type of traditional skirts, which is called in a local term as 'sin tin chok' (Figure 1A). It is made up of three separately woven bands sewn together, including a waistband, a body part and a lower part (Figure 1B) (Suchitta, 1989, p.97; McIntosh, 2012, pp.3–6). Chok weaving technique is employed for making the decorative patterns at the lower part of these skirts (Figure 1C). These patterns can be used to distinguish woven textiles of the Tai Yuan living in one area from others, such as those residing in the northern region from the central provinces (McIntosh, 2012, pp.3, 5). This type of skirts can also distinguish the Tai Yuan ethic group from the other Tai groups of Thailand, such as Tai Lue, Tai Khoen, Tai Yai, Tai Mao, and Tai Phuan (McIntosh, 2012, pp.3, 5).

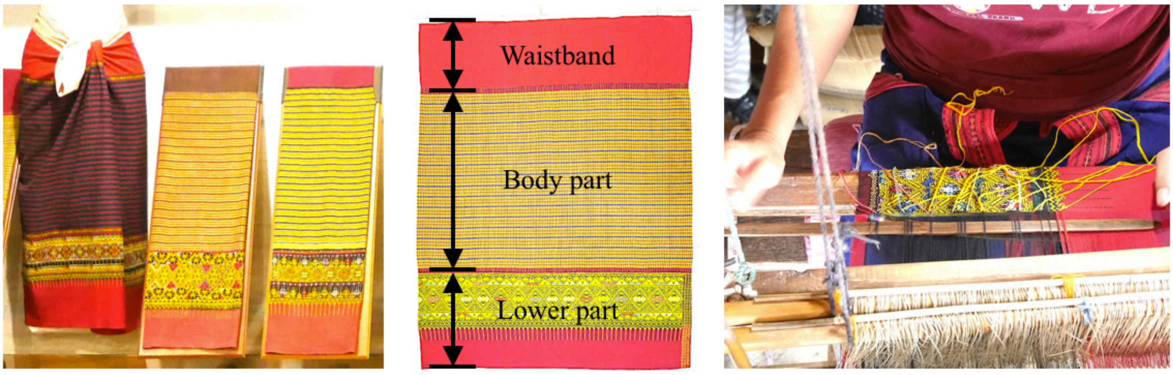

A B C

Figure 1. The Special Type of Traditional Skirts (i.e. Sin Tin Chok) and 'Chok' Weaving (Photograph by Chudasri, 2018)

The identification of a critical area for design

In 2018, an exploratory research was implemented with a case study of the weaving communities from Long district, Phrae province. In northern Thailand, they are one of the best-known producers of a type of traditional skirts that is called locally as 'sin tin chok'. In the previous study, Chudasri (2015, pp.174–186, 202–207; 2018, pp.88–91) identified four

potential areas for design to foster the future viability of weaving communities. These four areas include: (i) design and production development; (ii) product design and development; (iii) design in relation to marketing and sales; (iv) and design to support weaving expertise transfer between generations. Based on this finding, the group leaders and experienced weavers in Long district were inquired in 2017 and 2018 to select a critical area for design intervention. They addressed a critical need for design intervention that can cure the erosion of indigenous knowledge about traditional textile patterns. Additionally, based on a list of the potential mechanisms, which can enable Weaving Expertise Transfer (i.e. books, weaving courses and training materials, promotional events, learning centers and digital media), they identified a pattern booklet and a card game for which they could use to enable knowledge exchange.

The research objectives and co-creation opportunities

Therefore, this design research was initiated with three objectives as follows. First, it was **to create the patterns booklet**, which displays textile patterns in photographs and plotted graphs, and provides information about the history of Long weaving, the identity of Long woven textiles, the patterns' descriptors and meaning. Second, it was **to create the card game**, which is applicable to various groups of people. The Long representatives preferred products in the form of physical objects, and not yet on digital gadgets. From their perspective, physical objects are easy to use and can be applicable to various groups of people in various situating environments. For example, weavers may use the pattern booklet to communicate with training novices, visitors or potential buyers. Tourists may play a card game, while they are waiting for entering the museum. Potential users of these products may vary from weavers, weaving trainees, villagers, school children, tourists, visitors, textile enterprises and interested people from within the district and beyond. These requirements were linked with the third objective, which was **to generate co-creation opportunities through the design process**. This paper focuses on *co-creation through the design process*. Other aspects of this research project will be written separately in other papers.

Sampling materials

The seventeen traditional skirts exhibited in the 'Komol Phaboraan Museum' in Long district (Figure 2: Left) were the sampling materials for this research. These seventeen skirts used to belong to the Tai Yuan residing in Long district, who donated their skirts to this museum. Each of these skirts are estimated to be more than a hundred years old (from verbal discussion with Komol, the owner of this museum). Komol selected these seventeenth skirts for display based on their patterns, which uniquely represent the local identity and are different from the others. Geometric symmetry concepts were introduced for the analysis of decorative patterns at the lower part of these skirts. An example of the decorative patterns is shown in Figure 2: Right.

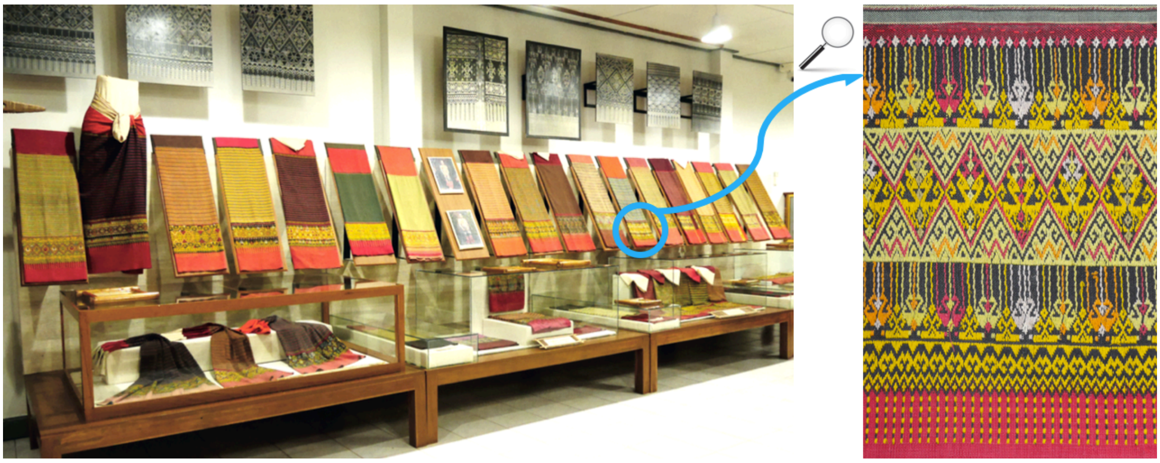

Figure 2. A Collection of the Traditional Skirts Exhibited in the 'Komol Phaboraan Museum' (Photograph by Author, 2018)

Research process

This research employed 'grounded theory approach' for collecting information and the analysis of information collected (Glaser and Strauss 1967 cited in Castree, Kitchin, and Rogers 2013; Scott 2014).

> *'Grounded theory is a research method that generates theories inductively on social processes through the analysis of qualitative data' (Glaser and Strauss, 1967 cited in Cassidy, 2018, p.283). …'Grounded theory approach is normally carried out by researchers who are already experienced in the area being studied. It allows them to probe deeply into the data collected' (Cassidy, 2018, p.284).*

The author conducted the fieldwork without any preconceived design framework or theoretical statements, but with experiential knowledge about relation between geometric symmetry in the practice of textile design, digital technology, co-creation design, task management, and research process. This research process consisted of three main tasks (Figure 3): creating the patterns booklet (Task-1) and the card game (Task-2), and generating co-creation opportunities through the design process (Task-3). Task-1 and Task-2 comprised several steps, some of which are carried out iteratively in respond to their other relevant steps. For example, an iterative process between the booklet design and developments of it, or between the gameplay design and the game testing. Task-3 was carried out when co-creation was feasible and appropriate to activities of Task-1 and Task-2. For example, co-creation within the research group through design process, co-creation between the research group and the community representatives during the product presentations and discussions, and co-creation between the research group with the potential users during the game testing. Fieldwork was mainly conducted in Long district three times, every three months. Each visit in Long district lasted two days. Fieldwork was also conducted beyond Long district.

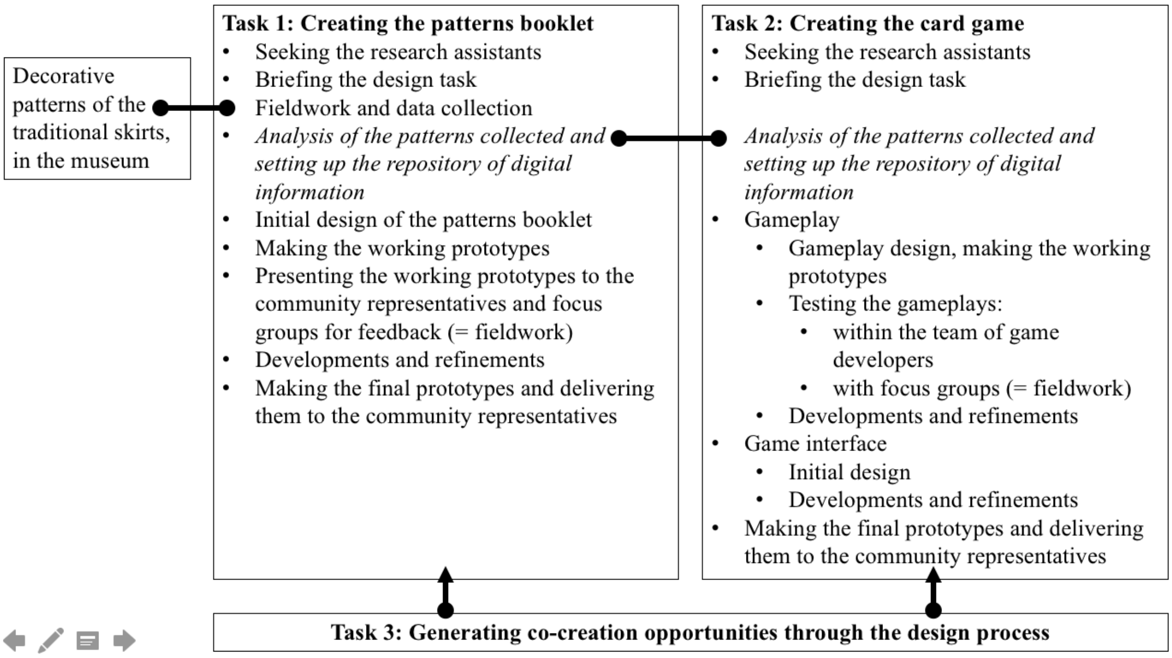

Figure 3. The Research Process

The research participants

Multiple actors were involved in this research. The research group from the education institution included the author (acting as the principal researcher, the project manager, the design director), a specialist in game design and five undergraduate students. Upon the announcement of a need for research assistants that offers a little amount of remuneration for their services, a student came in contact and span to the group of five. Other engaging actors also contributed to this research project. They can be classified into two groups. First, the representatives from the weaving communities of Long district included two weaving experts (age 64, 67) and two weavers (age 44, 48). Three of them have continuous experience in woven textiles for more than 30 years with cultural purposes and commercial purposes. Although, another weaver (age 48) has just returned to weaving for about four years, she was a fast learner since she grew up in Long district and used to be involved in weaving when she was young. Four of them were the key informants pertinent to the indigenous knowledge of woven textiles.

Second, the people, who participated in the game testing. It was conducted in Long district with local people and tourists from other provinces. The local people included an artist/writer (age 44), and three children (age 6-7) and five children (age 13-15) accompanied by their parents or guardians. The tourists included two university teachers (age 37, 39) and a group of eight tourists (age 35-48) visiting the Komol Phaboraan Museum. Additionally, the game testing was conducted in two provinces in the northern region. The participants included four undergraduate students (age between 20-22) and five children (age 11-12). Prior to conducting the game testing, the research group got permission from the key informants and the participants, including the children's parents or guardians.

Methods and tools for collecting information

Methods included interviews (and audio recording if permitted), review of artifacts, photographing, gameplay testing, observations, and video and audio recording (if permitted). Tools included digital cameras, audio recorders, the working prototypes of the patterns booklet and the card game in softcopy and hardcopy versions, a computer system connecting to the Internet, Cloud Storage and software (i.e. Microsoft Excel, Microsoft Word, Photoshop, Illustrator and Indesign).

FINDINGS AND DISCUSSION

During this project implementation, the research process was developed into more details, which were adjusted at times to suit with the available resources and the situations. Detailed exploration of the design process that incorporated co-creation are depicted as follows.

Co-creation through design process of the Patterns Booklet (Task-1)

This design process consisted of two phases: exploration (Figure 4); and finalization (Figure 5). The purpose of the exploration phase was to decode the textile patterns from digital photographs and record this information in a computer system. It was also to explore the competency and understanding of the research assistants, budget and production time, and opportunities for co-creation within the research group as well as with the community representatives; this was also applicable to design process of the card game. The initial design of the patterns booklet was the result of this exploration phase. This booklet included the eleven decorative patterns. The community representatives were satisfied with this working prototype and provided recommendations as follows. The research group should: complete the seventeen decorative patterns; depict the decoded patterns with their change as a result of production developments; revise the design layout, and add name and description of every pattern motif. Therefore, the author sought a more budget in order to fulfil these recommendations in the finalization phase. It is noted that team meeting and working with close supervision between the principal researcher and the research assistants was arranged more than it was planned, in order to ensure that the assignments were fulfilled and met the standard within the timeframe given.

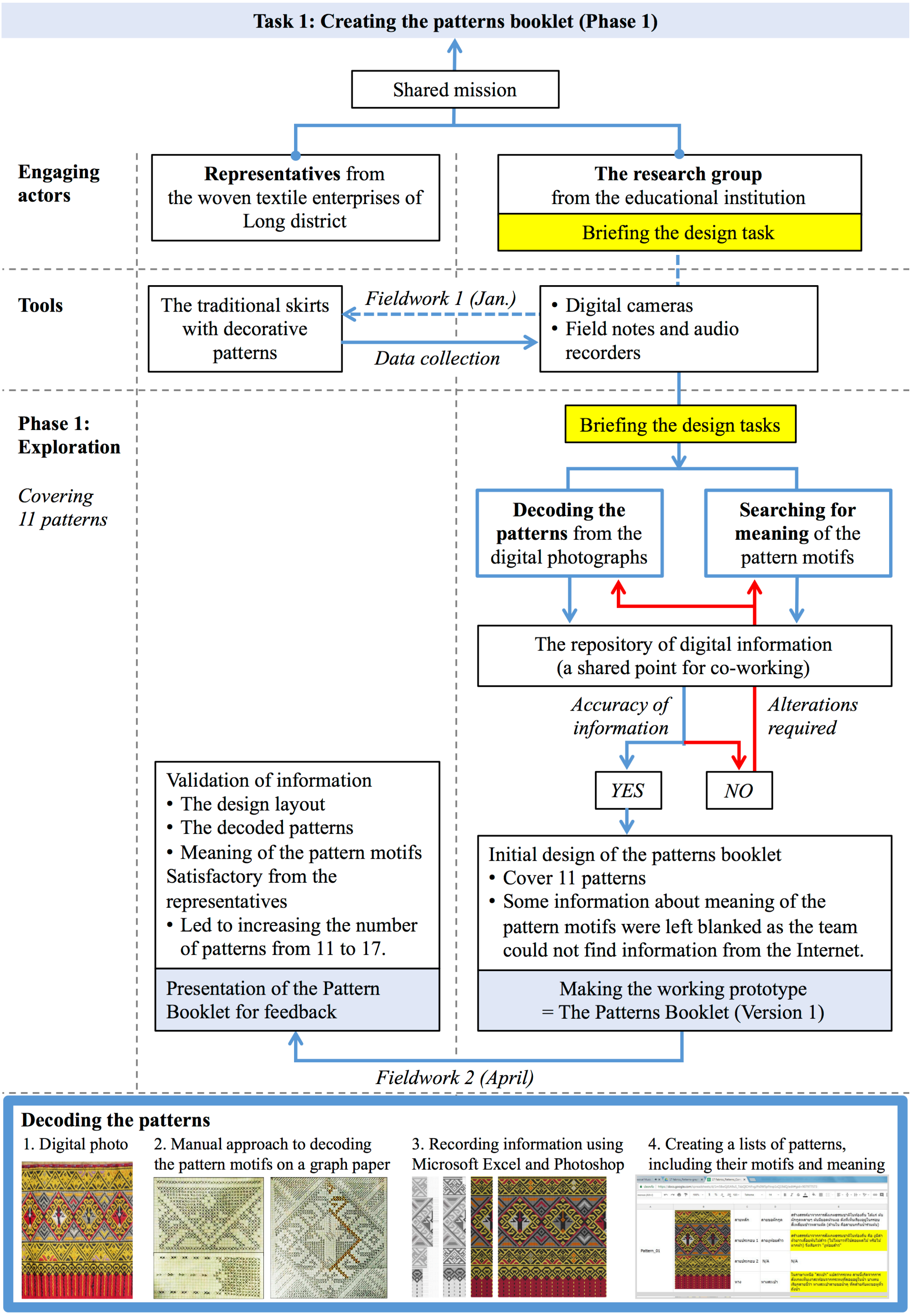

Figure 4. Co-creation through Design Process of the Patterns Booklet – Phase 1

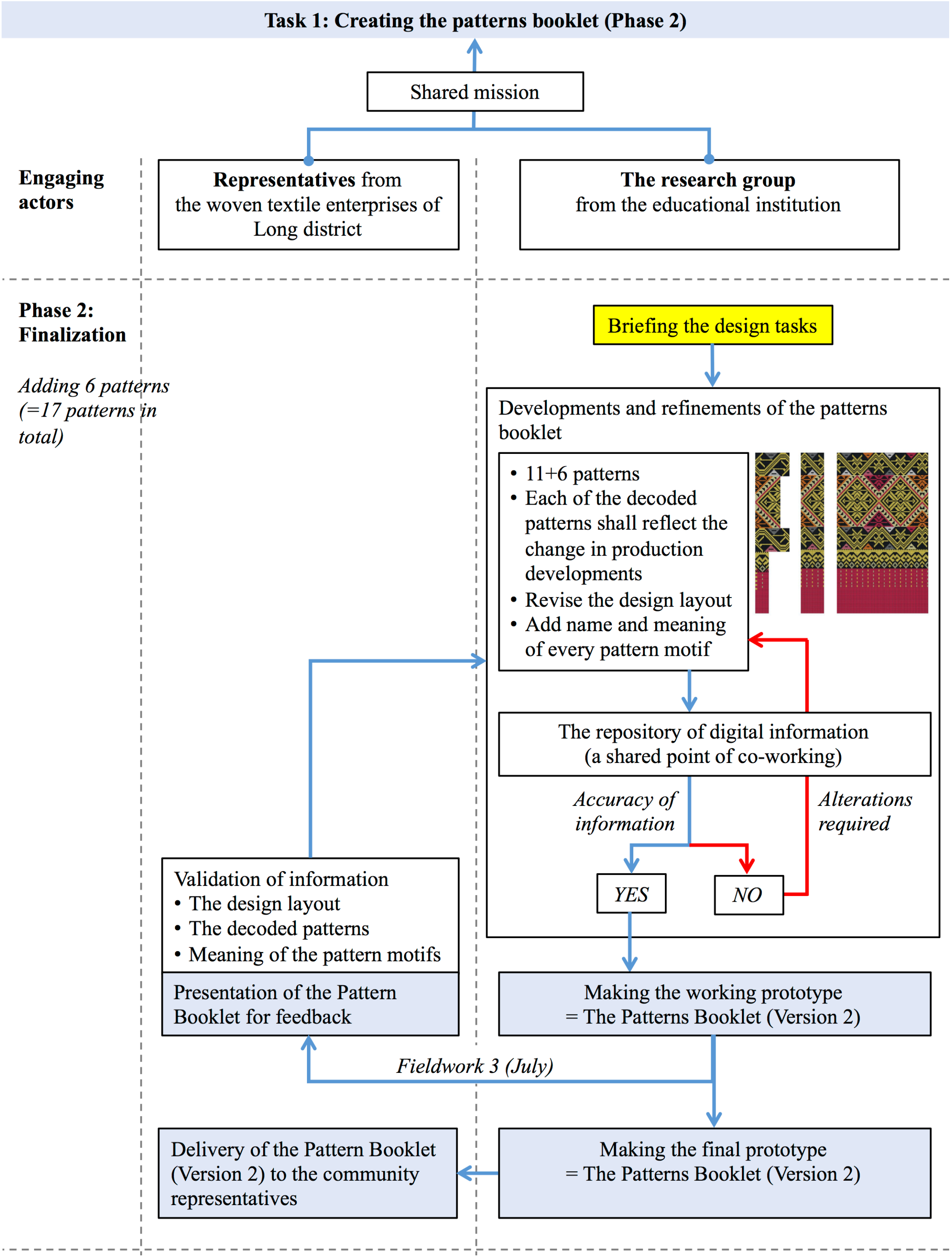

Figure 5. Co-creation through Design Process of the Patterns Booklet – Phase 2

Co-creation through design process of the card game (Task-2)

This design process consisted of two phases: exploration (Figure 6); and finalization (Figure 7). Design processes of the patterns booklet and the card game were linked through textile patterns and fieldwork 2 and 3. The purpose of this exploration phase was to explore a game, which could interest the community representative and potential users. In the

beginning, the research group came up with different ideas for developing the card game, such as matching textile patterns or creating the full patterns in ways which players could also learn about the design structure of traditional patterns. Yet, the research group agreed on developing a board game with a storyline about collecting woven fabrics or trading of. The research group developed the gameplay and conducted game testing within the research group several times prior to making a proper prototype of cards and a board. Next, this board game was tested with potential users from Long district (including one artist/writer, two weavers) and from outside this district (including two university teachers). This board game was failed to be accepted because it had so many rules, which were considered ‘complicated’, ‘taking sometimes to understand’. As a result, they were not in the mood to play this game. They recommended to develop the card game without a board game, which are easy to play. One set of cards should be applicable to playing different games. In Phase 2, the research group designed the card game for multiple players and developed two gameplays entitled: Connecting Pattern Motifs; and Matching Pattern Motifs. The same set of 68 cards (17 patterns x 4 parts = 68 cards) could be used in both gameplays. Game testing was part of the gameplay developments, which the research group carried out several times with the Long representatives and potential users. Eventually, they accepted these two gameplays and it was observed that they felt fun in playing these games.

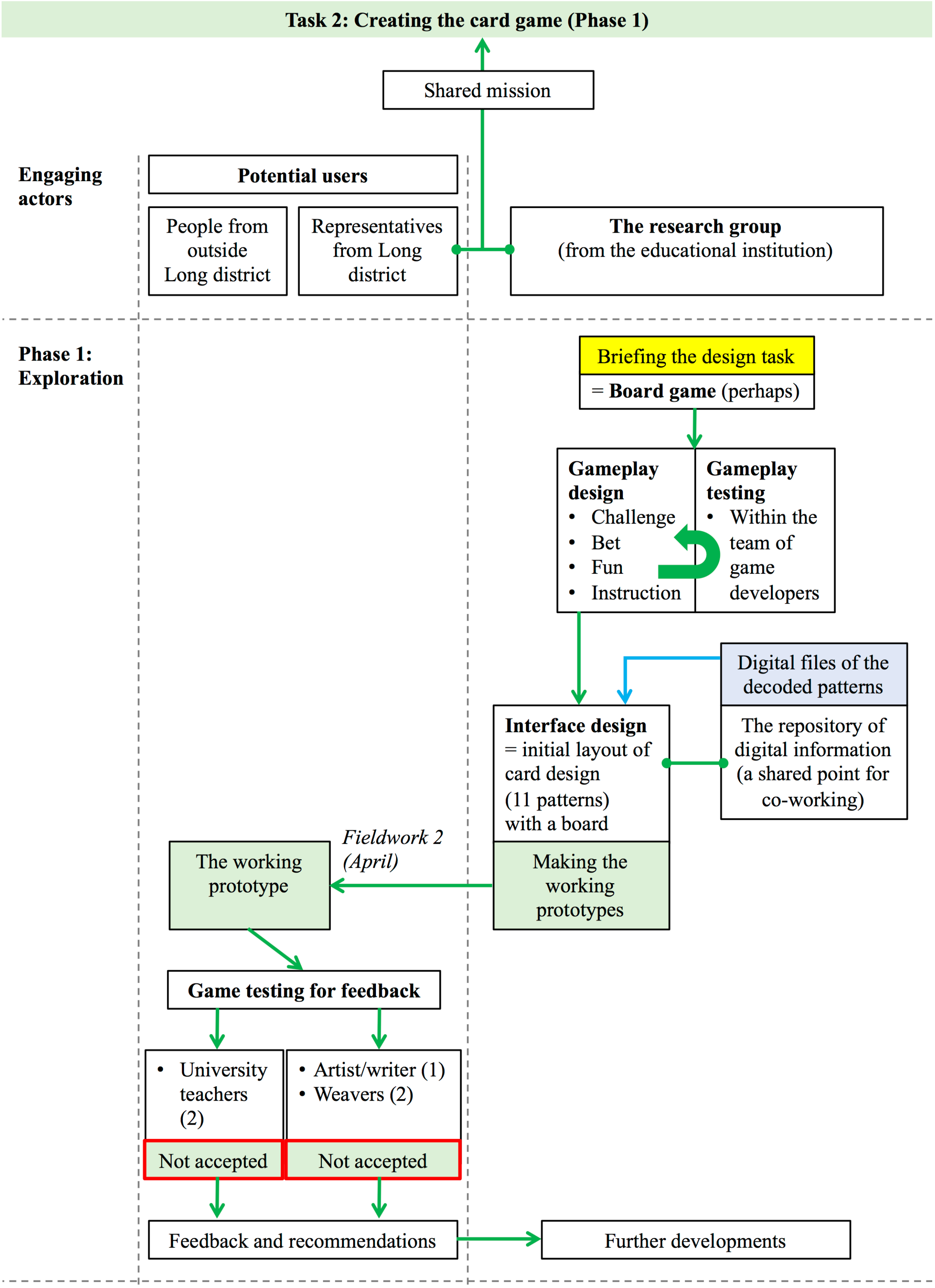

Figure 6. Co-creation through Design Process of the Card Game – Phase 1

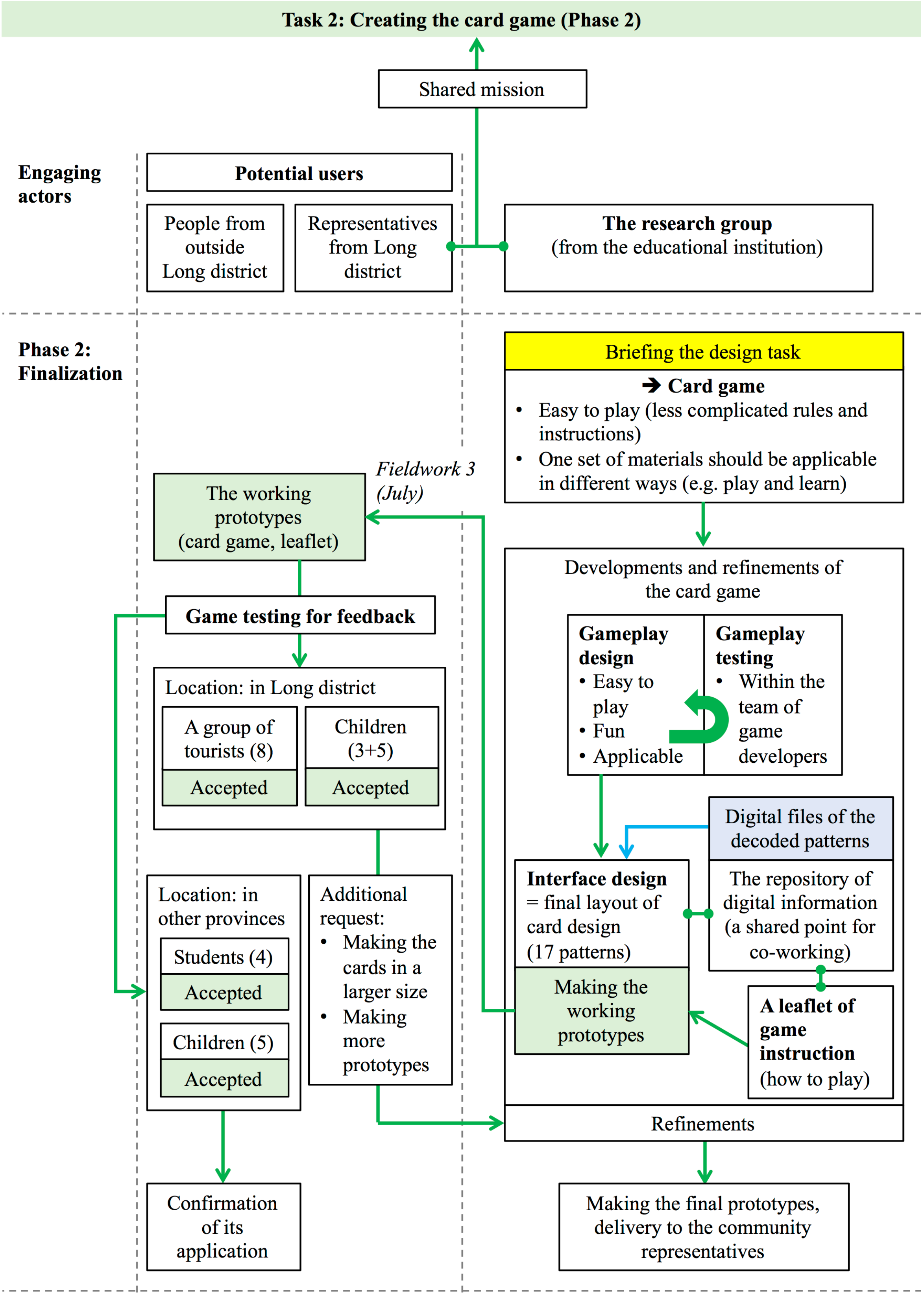

Figure 7. Co-creation through Design Process of the Card Game – Phase 2

CONCLUSION AND IMPLICATIONS

This paper begins with the significance of traditional textile, design, research and education with an emphasis on a critical need to revive traditional weaving and woven textiles in Thailand. The author inquired *how research and education could be used as a platform to connect craft enterprise and multiple actors to support the revival of indigenous knowledge about traditional textile patterns?* Co-creation design was employed as a strategic approach to investigate new opportunities for enterprises and increase their competitive advantage. However, there is gap in the existing literature, for example, those: offer a detailed exploration of the specific dimensions and categories that are important in co-creation design; demonstrate how firms can purposefully identify co-creation opportunities; what tools and processes can enable effective cocreation. In 2018, a research project was implemented in association with the weaving communities of Long district. The objectives were to create the patterns booklet and the card game, and to generate co-creation opportunities through the design process. This paper depicts detailed exploration of co-creation through design process of the Patterns Booklet (Task-1) and the card game (Task-2). Co-creation in these design processes occurred in various dimensions, including: co-creation for access to resources such as licensed software offered to the university members; co-creation in the forms of ideas, design and production; co-creation through various engagement platforms such as digital applications, tools and products, museum space (based on Frow, Nenonen, Payne and Storbacka, 2015, p.471). Co-creation also enables knowledge exchange between engaging actors, whose their knowledge expand to other knowledge domains, e.g. local history and languages, and digital technology – besides woven textiles.

BIBLIOGRAPHY

Arnold, F. (1992) 'The Geometry of Regular Repeating Pattern'. In: *Textile Progress*, 22:1, pp.1-62.

Cassidy, T. (2018) 'Research Approaches for Culturally Significant Design'. In: *Design Roots: Culturally Significant Designs, Products, and Practices*. Bloomsbury Publishing, London, pp.277–289.

Chudasri, D. (2015) An investigation into the potential of design for sustainability in the handicrafts of northern Thailand. Ph.D. Thesis in Lancaster University, Lancaster, UK.

Conway, S. (1992) *Thai Textiles*. British Museum Press, London.

Evans, M., Holroyd, A. T., Walker, S., Cassidy, T. and Jung, J. (2018) 'Strategies for Revitalization of Culturally Significant Designs, Products, and Practices'. In: *Design Roots: Culturally Significant Designs, Products, and Practices*. Bloomsbury Publishing, London, pp.341–359.

Frow, P., Nenonen, S., Payne, A. and Storbacka, K. (2015) 'Managing Co-creation Design: A Strategic Approach to Innovation'. In: *British Academy of Management*, Vol. 26, pp. 463–483. John Wiley & Sons, Oxford.

Hann, M.A. (1992) 'Symmetry in Regular Repeating Patterns: Case Studies from Various Cultural Settings'. In: *The Journal of the Textile Institute*, 83: 4, pp.579-590.

Hann, M.A. (2003) 'The Fundamentals of Pattern Structure Part III: The Use of Symmetry Classification as an Analyical Tool'. In: *The Journal of the Textile Institute*, Vol. 94 Part 2, pp.81–88.

Kokko, S. (2018) 'The Role of Higher Education in Sustaining Culturally Significant Crafts in Estonia'. In: *Design Roots: Culturally Significant Designs, Products, and Practices*. Bloomsbury Publishing, London, pp.231–232.

McIntosh, S. D. (2012) “Tai Yuan textiles of Thailand”. In: *Textiles Asia Journal*, Hong Kong, Vol.4, No.2, pp.3-9.

Ministry of Culture. (2009) Seminar of progressing from the cultural capital to the creative economy, Bangkok. Available in Thai language.

Museum of the Bank of Thailand Northern Region Office. (2011) Magnificent Lanna, Glamorous Textiles. Chiang Mai.

Nye, J. (2004) *Soft Power: The Means to Success in World Politics*. New York: Public Affairs.

Suchitta, P. (1989) “Mental template: the case of the Tai Lao Pha Sin”. In: *Asian Folklore Studies*, Vol.48, pp.95-105.

Walker, S., Evans, M., Cassidy, T., Jung, J. and Holroyd, A. T. (2018) Design Roots: Culturally Significant Designs, Products, and Practices. Bloomsbury Publishing, London.

Warren, W. (1983) *Jim Thompson: The Legendary American of Thailand*, Jim Thompson Thai Silk Company, Bangkok.

Washburn, D. and Crowe, D. (1988) *Symmetries of Culture: Theory and Practice of Plane Pattern Analysis*. University of Washington Press, Seattle and London.

Wherry, F. (2008) *Global Markets and Local Crafts: Thailand and Costa Rica Compared*, Johns Hopkins University Press, Baltimore.
