# Supplementary material for: Design research with the use of visual and symmetry analysis in indigenous woven textiles
Source: J Appl Crystallogr. 2023 Feb 1;56(Pt 1):81–94. doi: 10.1107/S1600576722011153 (PMC9901921; doi:10.1107/S1600576722011153)
Supplement: Supplementary file 2 [file j-56-00081-sup2.pdf]

ซิ่นตีนจกแห่งลุ่มน้ำแม่ปิง

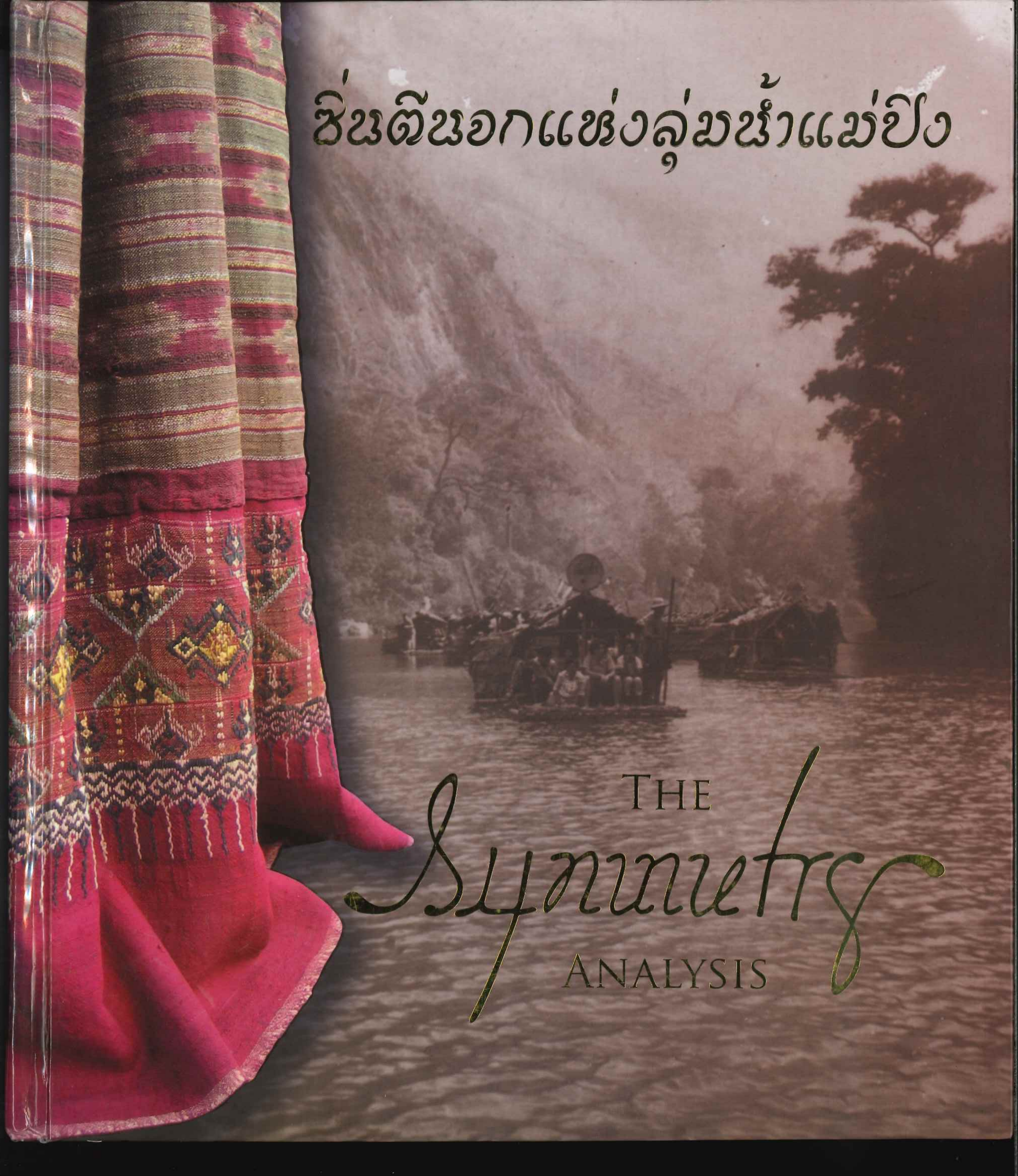

THE Symmetry ANALYSIS

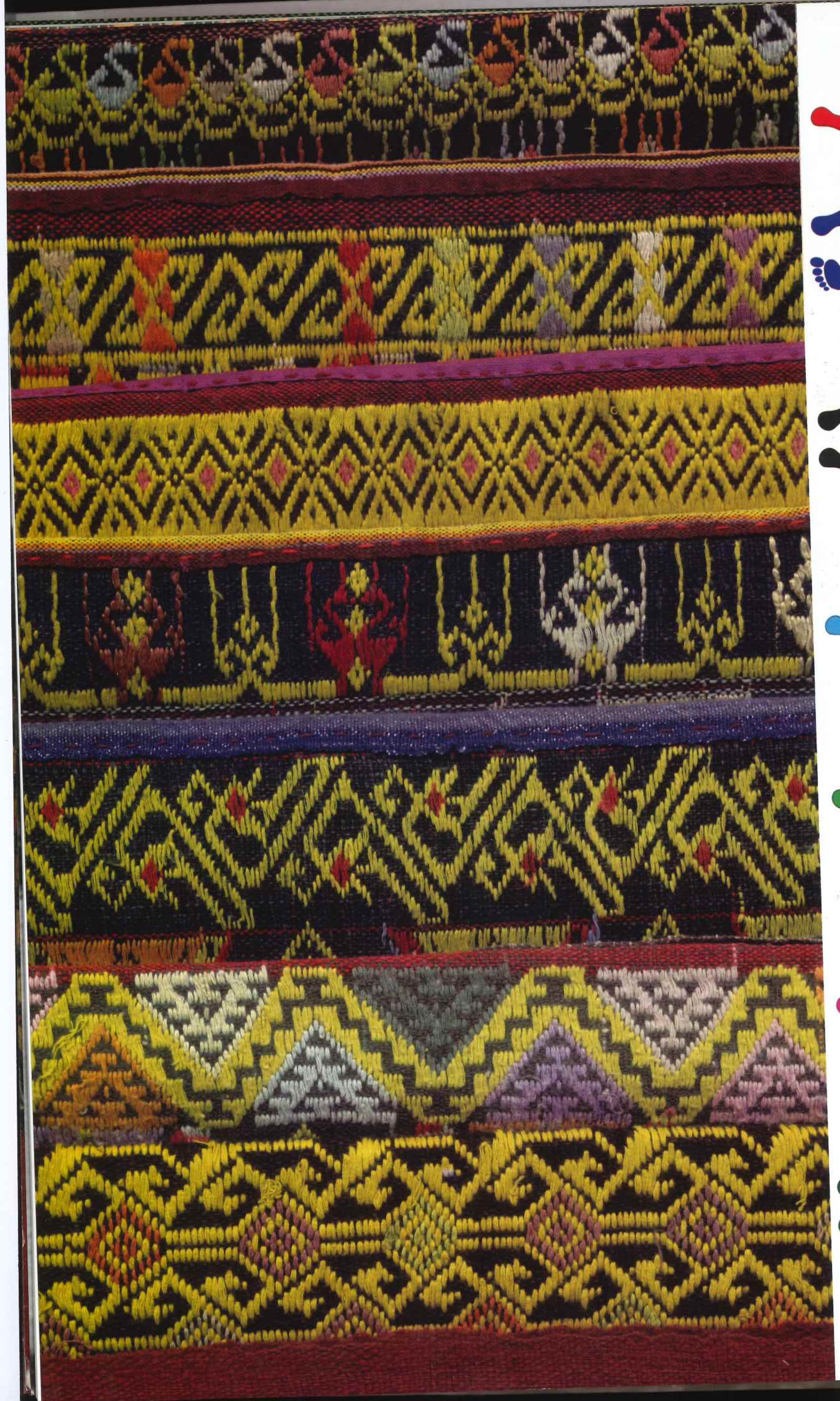

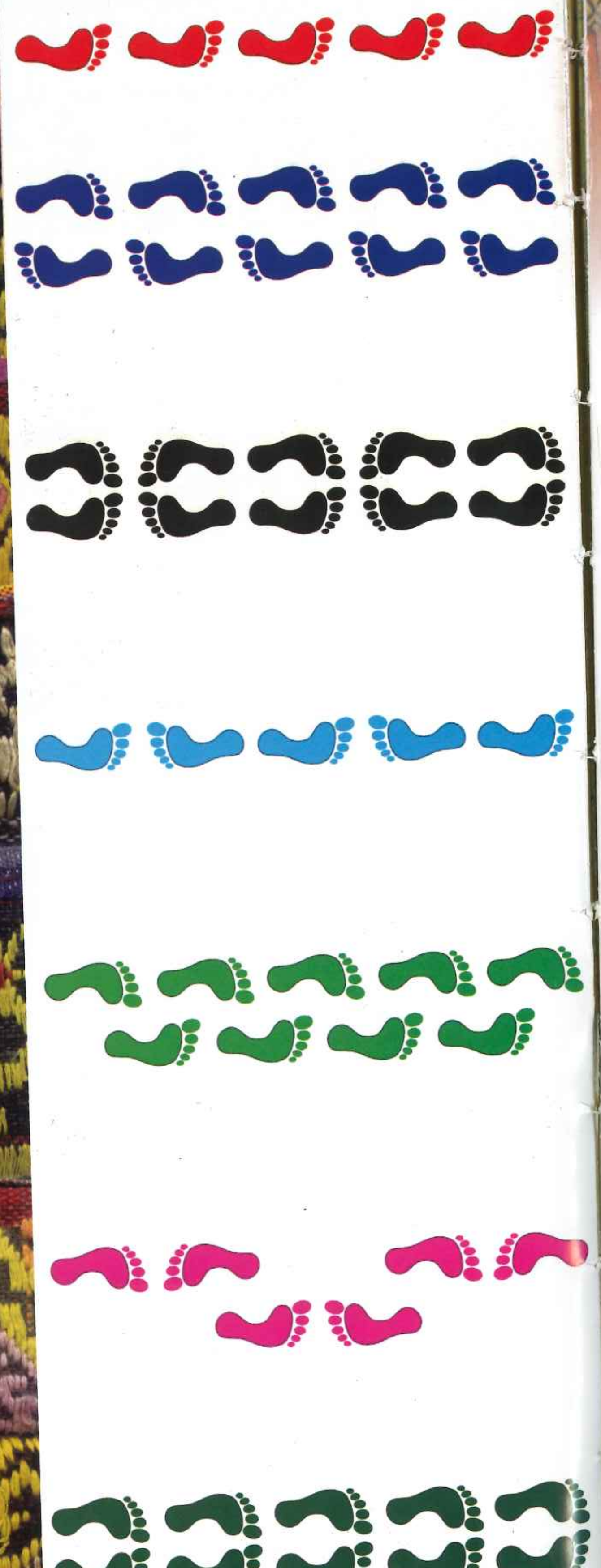

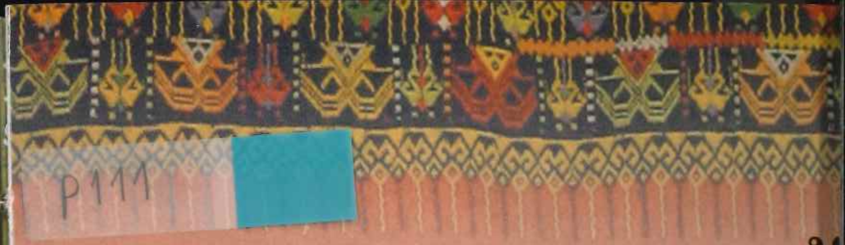

แบบสมมา

๑. การออกแบบลวดลาย (Pattern Designing)

การออกแบบหมายถึงการสร้างสรรค์ผลงานหนึ่งขึ้นมา โด
จัดระบบความคิดทางนิเวศวิทยา สังคมวิทยา มานุษยวิท
คณิตศาสตร์ผนวกเข้ากับความคิดทางศิลปะระหว่างกระบ
สร้างสรรค์นั้นเพื่อให้ผลงานที่ได้มีความสวยงาม และเห
การออกแบบลวดลายจึงเป็นการสร้างสรรค์ลวดลายบนสิ่งป
ขึ้นหนึ่งขึ้นมา โดยมีการจัดระบบความคิดทางศาสตร์ต่า
คณิตศาสตร์ผนวกเข้ากับความคิดทางศิลปะเพื่อให้สิ่งประดิ
นั้นมีความสวยงาม

การออกแบบลวดลายมีความสำคัญในการสร้างสรร
ประดิษฐ์ในหลายมุมมอง บางครั้งการออกแบบลวดลายเป็น
ปัญหาการมีบริเวณว่างบนงานประดิษฐ์ การออกแบบลวดลา
นอกจากสามารถเพิ่มคุณค่าของงานประดิษฐ์แล้ว ยังสื่อให้เ
ทางวัฒนธรรมประเพณีของงานประดิษฐ์และที่สำคัญก็คือ
แบบลวดลายสามารถสื่อให้เห็นแนวคิดที่หลอมรวมกันในงาน
ในด้าน นิเวศวิทยา สังคมวิทยา มานุษยวิทยา และ คณิ

ตัวใหญ่	ตัวเล็ก	คำอ่าน	ตัวให[illegible]
A	α	อัลฟา	I
B	β	เบตา	K
Γ	γ	แกมมา	Λ
Δ	δ	เดลตา	M
E	ε	เอปสิลอน	N
Z	ς	อิเซตา	Ξ
H	η	เอตา	O
Θ	θ	เธตา	Π

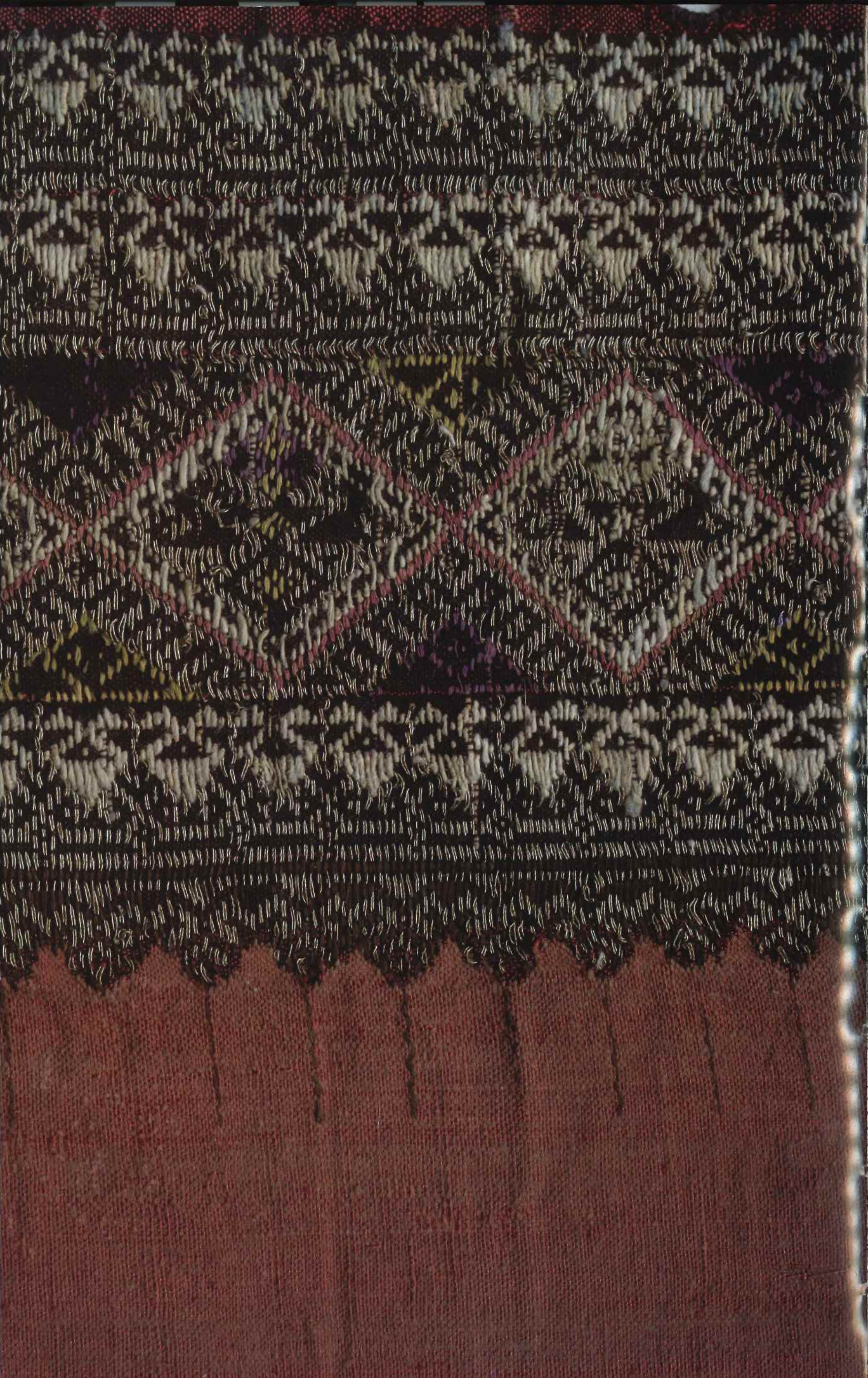

▲ ซิ่นตีนจกใช้ดิ้นเงิน ดิ้นทองและเส้นไหม ตัวซิ่นเป็นฝ้ายกุก (เส้นยืนพิเศษ) พบที่บ้านโฮ่ง ตำบลลี้ อำเภอลี้

บทที่ ๖
แบบสมมาตรของซิ่นตีนจกโหล่งลี้

เมืองลี้เป็นเมืองที่มีประวัติความเป็นมายาวนานตั้งแต่สมัยกรุงสุโขทัย ชื่อของเมืองลี้ได้ปรากฏในนามของ ลิ ลี้ และลิบ ในเอกสารเชิงประวัติศาสตร์หลาย ๆ ชิ้น พระราชพงศาวดารกรุงเก่าฉบับหลวงประเสริฐอักษรนิติ์ กล่าวว่า “ศักราช ๘๑๘ แต่งทัพให้ไปเอาลิสบทิน ครั้งนั้นเสด็จหนุนทัพขึ้นไปตั้งทัพหลวงตำบลโคน” พระราชพงศาวดาร ฉบับพระราชหัตถเลขา กล่าวถึงเหตุการณ์ในสมัยรัชกาลที่ ๑ ว่า “และทัพสมเด็จพระเจ้าหลานเธอเจ้าฟ้ากรมหลวงเทพหริรักษ์ พระยายมราชยกขึ้นไปทางเมืองลี้ ทางนั้นเป็นทางที่พม่ามาได้” และลิลิตยวนพ่ายกล่าวถึงตอนที่ทัพกรุงศรีอยุธยาของสมเด็จพระบรมไตรโลกนาถ (พ.ศ. ๑๙๙๑ - ๒๐๓๑) ตามไล่ทัพเชียงใหม่ของพระเจ้าติโลกราช (พ.ศ. ๑๙๘๕ - ๒๐๓๐) จากสุโขทัยกลับขึ้นเหนือไปในบทหนึ่งว่า

๏ อยู่ไทธิเบศรเจ้า	จอมปราณ
พราวพฤาบพลคชเสน	เกลื่อนแก้ว
ครั้นนพระผ่าผลาญพล	ยวนย่อย ไปแฮ
ทนนที่น้ำลิบแล้ว	เลอศไชย ฯ

น้ำลิบที่กล่าวนี้นักวิชาการต่างสรุปว่าเป็นเมืองลี้นั่นเอง บริเวณที่สร้างเมืองลี้ในอดีต ปัจจุบันยังคงปรากฏหลักฐานซากกำแพงเมืองให้เห็นบริเวณวัดพระธาตุดวงเดียว วัดพระธาตุห้าดวง วัดพระธาตุแท่นคำ วัดลี้หลวง วัดโปงกาง(ร้าง) ซึ่งวัดดังกล่าวตั้งอยู่บริเวณสองข้างถนนลำพูน-ลี้ (ถนนพหลโยธิน) ในเขตพื้นที่บ้านวังดิน บ้านลี้ บ้านพระธาตุห้าดวง ในเขตตำบลลี้

นอกจากนี้เมืองลี้ยังเป็นเมืองสำคัญที่เป็นศูนย์กลางการคมนาคมเมืองหนึ่ง งานวิทยานิพนธ์ของ ชวิศา ศิริ ในหัวข้อการค้าของอาณาจักรล้านนา ตั้งแต่ต้นพุทธศตวรรษที่ ๑๙ ถึงต้นพุทธศตวรรษที่ ๒๒ ได้สรุปเส้นทางการคมนาคมสู่เมืองต่าง ๆ สู่เชียงใหม่ โดยผ่านเมืองลี้ถึง ๔ เส้นทางหลัก

๑) เชียงตุง/เชียงรุ่ง — เชียงแสน/เชียงราย — เวียงชัย — เซริง(เทิง) — เวียงลอ(เมืองจุน) — เมืองปง — เชียงม่วน — เมืองสอง — ตรอกสลอบ(วังชิ้น) — เมืองเถิน — เมืองลี้ — เวียงท่ากาน(สันป่าตอง) — เชียงใหม่

๒) ไชยะบูลี /หลวงพระบาง — น่าน — เวียงสา นาน้อย — เวียงแพร่ — ตรอกสลอบ(วังชิ้น) — เมืองเถิน — เมืองลี้ —

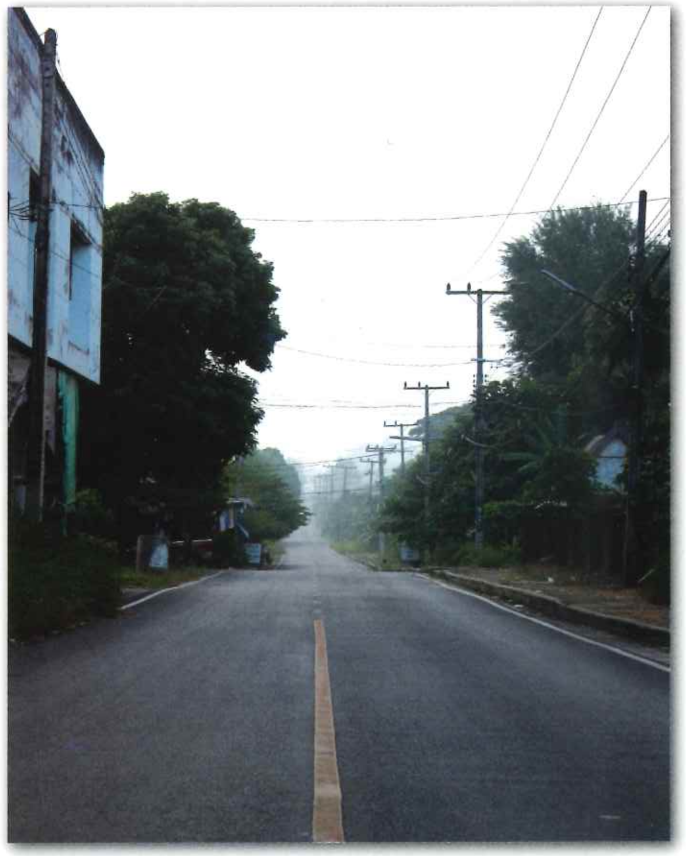

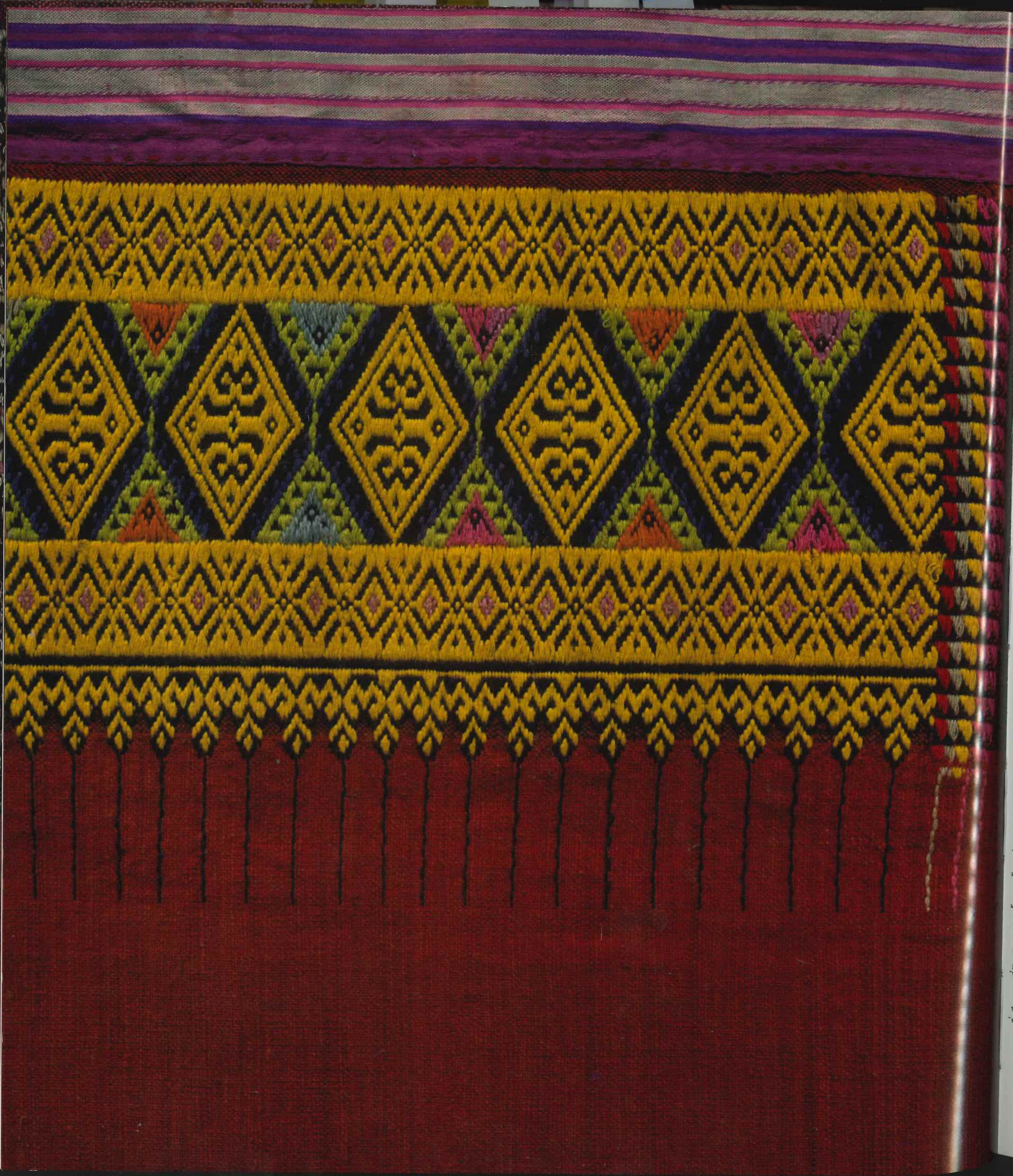

▲ ซิ่นตีนจกพบที่บ้านป่าหก ตำบลลี้ อำเภอลี้ ตัวซิ่นทอจากเส้นไหม

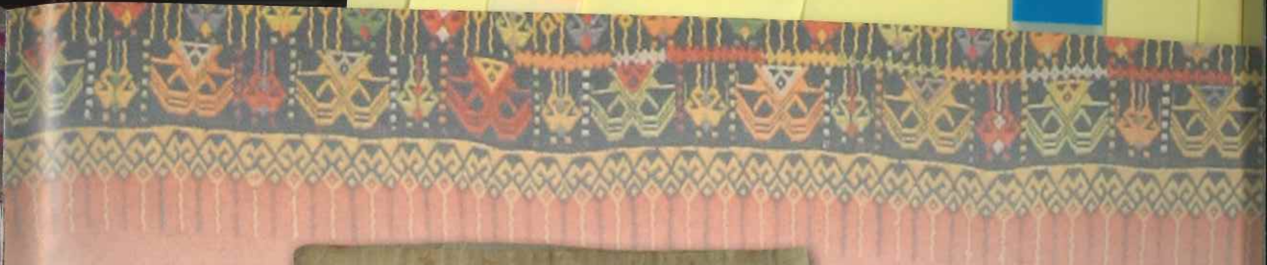

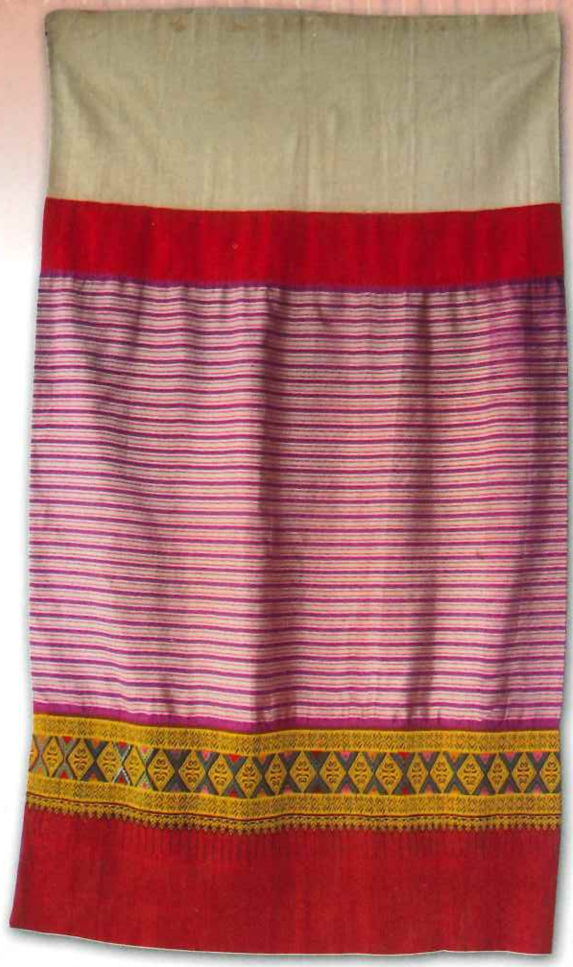

▲ ซิ่นตีนจกพบที่บ้านป่าหก ตำบลลี้ อำเภอลี้ ตัวซิ่นทอจากเส้นไหม

ตำแหน่ง
แถบบนและแถบล่าง
ขัน
โคม

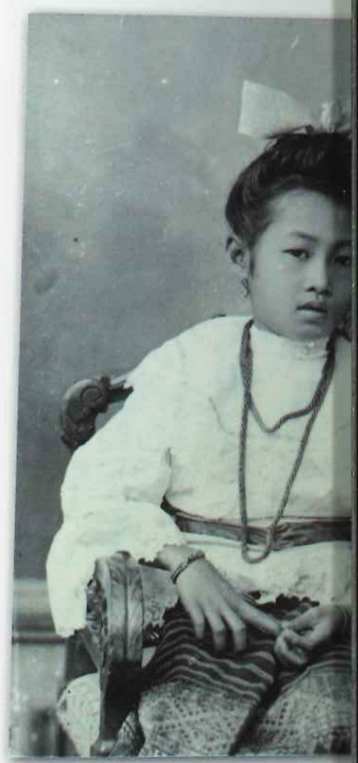

▶ เด็กหญิงชาวสยามไม่ทราบนาม ทั้งคู่นุ่งซิ่นตีนจกไทยวน (สำนักหอจดหมายเหตุแห่งชาติ)

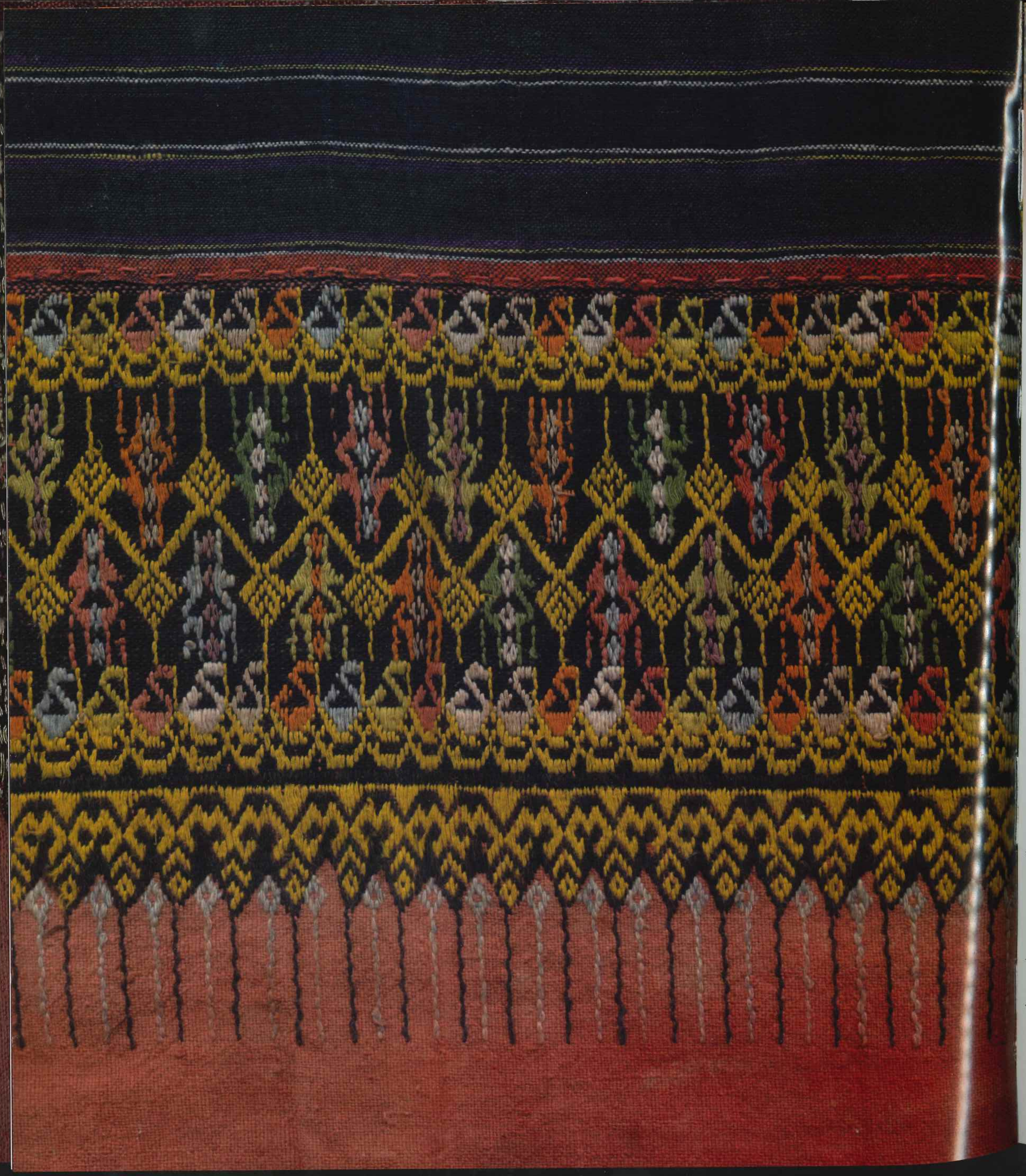

▲ ซิ่นตีนจกพบที่บ้านบวก ตำบลดงคำ อำเภอลี้

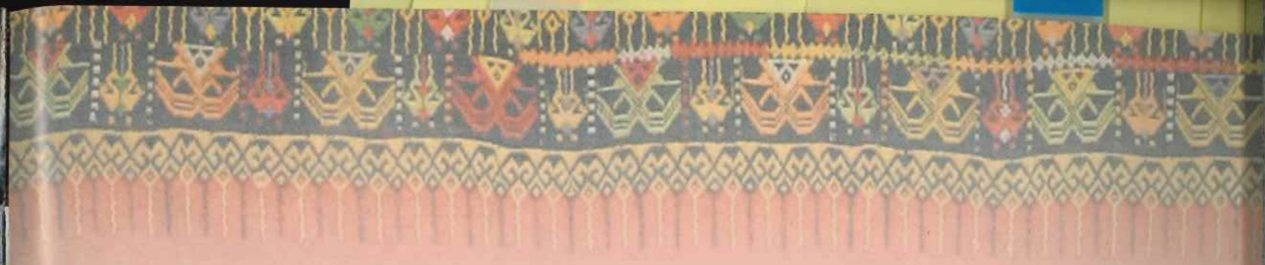

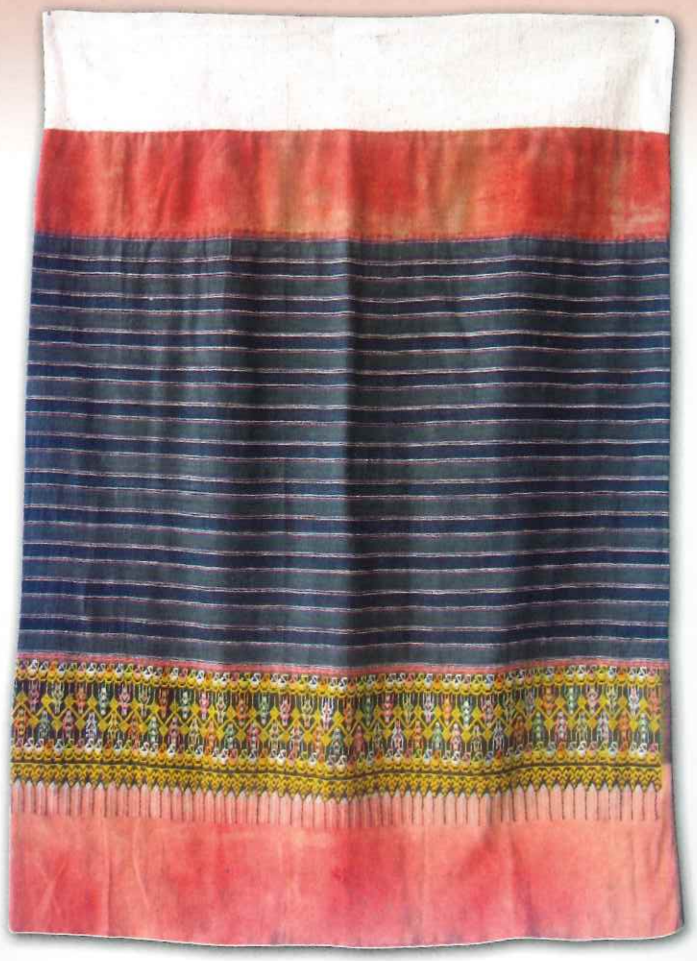

▲ ซิ่นตีนจกพบที่บ้านบวก ตำบลดงคำ อำเภอลี้

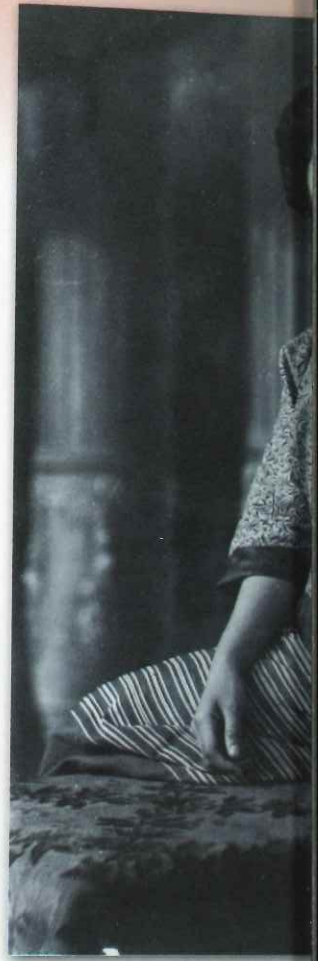

▲ เจ้าลดาคำ ณ เชียงใหม่ (๒๔๓๙-๒๕๓๗)

ตำแหน่ง	แบบสมมาตร
แถบบนและแถบล่าง	*p111*
ขัน โคม	-
แถบกลาง	*pma2*

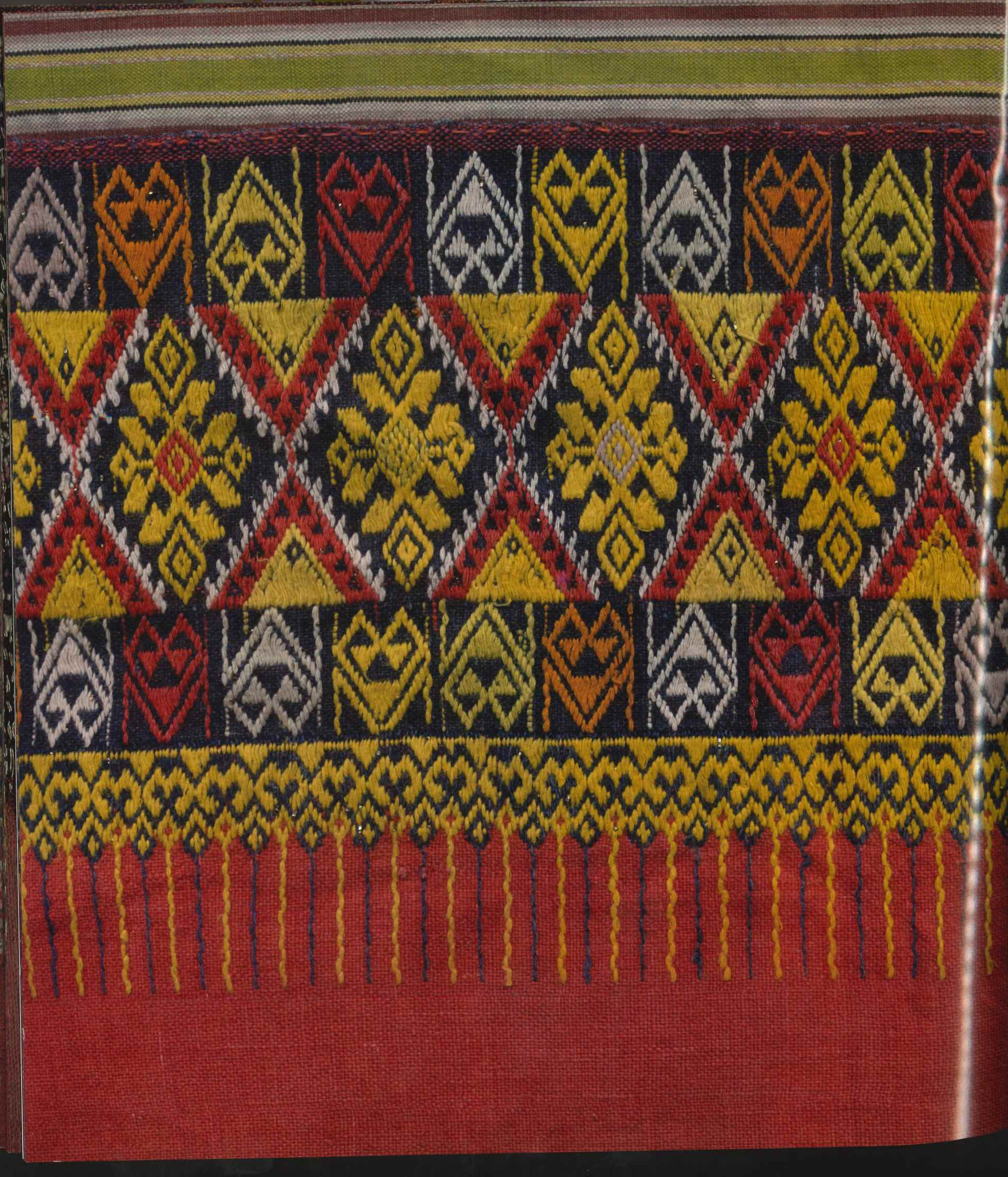

▲ ซิ่นตีนจกพบที่บ้านวังดิน ตำบลลี้ อำเภอลี้

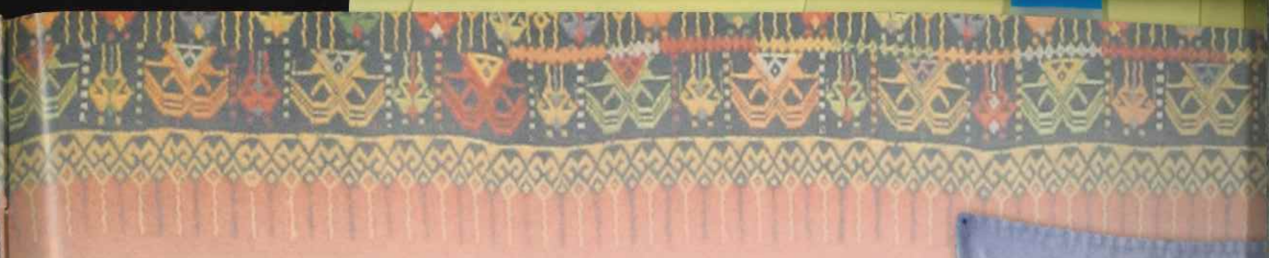

ตำแหน่ง	แบบสมมาตร
แถบบนและแถบล่าง	*pma2*
ขัน	*pmm2*
โคม	*pmm2*

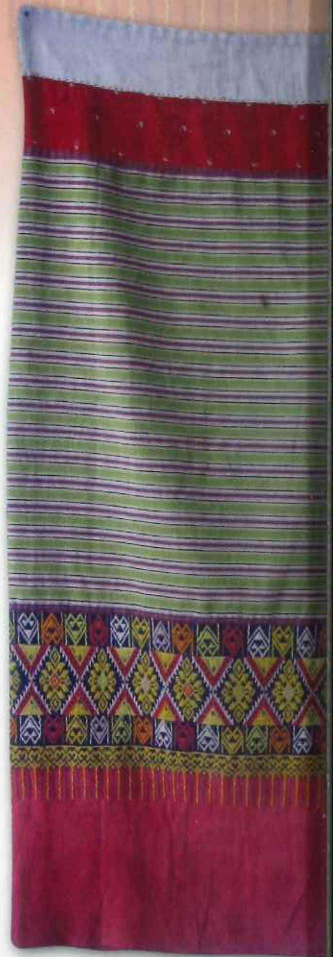

▶ ซิ่นตีนจกพบที่บ้านวังดิน ตำบลลี้ อำเภอลี้

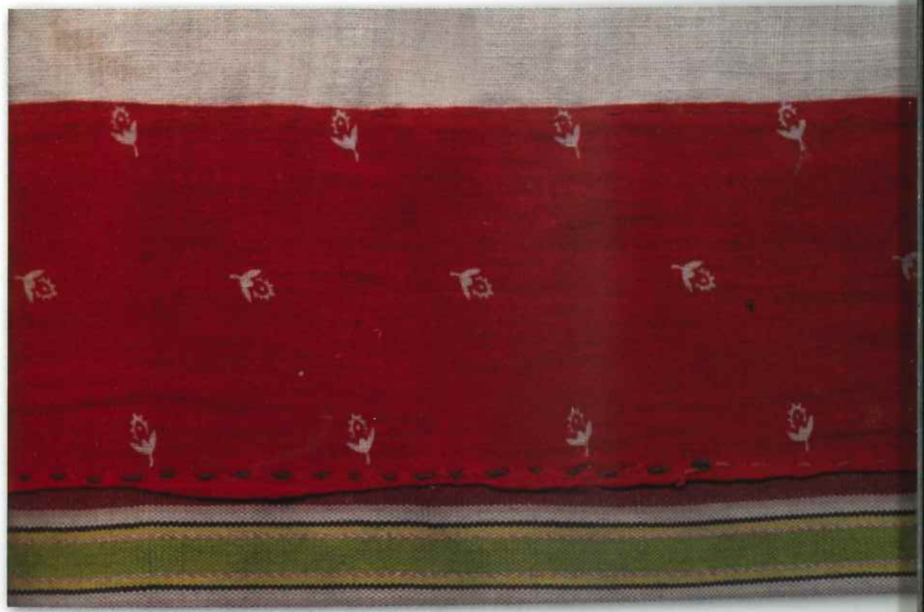

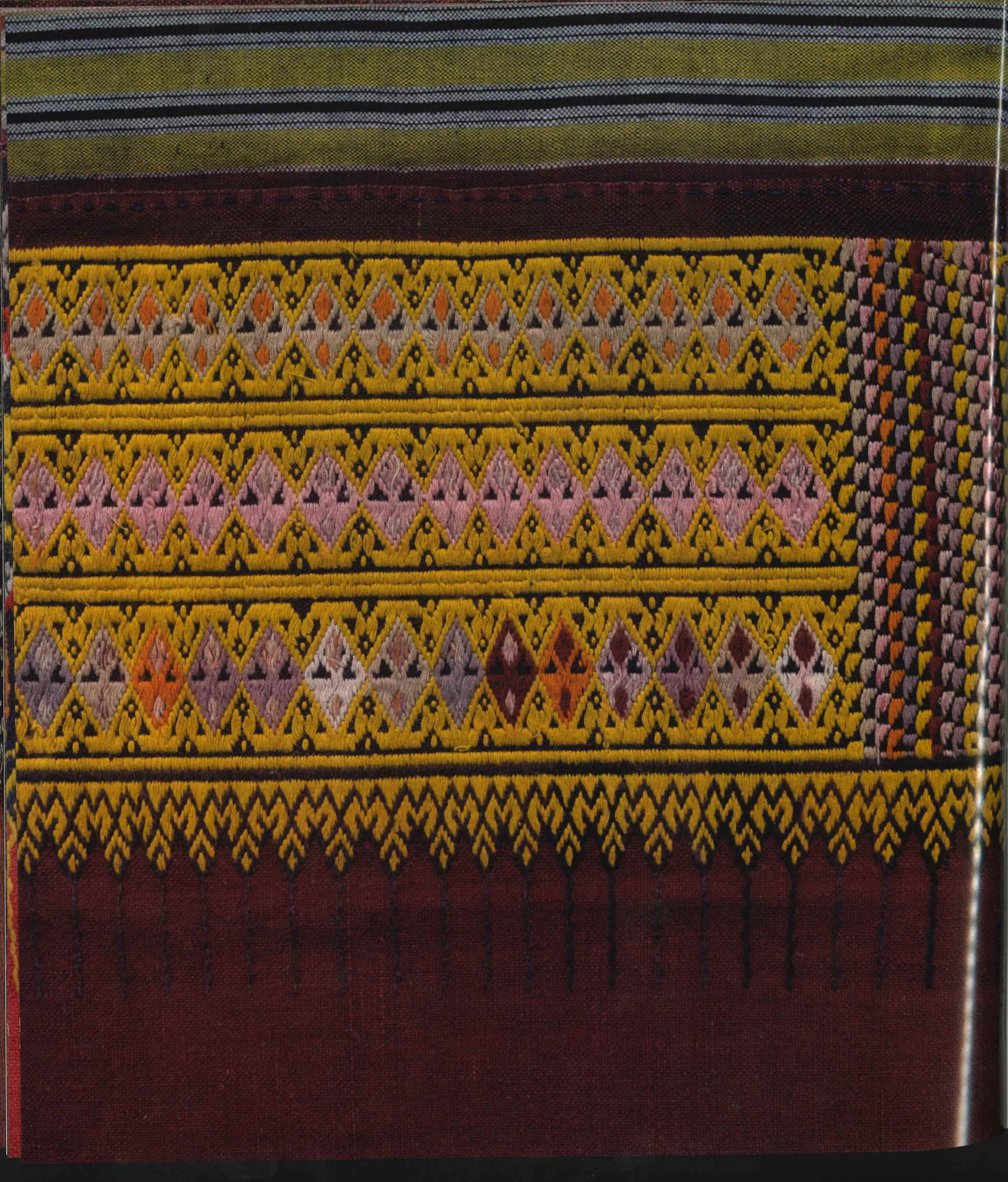

▲ ซิ่นตีนจก ซิ่นตีนจกพบที่บ้านโฮ่ง ตำบลลี้ อำเภอลี้

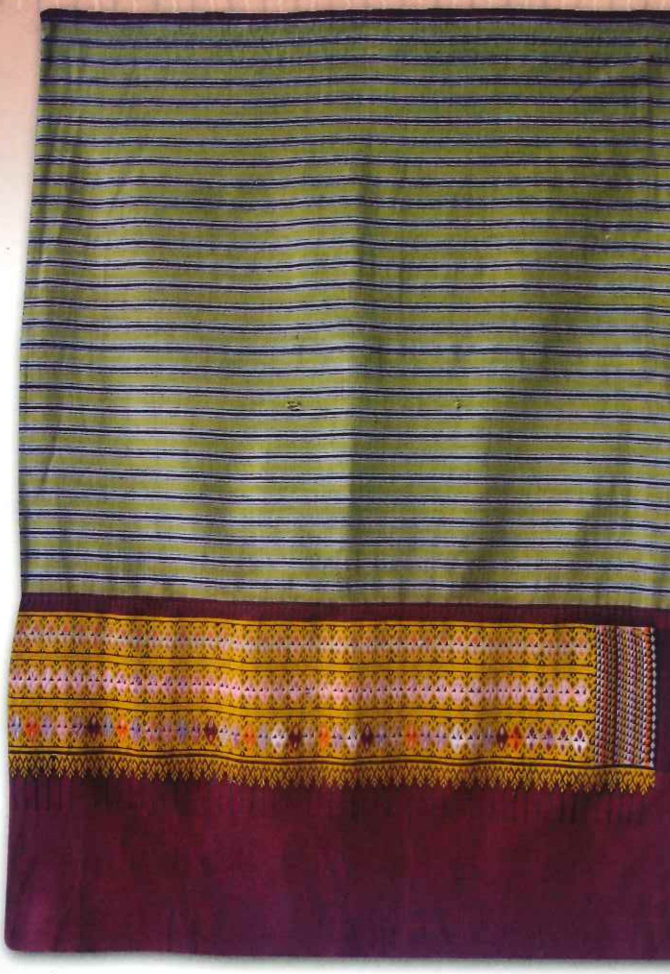

▲ ซิ่นตีนจก ซิ่นตีนจกพบที่บ้านโฮ่ง ตำบลลี้ อำเภอลี้

ตำแหน่ง
แถบบน
แถบกลาง
แถบล่าง

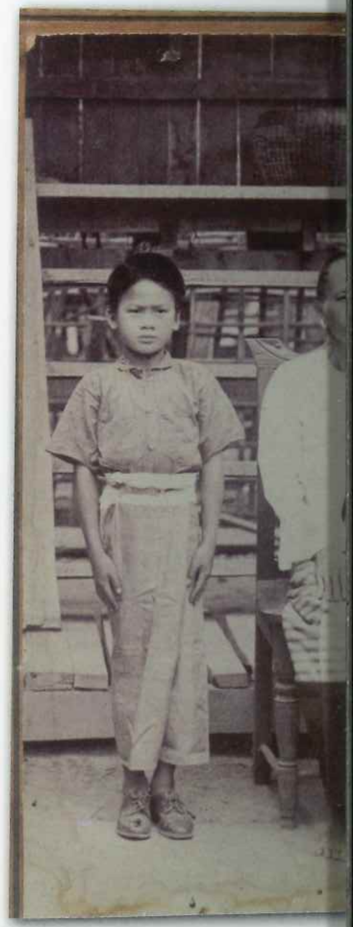

► แม่เฒ่าเมืองลี้ ขนาบข้างด้วยหลานบ่าวในชุดแต่งกายเรียบร้อย (บุญยวงคอลเลคชั่น)

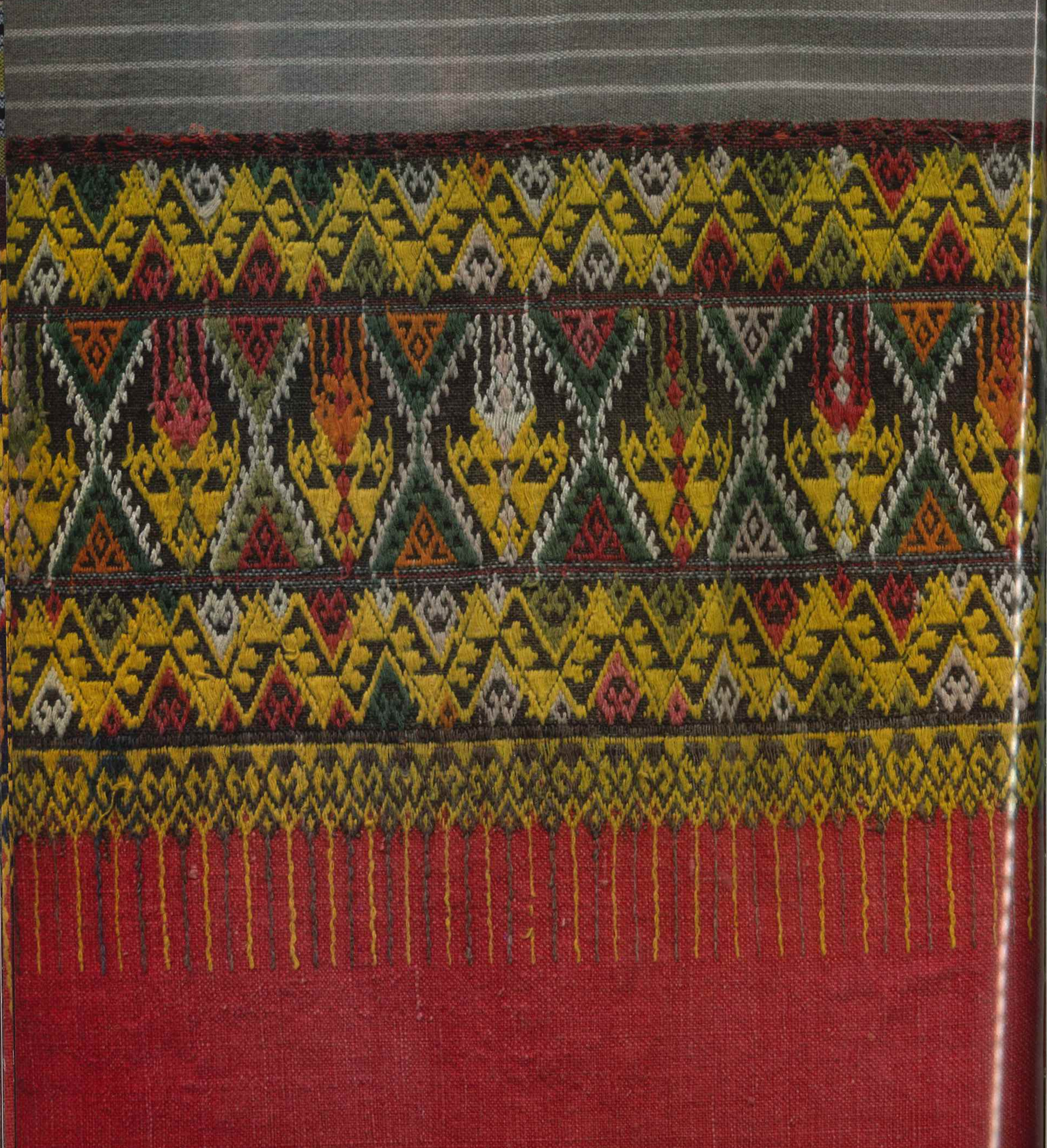

▲ ซิ่นตีนจกพบที่บ้านลี้ ตำบลลี้ อำเภอลี้

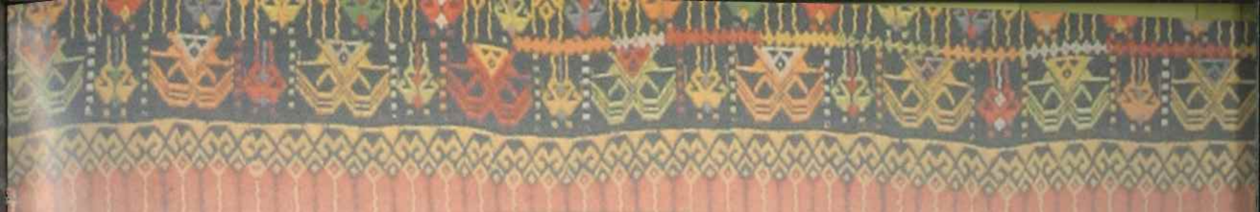

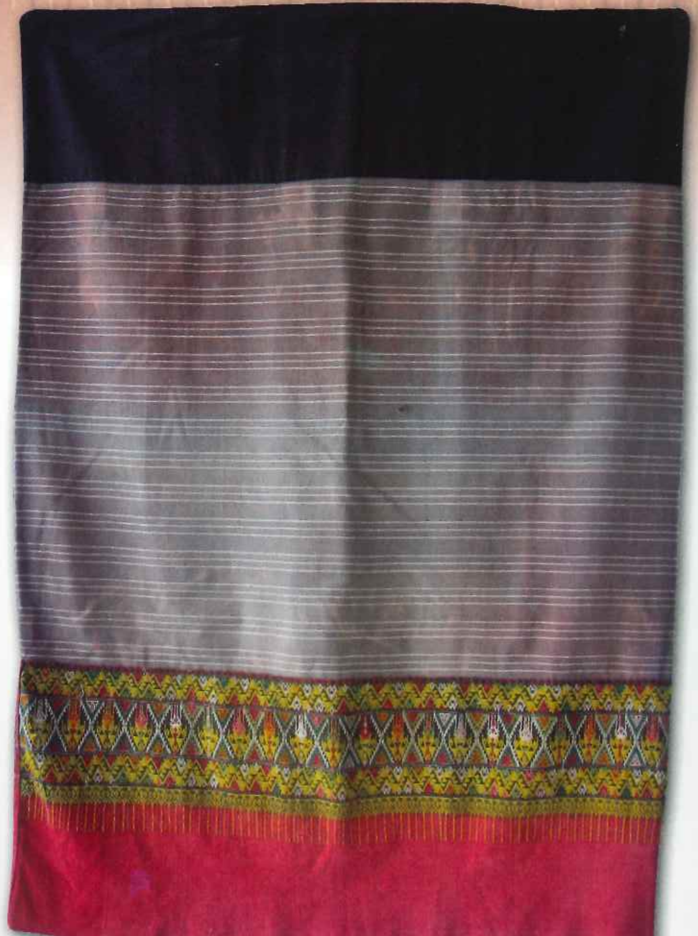

▲ ซิ่นตีนจกพบที่บ้านลี้ ตำบลลี้ อำเภอลี้

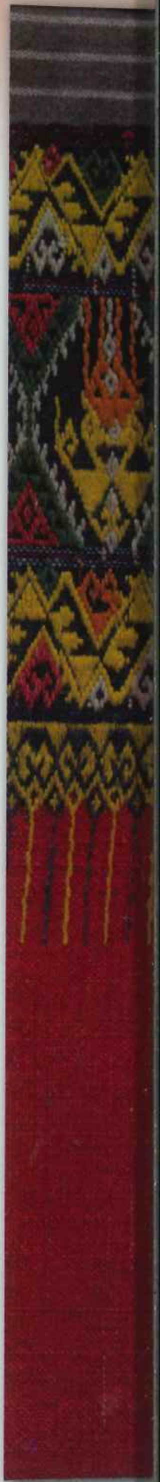

ตำแหน่ง	แบบสมมาตร
แถบบนและแถบล่าง	*p1a1*
ขัน	*pmm2*
โคม	*pm11*

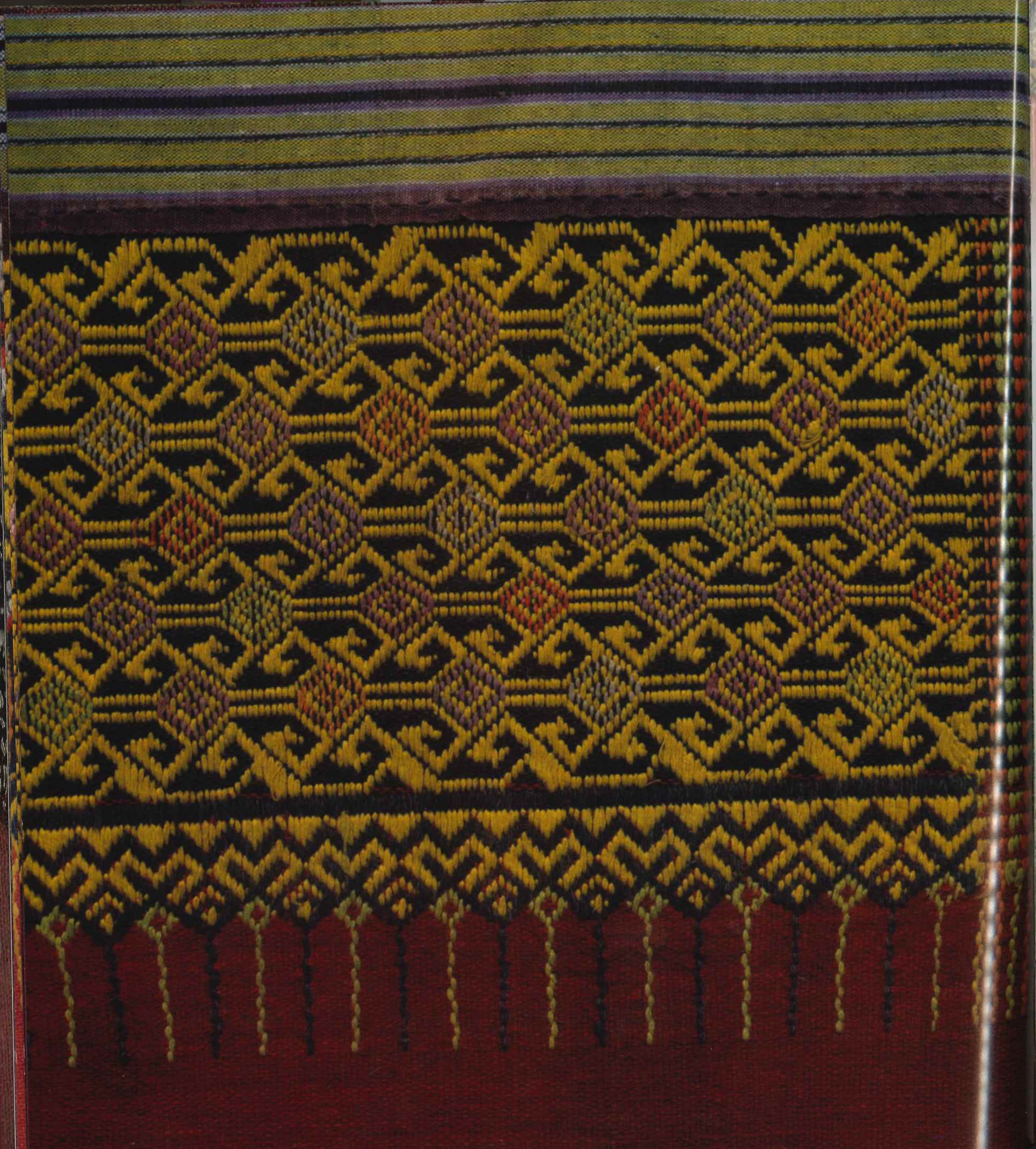

▲ ซิ่นตีนจก พบที่บ้านปางส้าน ตำบลดงดำ อำเภอลี้

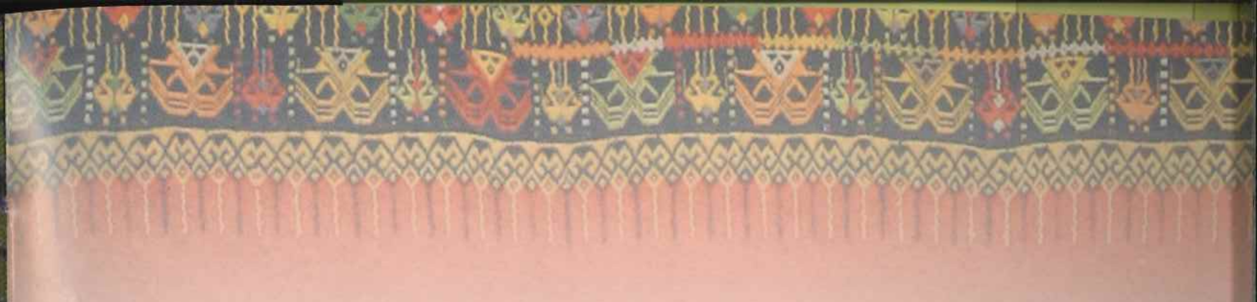

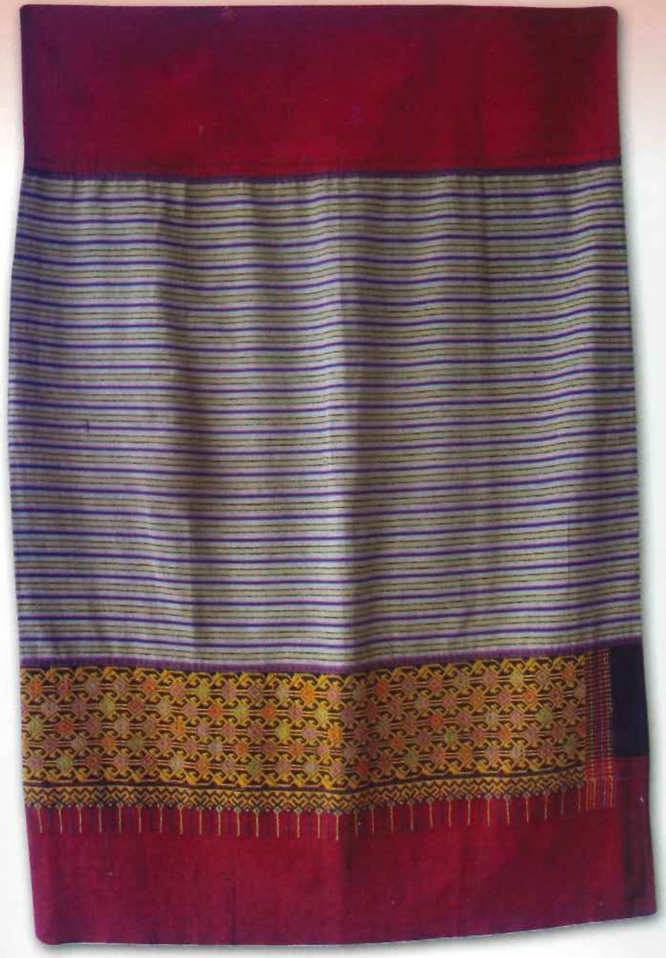

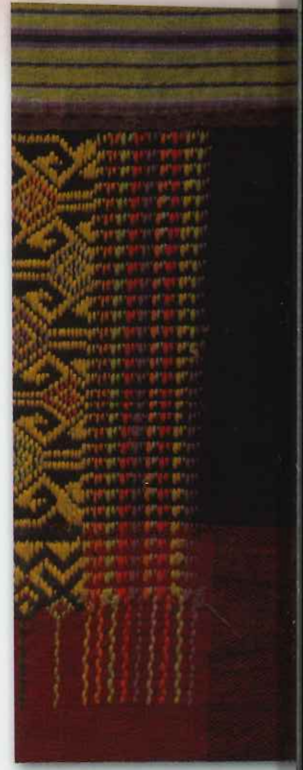

▲ ซิ่นตีนจก พบที่บ้านปางส้าน ตำบลดงดำ อำเภอลี้

ตำแหน่ง	แบบสมมาตร
แถบบน	*p1m1*
แถบกลาง	*p1m1*
แถบล่าง	*p1m1*

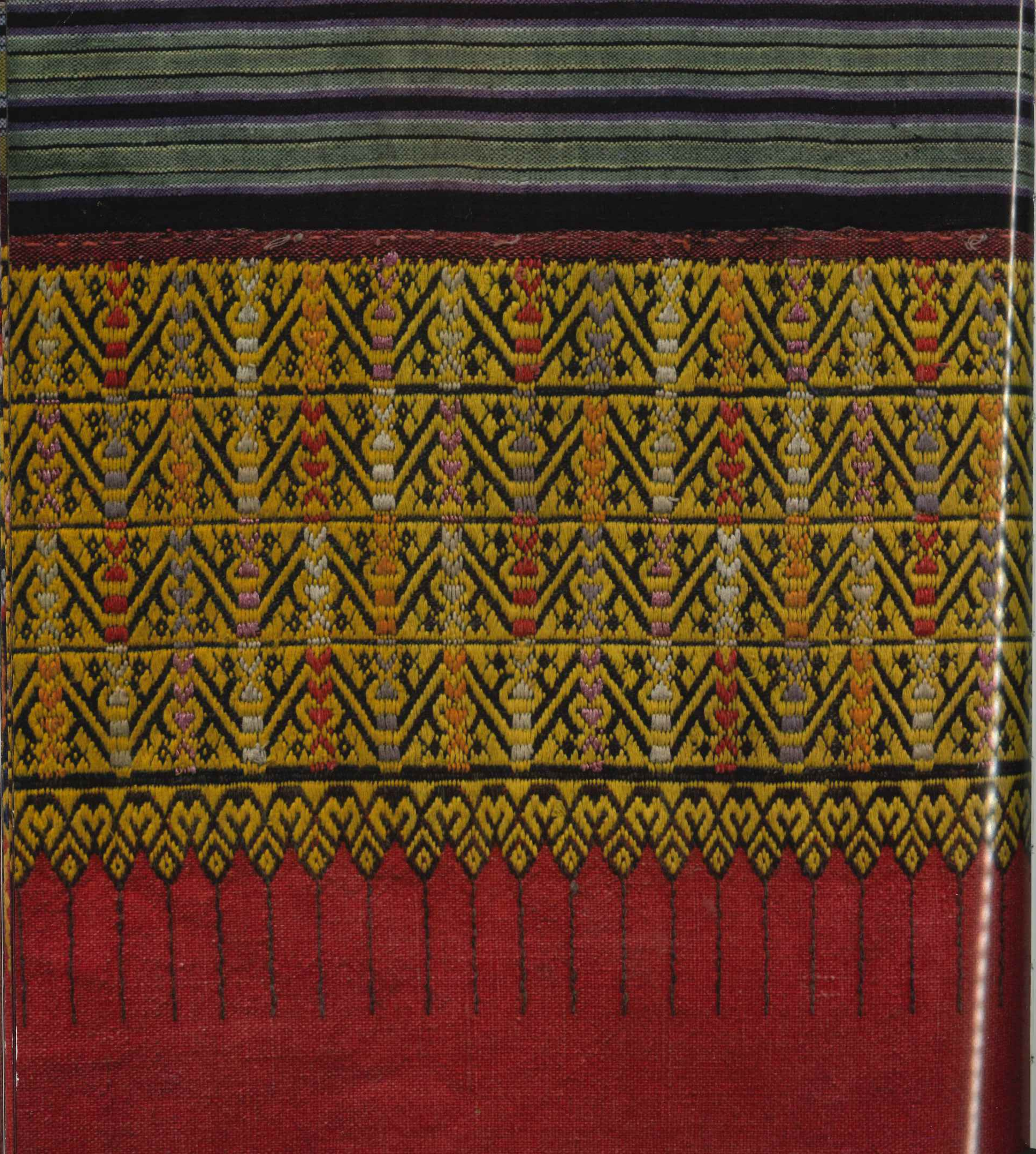

▲ ซิ่นตีนจก พบที่บ้านปางส้าน ตำบลดงดำ อำเภอลี้

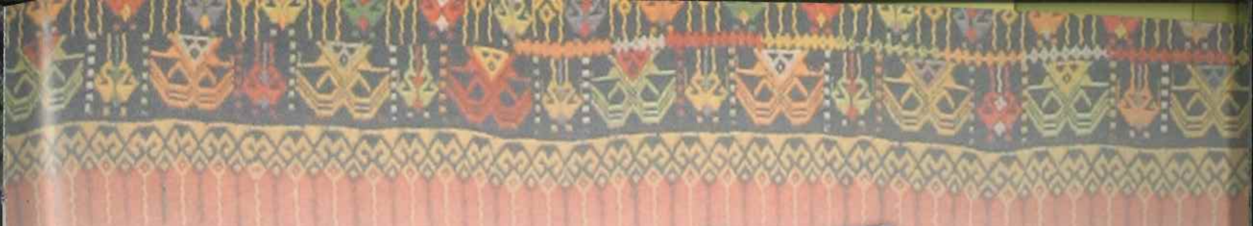

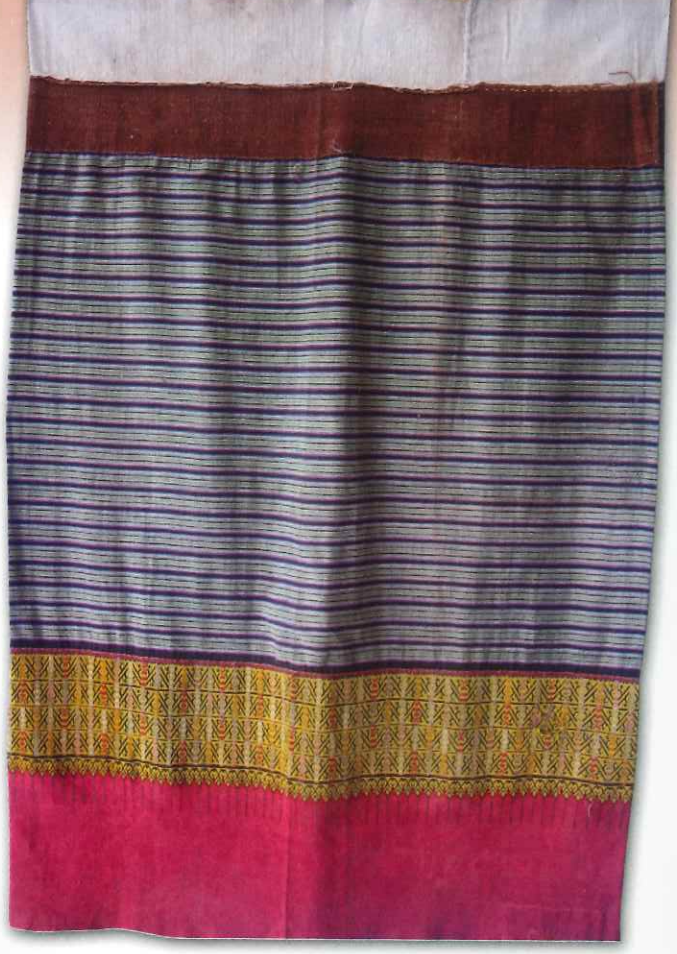

▲ ซิ่นตีนจก พบที่บ้านปางส้าน ตำบลดงดำ อำเภอลี้

ตำแหน่ง
แถบบน
แถบกลาง
แถบล่าง

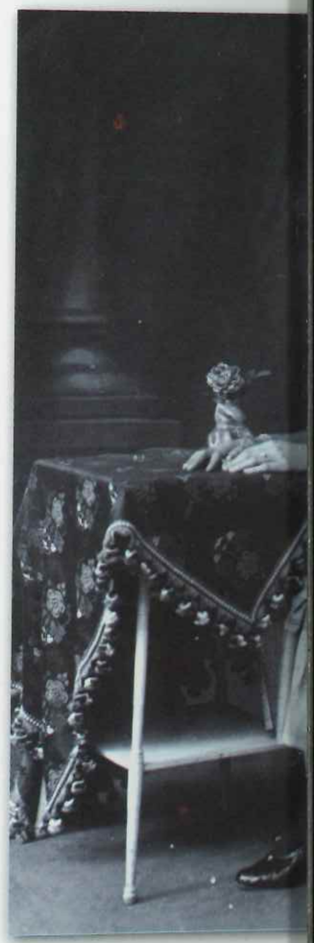

► สตรีชาวสยามไม่ทราบนามใช้ซิ่นลายขวางแบบไทยวนในการแต่งกายสมัยรัชกาลที่ ๖
(สำนักหอจดหมายเหตุแห่งชาติ)

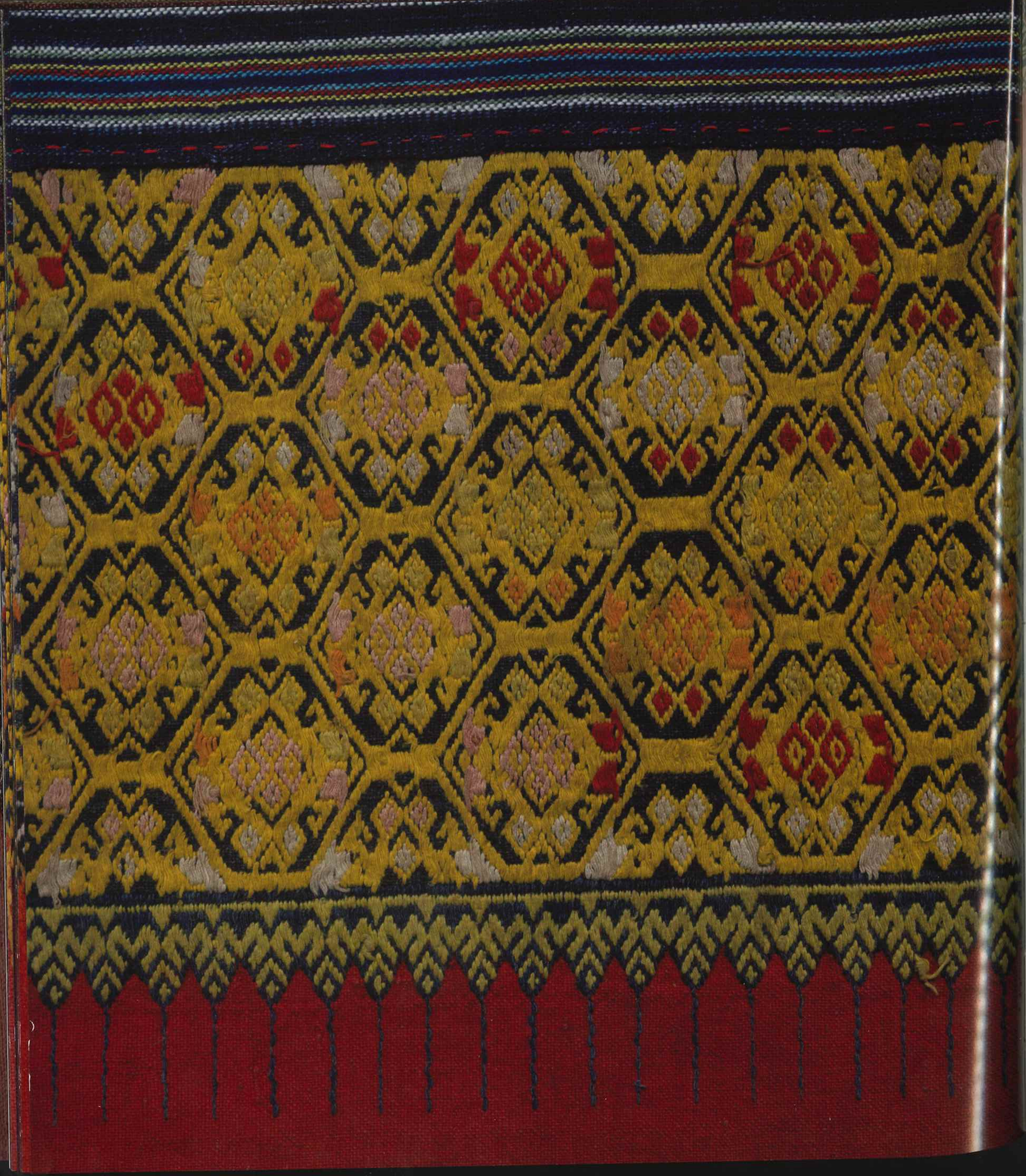

▲ ซิ่นตีนจกพบที่บ้านห้วยหญ้าไซ ตำบลดงดำ อำเภอลี้

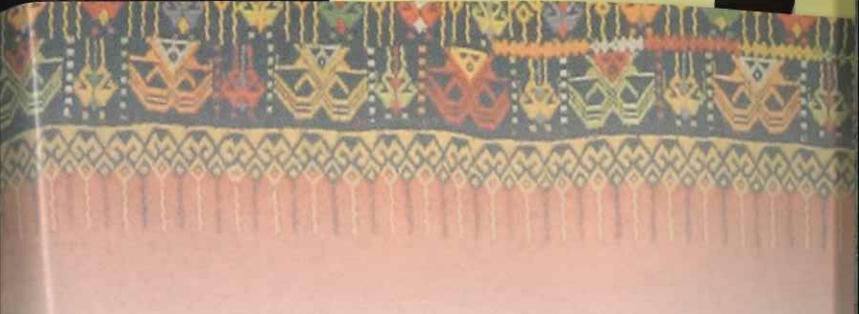

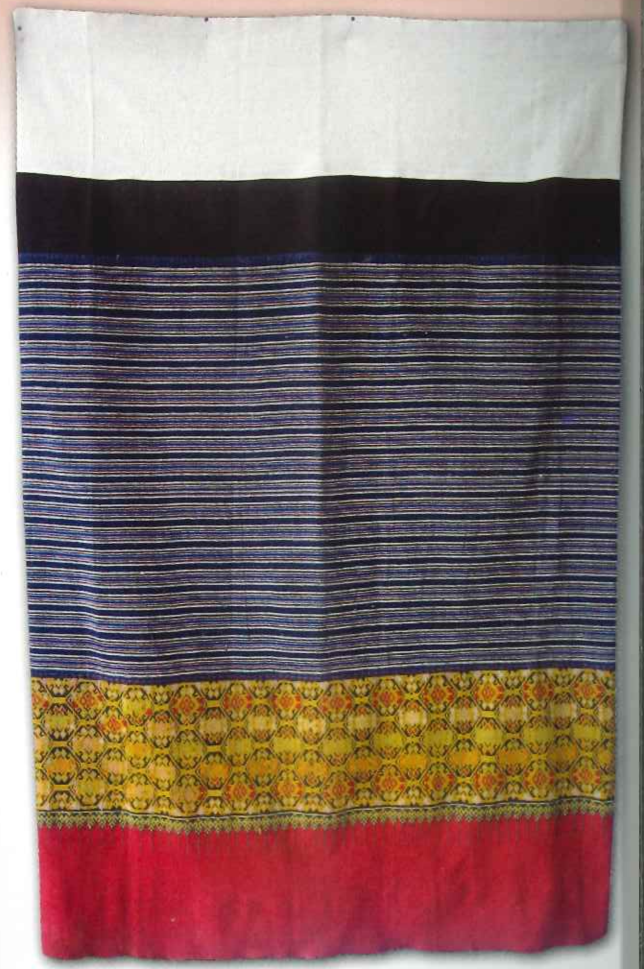

▲ ซิ่นตีนจกพบที่บ้านห้วยหญ้าไซ ตำบลดงดำ อำเภอลี้

ตำแหน่ง
แถบบนและแถบล่าง
ขัน
โคม

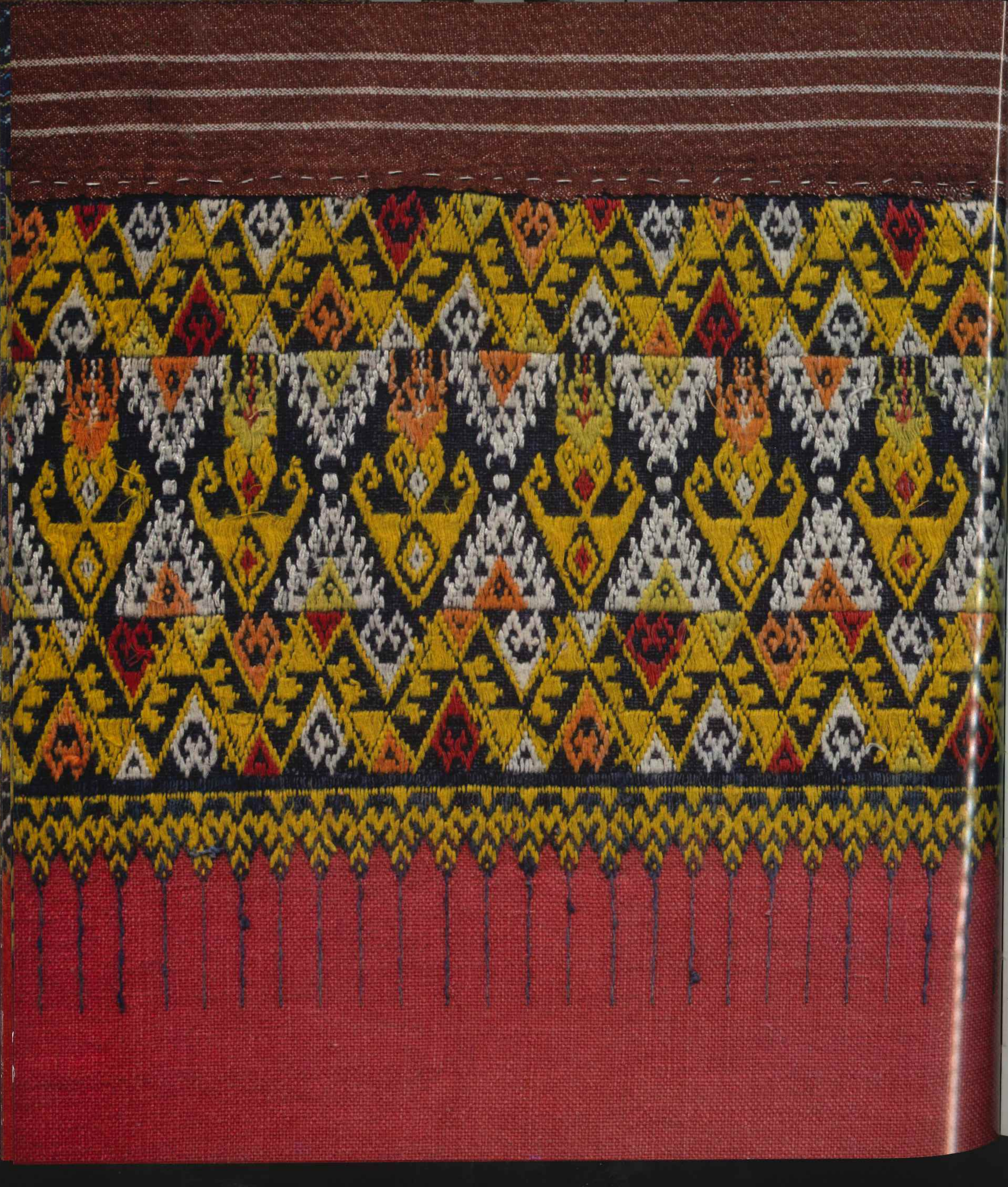

▲ ซิ่นตีนจกพบที่บ้านม่วงสามปี ตำบลลี้ อำเภอลี้

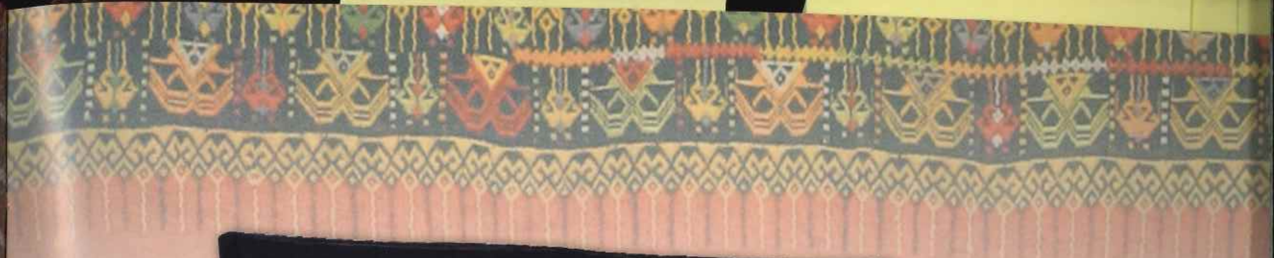

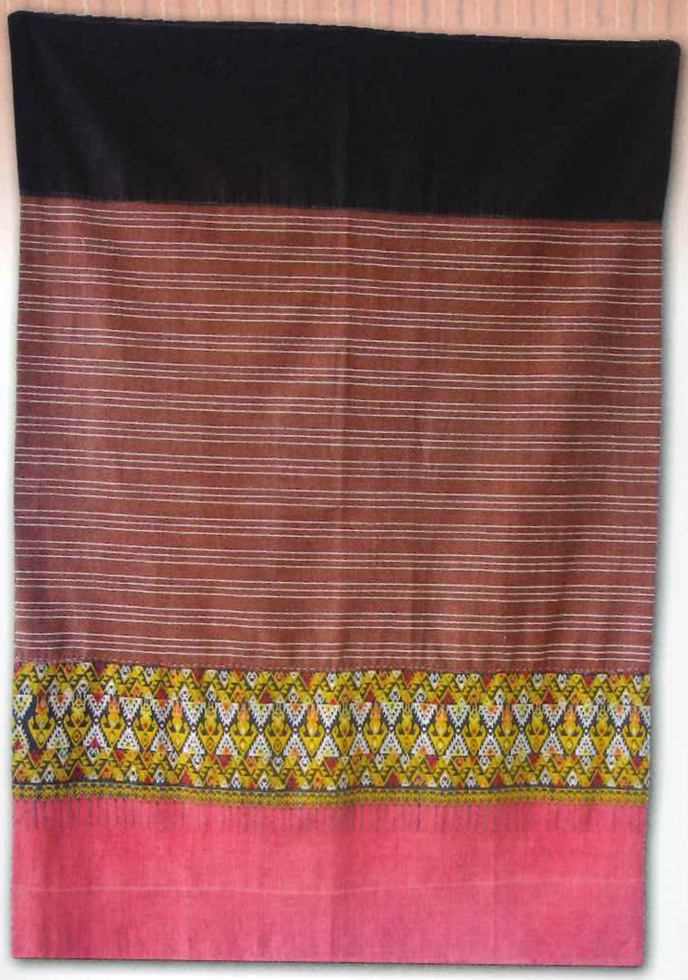

▲ ซิ่นตีนจกพบที่บ้านม่วงสามปี ตำบลลี้ อำเภอลี้

ตำแหน่ง
แถบบนและแถบล่าง
ขัน
โคม

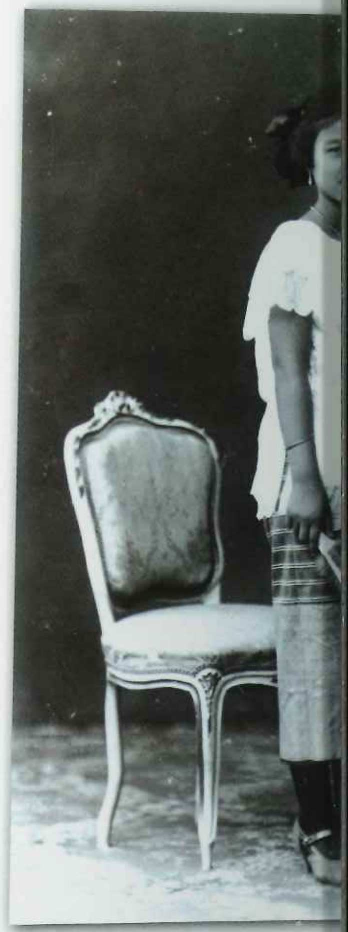

▶ สตรีชาวสยามไม่ทราบนาม (สำนักหอจดหมายเหตุแห่งชาติ)

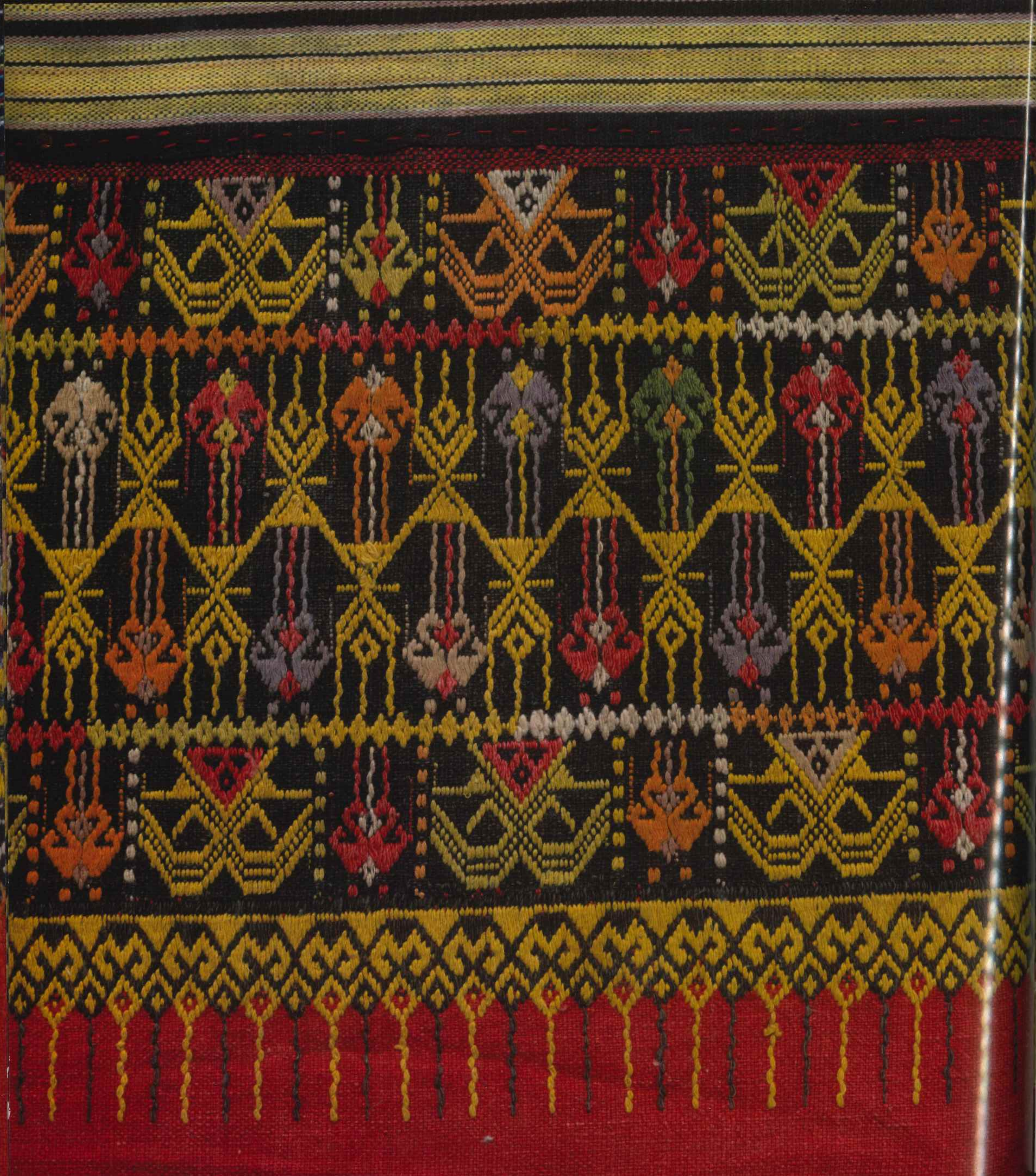

▲ ซิ่นตีนจกพบที่บ้านพวงคำ ตำบลลี้ อำเภอลี้

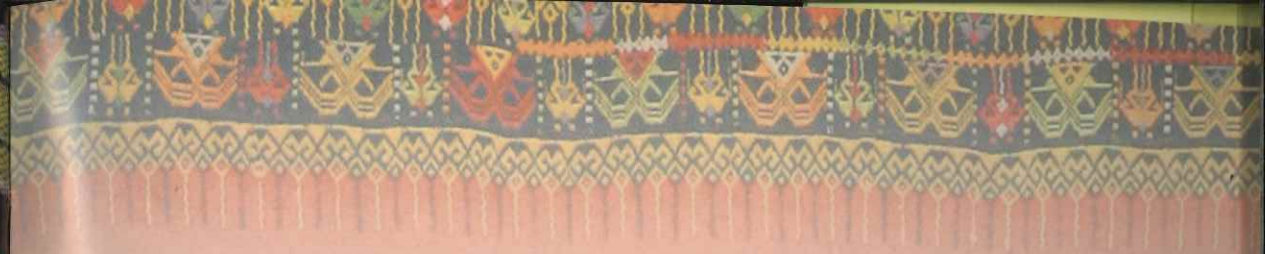

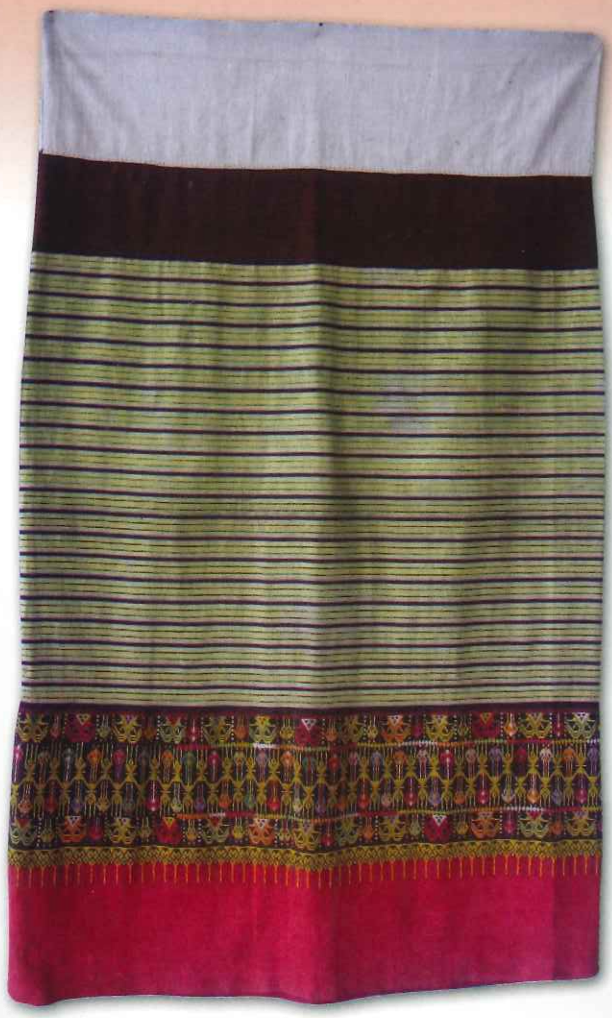

▲ ซิ่นตีนจกพบที่บ้านพวงคำ ตำบลลี้ อำเภอลี้

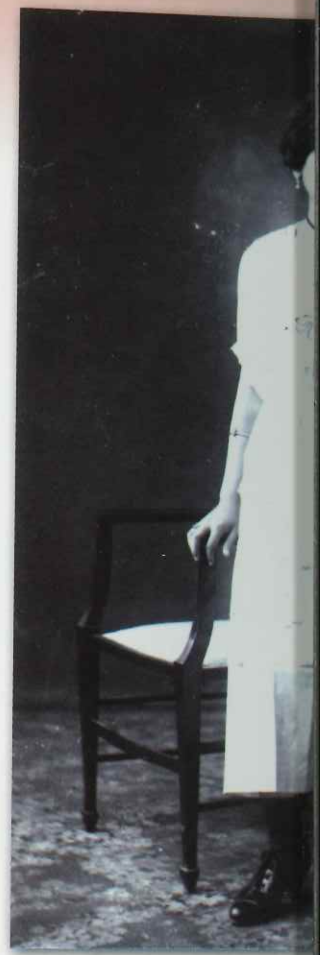

▲ สตรีชาวสยามไม่ทราบนาม (สำนักหอจดห[illegible]

ตำแหน่ง	แบบสมมาตร
แถบบน	*pm11*
แถบกลาง	*pma2*
แถบล่าง	*pm11*

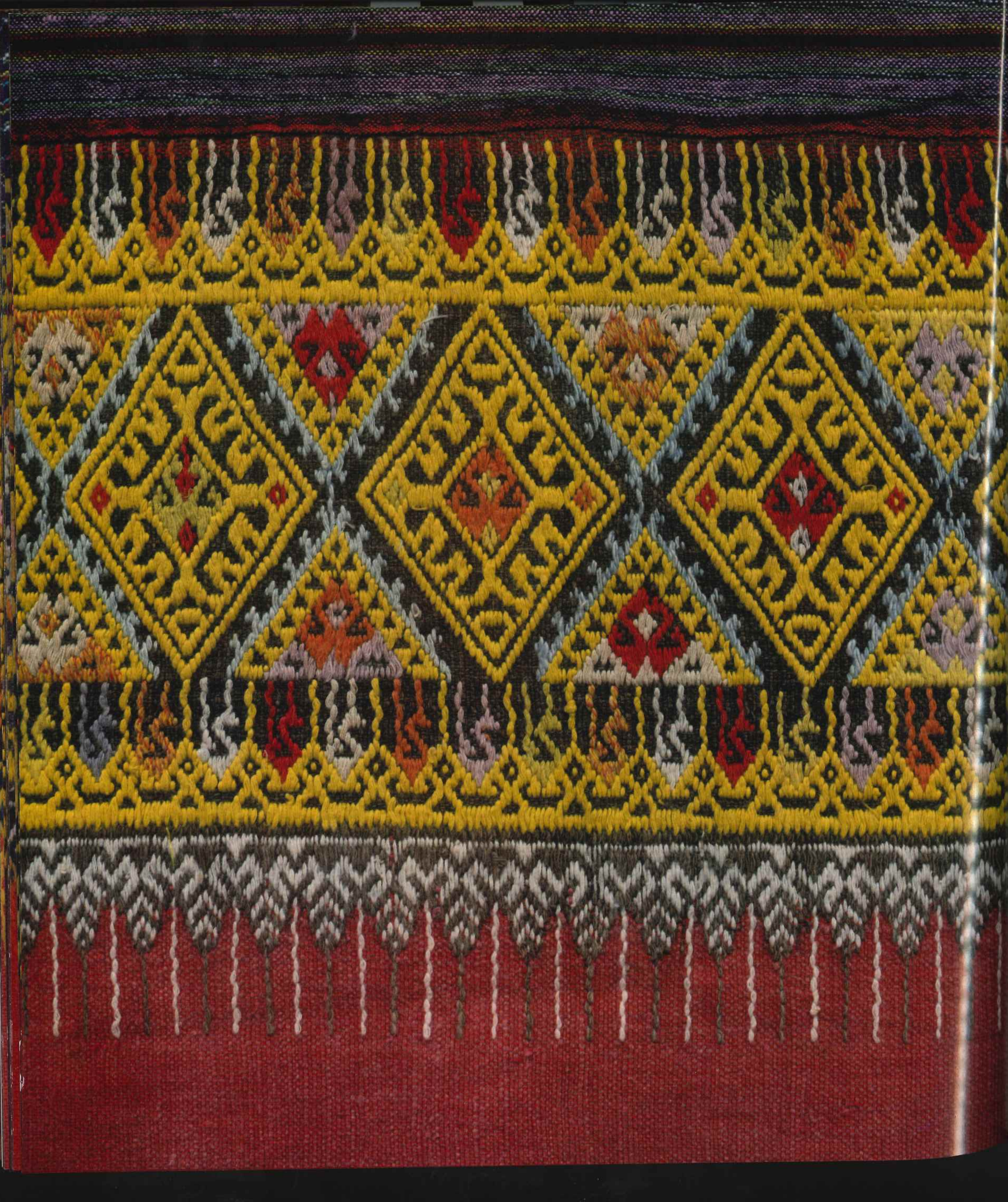

▲ ซิ่นตีนจกพบที่บ้านป่าหก ตำบลลี้ อำเภอลี้

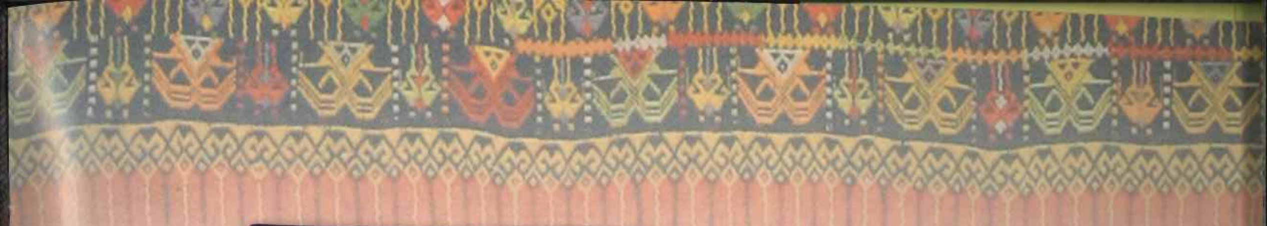

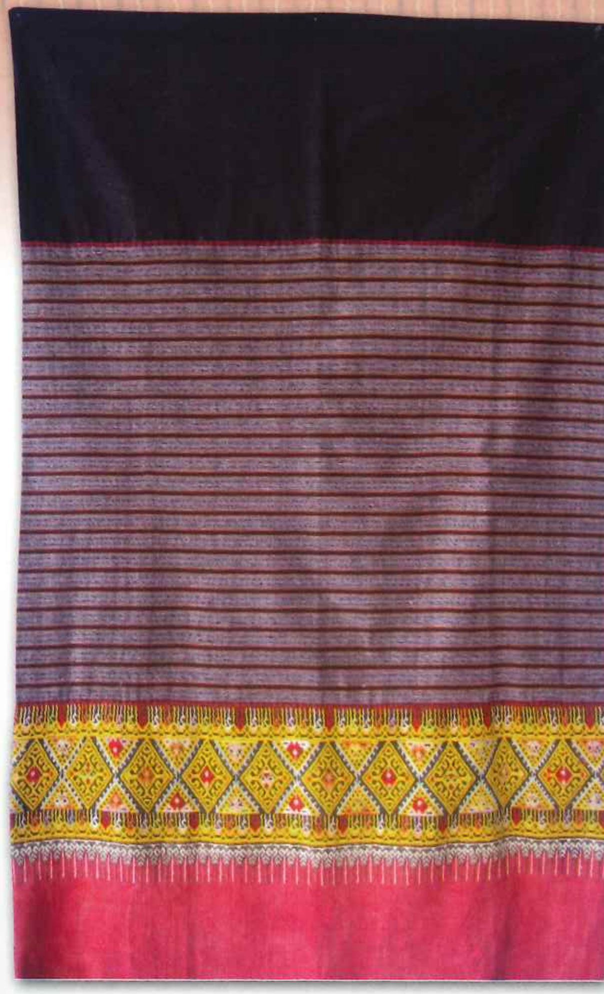

▲ ซิ่นตีนจกพบที่บ้านป่าหก ตำบลลี้ อำเภอลี้

ตำแหน่ง
แถบบนและแถบล่าง
ขัน
โคม

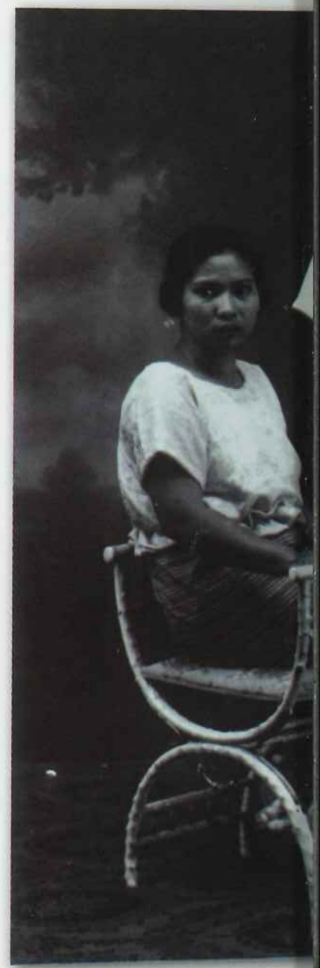

▶ กลุ่มสตรีชาวสยามไม่ทราบนาม (สำนักหอจดหมายเหตุแห่งชาติ)

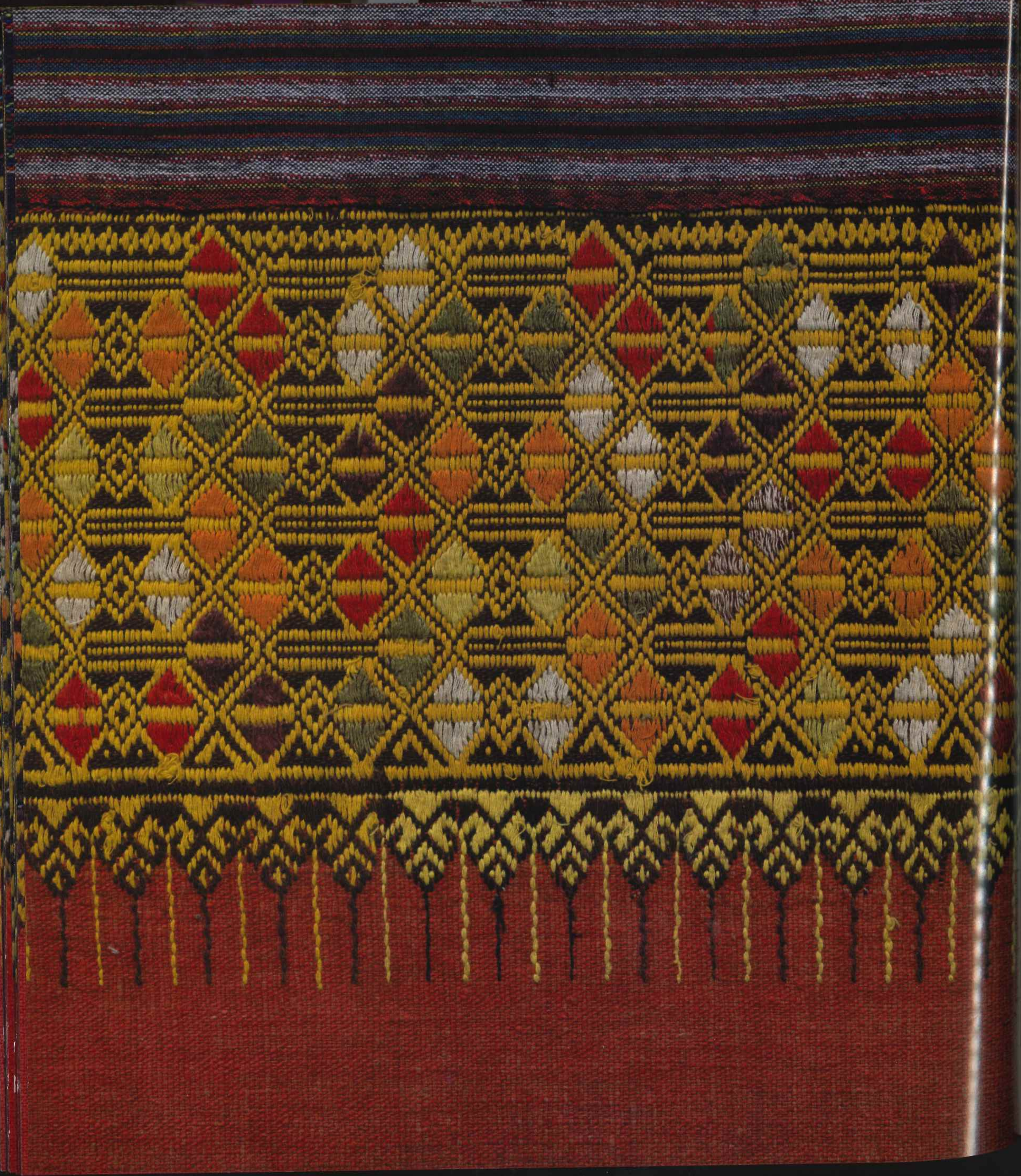

▲ ซิ่นตีนจก พบที่บ้านม่วงสามปี ตำบลลี้ อำเภอลี้

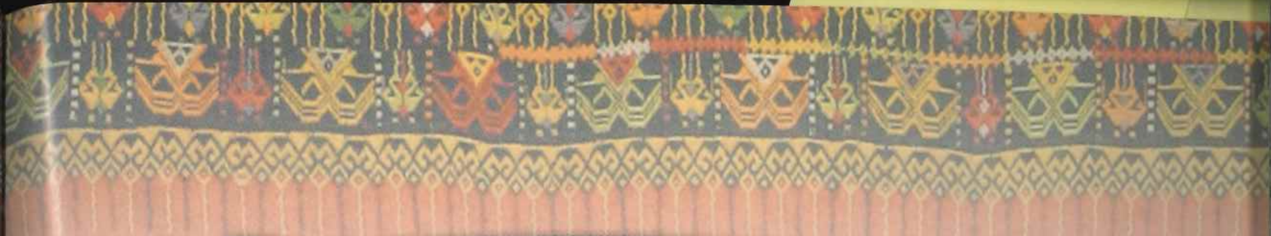

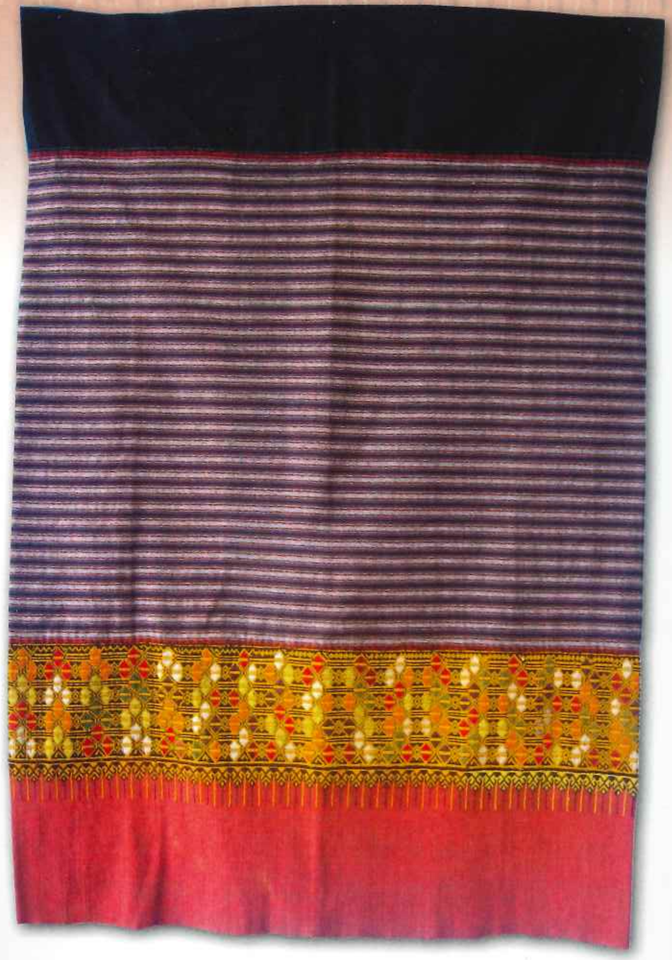

▲ ซิ่นตีนจก พบที่บ้านม่วงสามปี ตำบลลี้ อำเภอลี้

ตำแหน่ง
แถบบน
แถบกลาง
แถบล่าง

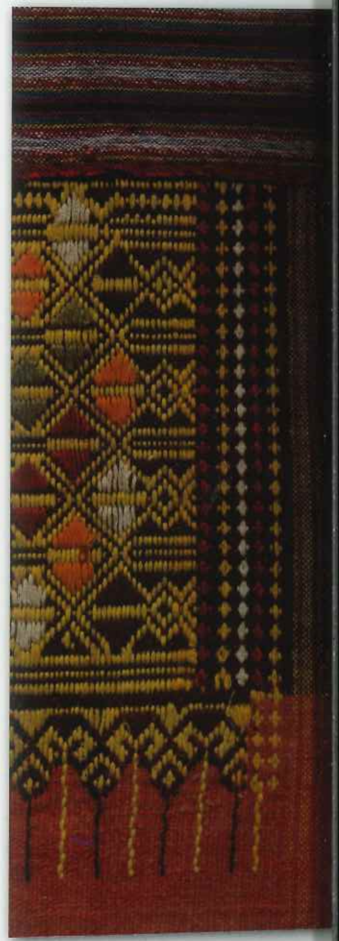

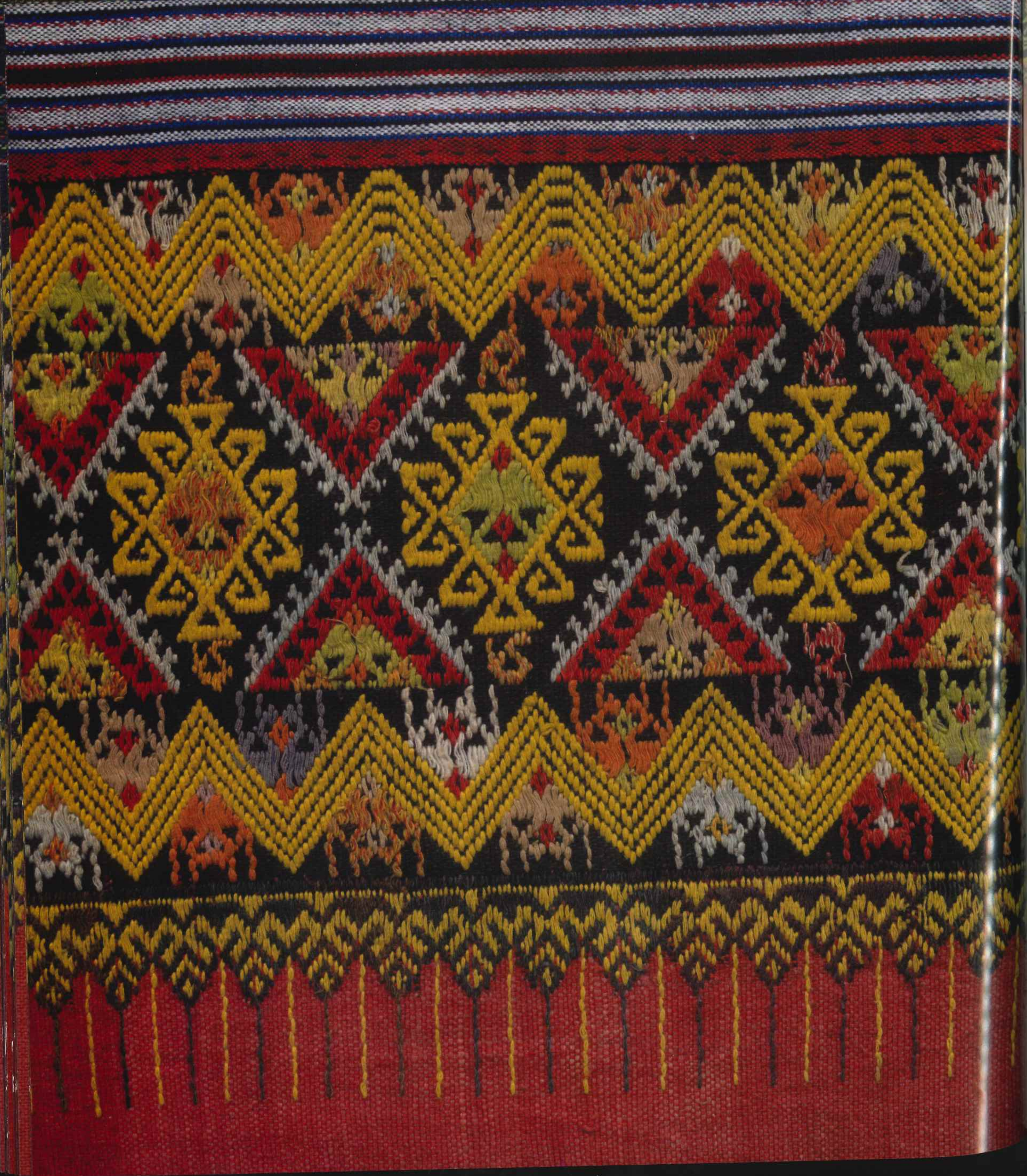

▲ *ซิ่นตีนจก พบที่บ้านบวก ตำบลดงดำ อำเภอลี้*

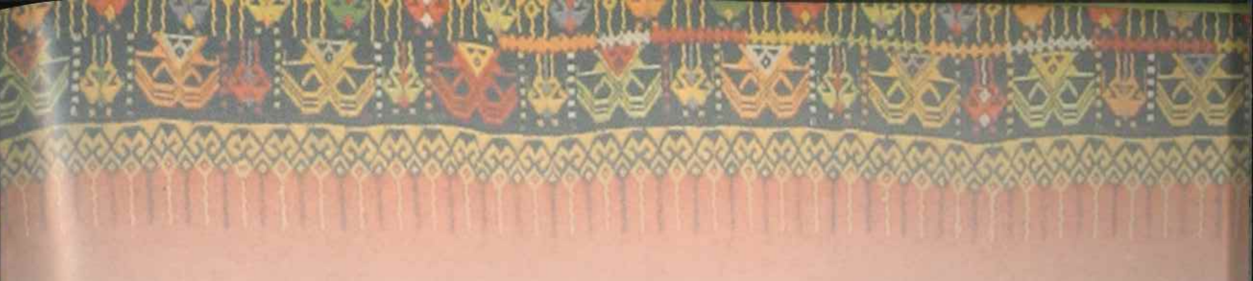

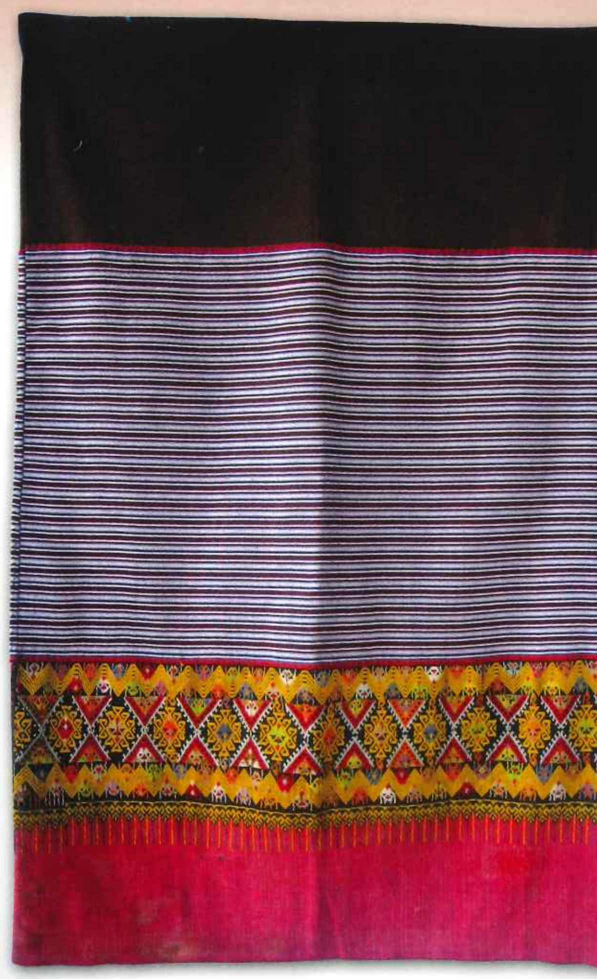

▲ *ซิ่นตีนจก พบที่บ้านบวก ตำบลดงดำ อำเภอลี้*

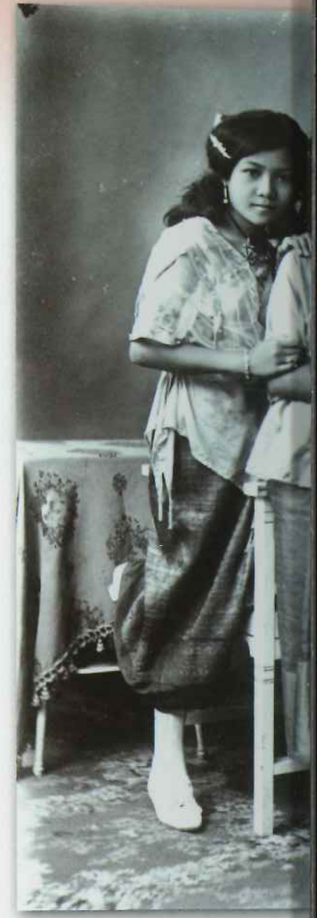

▲ *กลุ่มสตรีชาวสยามไม่ทราบนาม (สำนักห[illegible]*

ตำแหน่ง	แบบสมมาตร
แถบบนและแถบล่าง	*pma2*
ขัน	*pmm2*
โคม	*pm11*

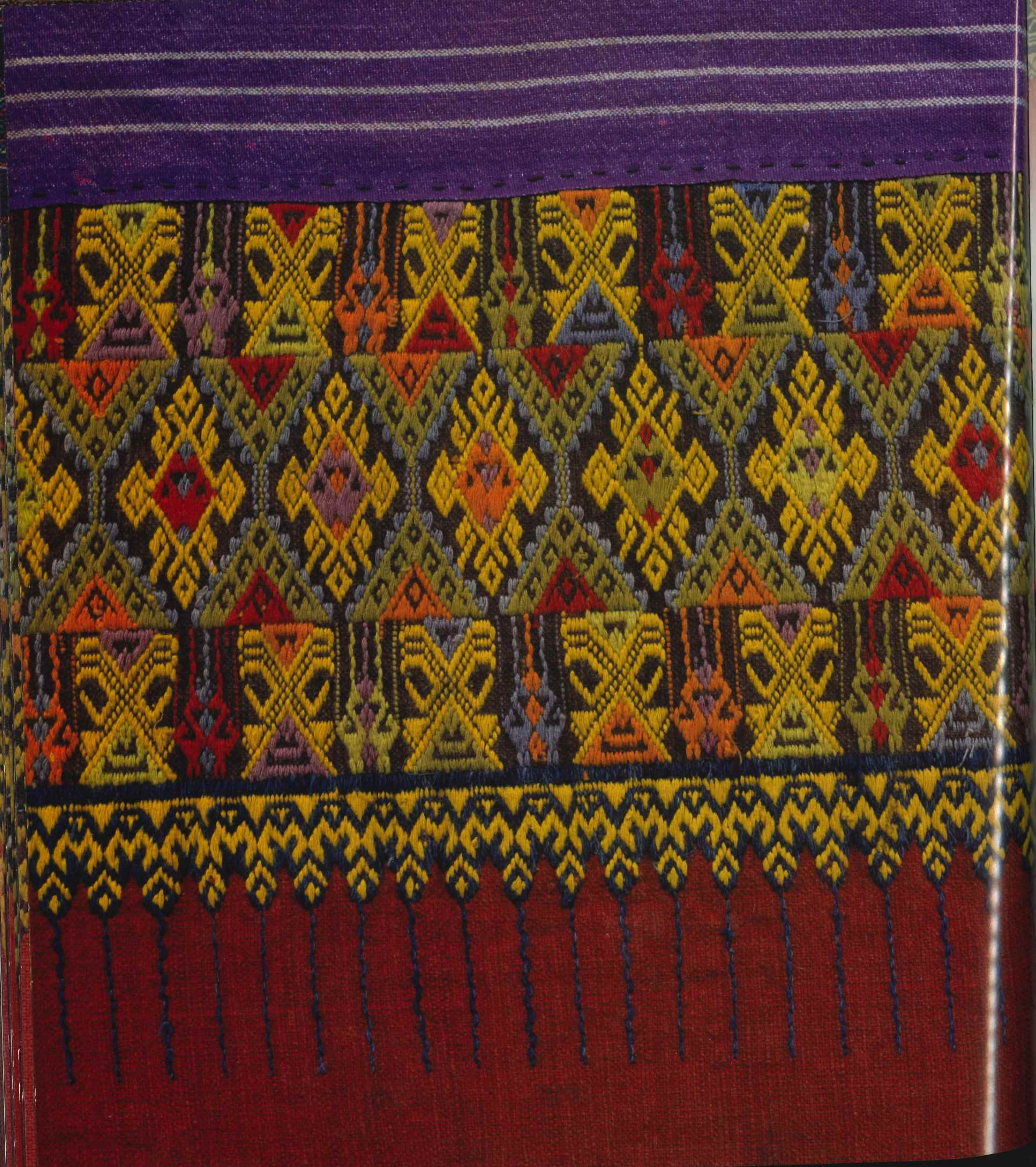

▲ ซิ่นตีนจกพบที่บ้านพวงคำ ตำบลลี้ อำเภอลี้

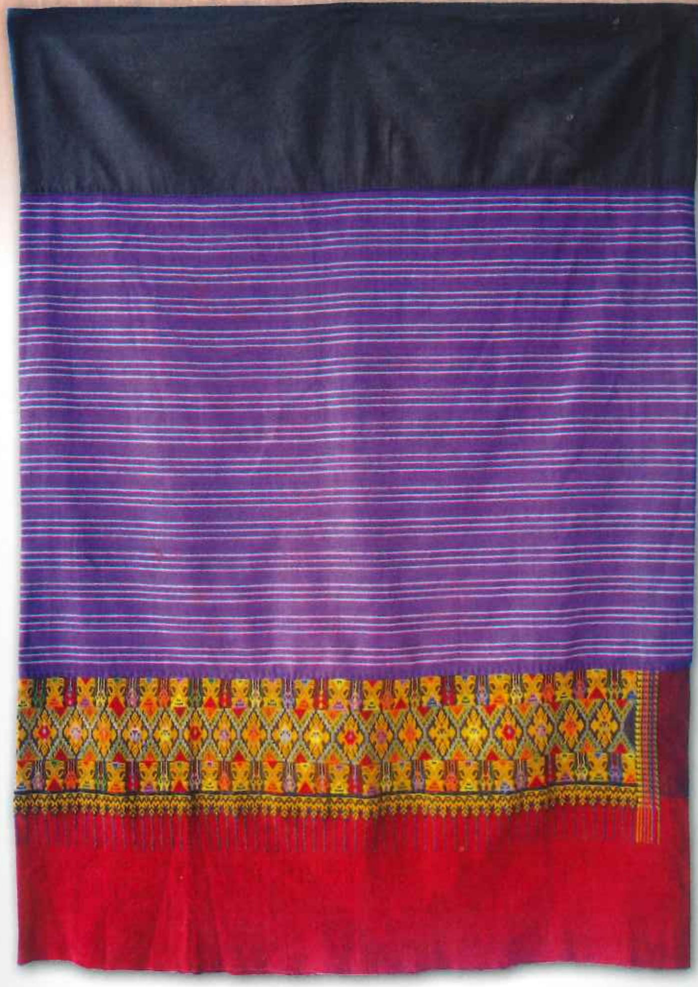

▲ ซิ่นตีนจกพบที่บ้านพวงคำ ตำบลลี้ อำเภอลี้

ตำแหน่ง
แถบบนและแถบล่าง
ขัน
โคม

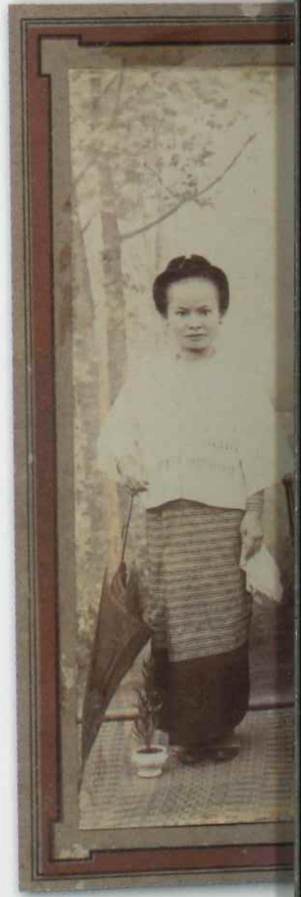

▶ ครอบครัวชาวไทยวน เวียงลำพูน (บุญยวงคอลเลคชั่น)

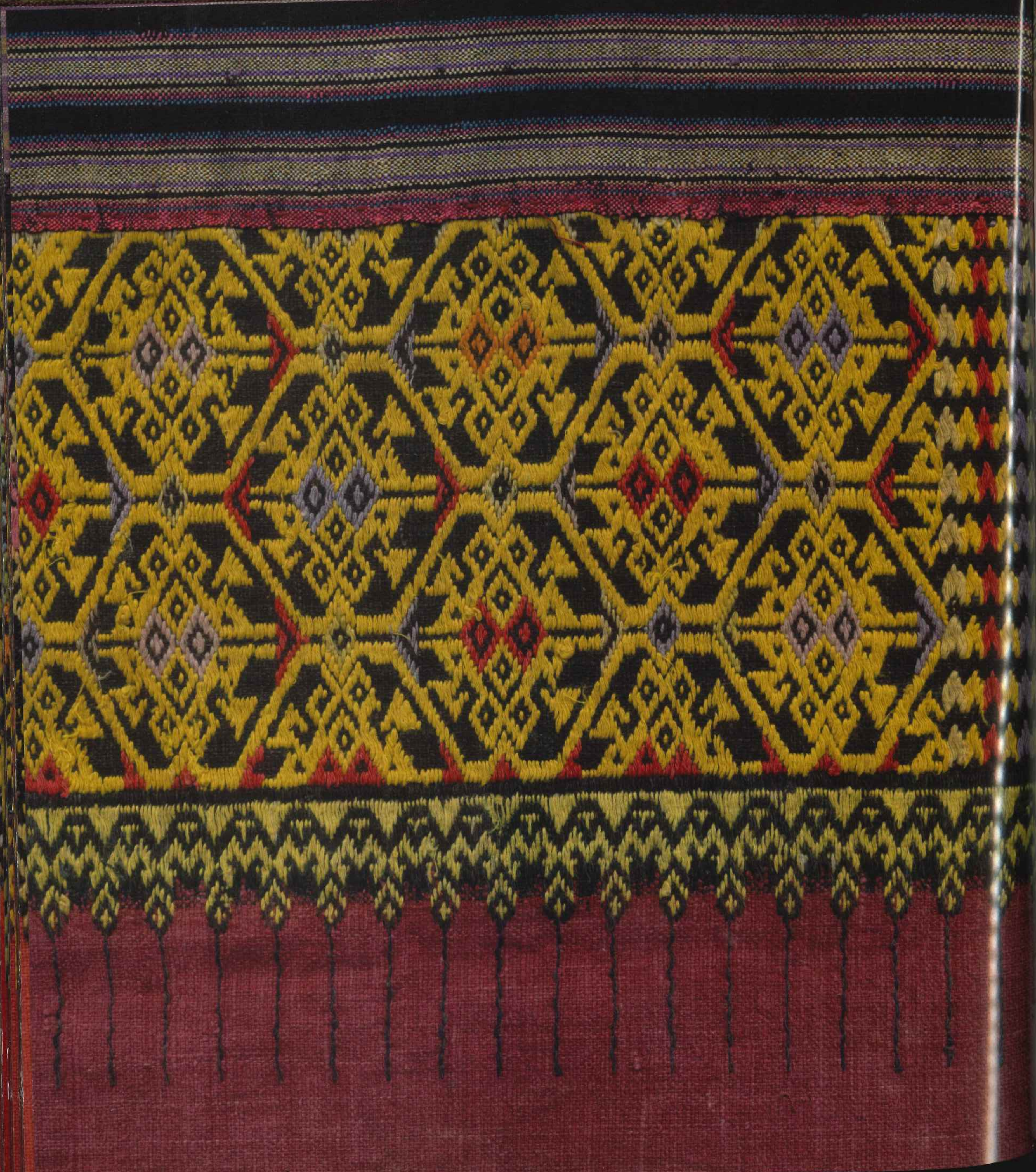

▲ ซิ่นตีนจกพบที่บ้านแม่หว่าง ตำบลนาทราย อำเภอลี้

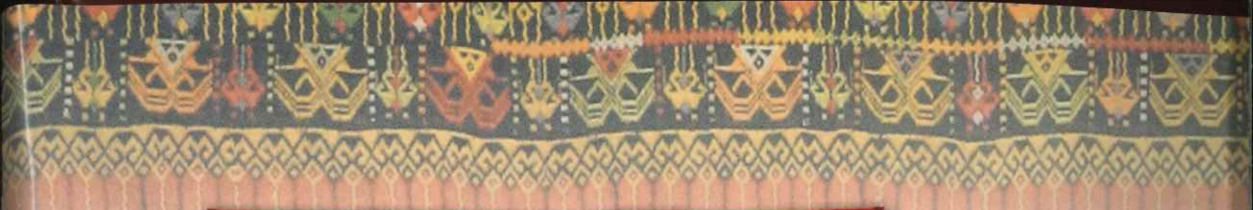

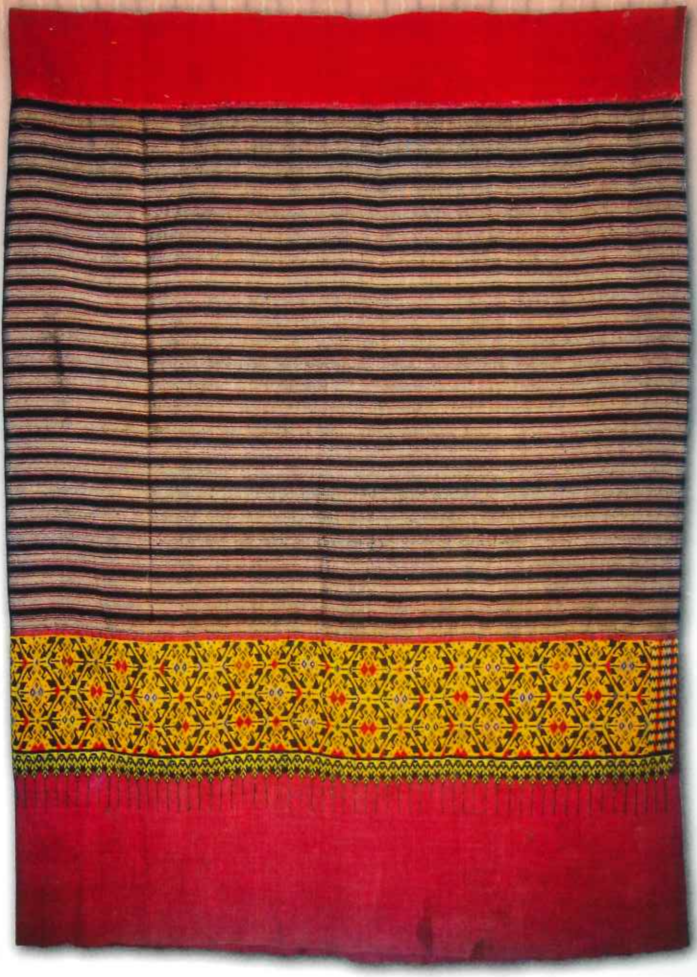

▲ ซิ่นตีนจกพบที่บ้านแม่หว่าง ตำบลนาทราย อำเภอลี้

ตำแหน่ง
แถบบนและแถบล่าง
ขัน
โคม

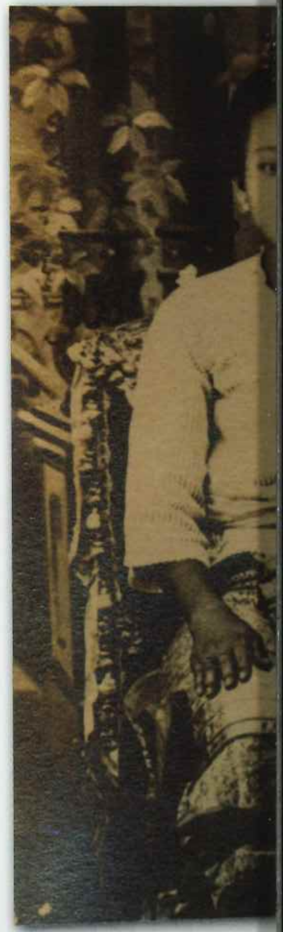

▶ สตรีไม่ทราบนาม (บุญยวงคอลเลคชัน)

บทสรุป

ในดินแดนนี้ เมื่อมองลงมาจากยอดดอยสุเทพ จะเห็นเป็นที่ราบกว้างใหญ่ในมุมกว้าง ๓๖๐ องศานั้น ล้อมรอบด้วยทิวเขา ที่ราบแห่งนี้เริ่มผายกว้างในทางทิศเหนือ ที่อำเภอแม่แตง กว้างมากในตอนกลาง แล้วค่อย ๆ สอบแคบลงที่ตอนล่างของอำเภอจอมทอง ไปสิ้นสุดที่อำเภอฮอด ที่ราบแห่งนี้คือที่ราบลุ่มน้ำแม่ปิงตอนบน และ น้ำแม่ปิงสายนี้ เมื่อไหลผ่านที่ราบอันอุดมสมบูรณ์ไปแล้วก็ไหลกัดเซาะเวียนวนอยู่ในเทือกเขาหินปูนสลับซับซ้อน การพังทลายของหินปูนก่อให้เกิดออบหรือแคนยอนที่น่าดู ตามสองฟากฝั่งแม่น้ำมีถ้ำ โพรง หน้าผาสูงชัน มีหินงอกหินย้อย สีสันรูปร่างแปลกตา มองเห็นเป็นสิ่งต่าง ๆตามมโนภาพของแต่ละคน บางแห่งมีธารน้ำไหล ตกลงมายังลำน้ำสวยงามยิ่งนัก บางแห่งซอกเขาเป็นหินผาที่แข็งแรงบีบลำน้ำให้ไหลไปเป็นลำแคบ ๆ เกิดเกาะแก่งน่าอัศจรรย์ใจ ผู้คนในอดีตใช้เส้นทางนี้ในการคมนาคม ขนส่งโดยเรือและแพ ถัดจากเมืองฮอดลงไป จะเห็นหมู่บ้านเรียงรายตามริมฝั่งน้ำ เรียงลำดับจากบ้านหน้าแก่ง วังตอน เกาะผีบ้า บ้านน้อย ผาวิ่งชู้ บ้านโท้งผาสาท บ้านท่าเดื่อ ผาหีบ บ้านท่าครั่ง บ้านชั่ง บ้านท่าคือ บ้านหนองบัวคำ บ้านดีนดอยเกิ้ง บ้านมืดกา และบ้านงิ้วเฒ่าเป็นบ้านสุดท้าย นาน ๆ จะเห็นก้อนหินใหญ่โผล่ผิวน้ำบ้าง เมื่อเริ่มเข้าเขตซอกเขา ก็จะไม่เห็นชุมชนใดอีก นอกจากผาหินปูนทางฝั่งซ้ายและผาหินเขี้ยวหนุมานทางฝั่งขวาซึ่งมีสีขาวบ้าง สีเทาบ้าง สีคล้ายสนิมเหล็กบ้าง การเดินทางจะผ่านผาเผียบ แก่งป๊อก ผาวอก ผาเต๋า แก่งอกม้า ผาฆ้อง ผาขัว ผาแจ้ก ผาสุ่มไก่ แก่งปัดตา ผาผึ้ง ผาปุ๊ และหมู่บ้านเล็ก ๆชื่อบ้านสะเลียม ต่อไปเป็น ผาม่าน หาดหยวก ดอยโหยด ผาสะเปา บ้านก้อท่า ผาเต่า แก่งก้อ ผาควาย แก่งขอน ผาผางประทีป ผาพระนอน ผาวี ผาหมู ผามัน แก่งสะเลียม แก่งปวง ผาแมว วันคืนตามเส้นทางจะมีทัศนียภาพที่งดงามมากขึ้นตามลำดับ จนถึงแก่งช้างร้อง วังสิงห์ที่มีหินเขี้ยวหนุมานโผล่พ้นน้ำเป็นรูปสิงห์ ผ่านแก่งจาน ถึงแก่งสร้อยที่ผู้เดินทางเกรงกลัวอันตรายกันมากที่สุด หลังจากนั้นก็ผ่านแก่งต่าง ๆอีกมากมาย พ้นจากสบตื๋นที่ห้วยแม่ตื๋นไหลมาบรรจบแม่ปิงก็จะผ่านแก่งอาบนางที่สวยงามมาก หลังจากนั้นก็จะเข้าเขตภูเขาหินแกรนิต ทิวทัศน์คลายความงดงามลงบ้างแล้ว แม่น้ำเริ่มกว้างออก สองฟากฝั่งไกลกันออกไปเรื่อย ๆ น้ำแม่ปิงจึงไหลต่อเข้าไปในเขตที่ราบอำเภอบ้านนา จังหวัดตากเรียกว่าที่ราบลุ่มน้ำแม่ปิงตอนล่าง เริ่มมีหมู่บ้านราษฎรอีกครั้งหนึ่ง แต่ที่เอ่ยมาเหล่านี้จมอยู่ใต้ทะเลสาบเหนือเขื่อนทั้งสิ้น เหลืออยู่ความทรงจำของผู้คนหลาย ๆคน แต่จะมีสักกี่คนที่ยังมีชีวิตเหลืออยู่ในวันนี้ ย่อมหมายความว่าคงต้องกลายเป็นตำนานในเร็ววัน อย่างไรก็ตาม ยังมีร่องรอยแห่งตำนานนี้อยู่บ้าง สิ่งนี้เรียกว่าซิ่นตีนจกแห่งลุ่มน้ำแม่ปิง ถ้าอารยธรรมหมายถึงความเจริญทางวัฒนธรรม สิ่งนี้ก็นับได้ว่าเป็นร่องรอยของอารยธรรม และชาวลุ่มน้ำแม่ปิงนี่เองเป็นผู้สร้างอารยธรรมโดยสิ่งที่อยู่เบื้องหลังคือความฉลาดทางเชาวน์ปัญญา การคิด การใช้เหตุผล การคำนวณ การเชื่อมโยง ตลอดจนสามารถปรับเปลี่ยนแนวคิดได้อย่างเหมาะสมกับสภาวการณ์ เช่นความสำนึกในเรื่องแบบสมมาตรในการรังสรรค์ผืนผ้าเป็นลวดลายเพื่อใช้ในชีวิต ผ้าตีนจกโบราณเหล่านี้แสดงถึงอดีตกาลและอัตลักษณ์ของชาติพันธุ์ไทยวนที่ยังสืบทอดมาจวบจนปัจจุบัน ถ้าจะเปรียบเทียบกับจารึกในทางประวัติศาสตร์ หรือฮาร์ดดิสก์ในเครื่องคอมพิวเตอร์ หรือดีเอ็นเอของสิ่งมีชีวิต ผ้าตีนจกโบราณเหล่านี้ก็คือสิ่งบันทึกข้อมูลสำคัญของคนไทยวน ใช้บอกเล่าความเป็นมาและจะบอกเล่าความเป็นไปแก่อนุชนรุ่นหลังต่อจากนี้ ผ้าตีนจกโบราณเหล่านี้เป็นตัวชี้วัดคะแนนในสนามประลองอัจฉริยภาพของชาติพันธุ์ไทยวนต่อชาติพันธุ์อื่น ๆ เป็นสิ่งบ่งชี้ว่าชาวไทยวนก็มีความมั่งคั่งพอมีเวลาพอและมีความสามารถพอที่จะสร้างอารยะธรรมและสืบทอดมันต่อมายังลูกหลานได้

การนับความถี่ของการใช้แบบสมมาตรในตัวอย่างลายจกบนตีนซิ่นของชาวไทยวนที่พบในเขตลุ่มน้ำแม่ปิงและแม่น้ำสาขาที่เสนอในหนังสือเล่มนี้ พบว่าแบบสมมาตรทั้งหมดถูกใช้ปะปนกันไป จากการวิเคราะห์จำแนกแบบสมมาตรของลวดลายซิ่นตีนจกจากบุญยวงคอลเลคชันพบว่า ซิ่นตีนจกจอมทอง ๑๑ ชิ้น มีการใช้แบบสมมาตรทั้งหมดเพียง ๓ แบบได้แก่ *pm11 pmm2* และ *pma2* ทั้งนี้สัดส่วนของความถี่ในการใช้แบบสมมาตร แสดงในตาราง

สัดส่วนการใช้ (%)	*p111*	*p112*	*p1m1*	*p1a1*	*pm11*	*pmm2*	*pma2*	รวม
แถบบน ล่าง	-	-	-	-	๙๑	-	๙	๑๐๐
ขัน	-	-	-	-	-	๑๐๐	-	๑๐๐
โคม	-	-	-	-	๘๒	๑๘	-	๑๐๐

ซิ่นตีนจกแม่หาดทั้ง ๑๓ ชิ้น มีการใช้แบบสมมาตรเพิ่มมาเป็น ๔ แบบได้แก่ *p111 pm11 pma2* และ *pmm2* ทั้งนี้สัดส่วนของความถี่ในการใช้แบบ สมมาตร แสดงในตาราง

สัดส่วนการใช้ (%)	*p111*	*p112*	*p1m1*	*p1a1*	*pm11*	*pmm2*	*pma2*	รวม
แถบบน ล่าง	-	-	-	-	๖๐	-	๔๐	๑๐๐
ขัน	๑๐	-	-	-	-	๙๐	-	๑๐๐
โคม	-	-	-	-	๙๒	๘	-	๑๐๐

ซิ่นตีนจกบ้านตาลหลวง ทั้ง ๒๒ ชิ้น มีการใช้แบบสมมาตรเพิ่มมาเป็น ๕ แบบได้แก่ *p111 p1a1 pm11 pmm2* และ *pma2* ทั้งนี้สัดส่วนของความถี่ในการใช้แบบสมมาตรแสดงในตาราง

สัดส่วนการใช้ (%)	*p111*	*p112*	*p1m1*	*p1a1*	*pm11*	*pmm2*	*pma2*	รวม
แถบบน กลาง ล่าง	๖	-	-	๓๓	๕๖	-	๖	๑๐๐
ขัน	-	-	-	-	-	๑๐๐	-	๑๐๐
โคม	-	-	-	-	๑๐๐	-	-	๑๐๐

เช่นเดียวกับซิ่นตีนจกหนีน้ำท่วม เมืองฮอดจำนวน ๓๐ ชิ้น มีการใช้แบบสมมาตรจำนวน ๕ แบบได้แก่ *p111 p1a1 pm11 pmm2* และ *pma2* ทั้งนี้สัดส่วนของความถี่ในการใช้แบบสมมาตร แสดงในตาราง

สัดส่วนการใช้ (%)	*p111*	*p112*	*p1m1*	*p1a1*	*pm11*	*pmm2*	*pma2*	รวม
แถบบน กลาง ล่าง	๔	-	-	๔	๓๖	-	๕๖	๑๐๐
ขัน	๔	-	-	-	๔	๙๒	-	๑๐๐
โคม	-	-	-	-	๘๗	๑๓	-	๑๐๐

ท้ายที่สุดที่น่าชื่นชมความสามารถในการออกแบบของช่างจกโหล่งลี้ก็คือการได้พบแบบสมมาตรทั้ง ๗ แบบอย่างครบถ้วนในซิ่นตีนจกโหล่งลี้จำนวน ๒๙ ชิ้น แสดงในตาราง

สัดส่วนการใช้ (%)	*p111*	*p112*	*p1m1*	*p1a1*	*pm11*	*pmm2*	*pma2*	รวม
แถบบน กลาง ล่าง	๑๐	๓	๓	๑๓	๓๒	๗	๓๒	๑๐๐
ขัน	-	-	-	-	-	๑๐๐	-	๑๐๐
โคม	-	-	-	-	๖๘	๓๒	-	๑๐๐

โดยรวมแล้วเราจะเห็นว่ามีการใช้แบบสมมาตรครบทั้ง ๗ แบบในการจกลวดลายบนตีนซิ่นของชาวไทยวนลุ่มน้ำแม่ปิง ทั้งนี้ความหลากหลายของการใช้แบบสมมาตร หรืออีกนัยหนึ่งก็คือความหลากหลายในรูปแบบของการสร้างลายพบที่แถบบนล่าง หรือ แถบกลาง(ถ้ามี) ของตีนจก สิ่งที่น่าสังเกตก็คือ แม้แบบสมมาตร *p111* และเป็นแบบที่ง่าย กล่าวคือเป็นเพียงการจกลายเดิมไปวางซ้ำไม่มีการพลิกแพลง หมุน หรือสลับใดๆ กระนั้นก็พบการใช้แบบสมมาตรนี้น้อยครั้งกว่าแบบสมมาตรอื่นที่ยากกว่าเช่น *p1a1* หรือ *pmm2* ซึ่งพบว่าถูกใช้บ่อยครั้งกว่ามากอย่างมีนัยสำคัญ ข้อสังเกตที่ได้เหล่านี้ สุดท้ายก็ยังคงเป็นข้อสังเกตไม่อาจสรุปอะไรได้เพราะว่าไม่มีผู้ใดทราบถึงเหตุผลของช่างจกผืนผ้าเหล่านี้ แท้ที่จริงการจะใช้แบบสมมาตรซึ่งยากหรือง่ายแบบไหน การใช้ต้นแบบหรือแม่ลายอย่างง่ายหรือซับซ้อนแค่ไหน เรามิอาจหยั่งรู้ในเหตุผลนั้นได้ สิ่งที่เราเห็นกันนั้นอาจเกิดจากหลายๆปัจจัย เช่นสังเกตว่ามีการจกเป็นรูปหงส์สีดำหรือสีเข้มอื่นๆบนพื้นสีดำเหมือนกันอยู่บ่อยๆ ที่รู้จักกันในนามของ "ลายหงส์ดำ" แต่ด้วยเหตุผลใดเล่าจึงต้องเป็นหงส์ดำ ทั้งๆที่มีสีอื่นให้เลือกใช้ โดยทฤษฎีการใช้สีง่ายๆช่างจกย่อมต้องเลือกสีเส้นจกให้ต่างจากสีพื้นเพื่อให้เห็นเป็นลวดลายเด่นชัดแต่เราอธิบายไม่ได้ ที่ทำได้ก็แค่คาดเดาความคิด นักวิชาการทางด้านผ้าโบราณหรือนักสะสมหลายๆท่านพยายามตีความ คาดเดา แล้วยกข้อคาดเดาของตนเองให้เป็นความจริงหรือที่เรียกกันว่าทฤษฎีเป็นซึ่งสิ่งที่ไม่กระทำกันในทางคณิตศาสตร์ เมื่อเห็นซิ่นตีนจกลวดลายแปลกๆชิ้นหนึ่ง หลายๆ ท่านกล่าวสรุปว่าเป็นสันป่าตองบ้าง เป็นลำปางบ้าง เป็นที่นั่นบ้าง เป็นที่นี่บ้าง ถ้าจะตั้งคำถามว่าต่อท่านเหล่านั้นว่าท่านเคยพบช่างสันป่าตองนั่งกี่กำลังจกตีนซิ่น "แบบสันป่าตอง" ที่ท่านกำลังกล่าวอ้างนั้นกี่ครั้งในชีวิตของท่าน หรือท่านเคยเห็นผู้หญิงไทยวนชาวสันป่าตองนุ่งซิ่นตีนจก"แบบสันป่าตอง"ที่ท่านกำลังกล่าวอ้างนั้นกี่คนหรือกี่ครั้งในชีวิตของท่าน เชื่อว่าท่านตอบไม่ได้ แต่เหตุไฉนท่านเหล่านี้จึงมักยกข้อคาดเดามาเป็นทฤษฎี ในทางคณิตศาสตร์พวกเราไม่เชื่อ แม้ท่านจะตอบว่าท่านเห็นมามากกับตาตนเอง นักคณิตศาสตร์ก็ไม่เชื่อ เราเห็นว่านกแก้วบินได้ นกกระปูดบินได้ นกเขาบินได้ นกเอี้ยงบินได้ นกกระจอกบินได้ นกพิราบบินได้ นกสารพัดชนิดบินได้ จะมีตัวอย่างมากแค่ไหนก็ตามนักคณิตศาสตร์มิอาจสรุปเป็นทฤษฎีได้ว่านกทุกชนิดต้องบินได้ นักคณิตศาสตร์เราไม่ยอมรับการสรุปผลแบบ อุปนัย สิ่งที่จะทำได้ก็คือแค่การรายงานข้อมูลและตั้งข้อสังเกตไว้เพียงเท่านี้ ขอให้ผู้อ่านไตร่ตรอง
